# Discovery and Development of a Potent LIMK2 Isoform-Specific Degrader

**DOI:** 10.1021/acschembio.6c00137

**Published:** 2026-05-01

**Authors:** Kamal Rayees Abdul Azeez, Hayuningbudi Saraswati, Thorsten Mosler, Thomas Hanke, Hung Ho-Xuan, Noah Neder, Saran Aswathaman Sivashanmugam, Marcel Heinz, Martin-Peter Schwalm, Giulio Giuliani, Rajeshwari Rathore, Rubina Kazi, Rahul Kumar, Marko Mitrovic, Cristian Prieto-Garcia, Henry J. Bailey, Sebastian Mathea, Gerhard Hummer, Ivan Đikić, Susanne Müller, Alexandra Stolz, Daniela S Krause, Stefan Knapp

**Affiliations:** 1 Institute of Pharmaceutical Chemistry, 9173 Goethe University, Max-von-Laue-Str. 9, 60438 Frankfurt am Main, Germany; 2 Structural Genomics Consortium (SGC), Buchmann Institute for Life Sciences, Max-von-Laue-Str. 15, 60438 Frankfurt am Main, Germany; 3 Institute of Transfusion Medicine−Transfusion Centre, Johannes Gutenberg University Medical Center, 55131 Mainz, Germany; 4 Institute of Biochemistry II, School of Medicine, Goethe University Frankfurt, Frankfurt am Main 60590, Germany; 5 Buchmann Institute for Molecular Life Sciences (BMLS), Goethe University, Max-von-Laue-Str. 15, 60438 Frankfurt am Main, Germany; 6 Department of Theoretical Biophysics, 28273 Max Planck Institute of Biophysics, Frankfurt am Main 60438, Germany; 7 Institute for Biophysics, Goethe University Frankfurt, Frankfurt am Main 60438, Germany; 8 German Cancer Consortium (DKTK) Mainz/Frankfurt, German Cancer Research Center (DKFZ), 69120 Heidelberg, Germany; 9 Research Center for Immunotherapy (FZI), University Medical Center, University of Mainz, 55131 Mainz, Germany

## Abstract

The LIM kinases (LIMK1/2) are key mediators in signaling cascades that regulate actin cytoskeleton dynamics via cofilin phosphorylation. Dysregulation of these pathways and overexpression of LIMKs are implicated in disease development, including cancer, Fragile X syndrome, and glaucoma. Positioned downstream of actin-regulating Rho GTPase signaling pathways, LIM kinases are attractive drug targets. Here, we targeted LIMKs with PROTACs to disrupt both catalytically and noncatalytically mediated functions. Despite employing a dual LIMK1/2 inhibitor warhead and high structural conservation between the two human LIM kinases, we discovered isoform-specific LIMK2 degradation by initial PROTACs that we optimized into a highly potent and selective LIMK2 degrader. Cell-based assays and structural analysis indicated that isoform specificity was likely driven by favorable orientation bias and/or lysine accessibility, along with enhanced ternary complex formation. We comprehensively characterized the PROTAC as a chemical probe that induces isoform-specific degradation, offering a powerful alternative to conventional reversible pan-LIMK inhibitors.

## Graphical abstract

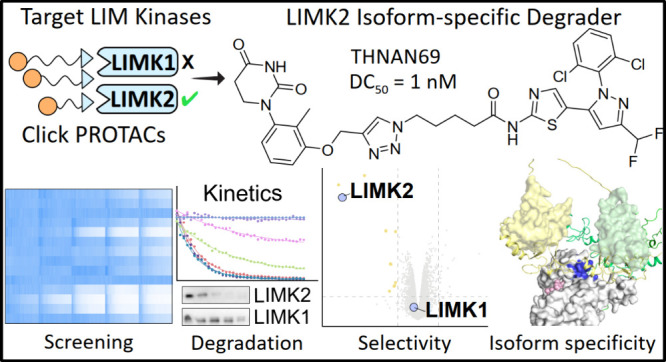

## Introduction

The LIM kinases (LIMKs), LIMK1 and LIMK2, are dual-specific serine/threonine kinases. Each of these key signaling enzymes contain two LIM domains and a PDZ domain at their N-termini, followed by proline/serine-rich regions and a C-terminal kinase domain.^1^ The precise functions of the LIM and PDZ domains have not been firmly established, but biochemical studies suggest that these protein interaction domains may have roles in LIMK autoregulation, scaffolding in LIMK signaling complexes, and cellular localization.^2,3^ The LIMKs are key mediators of multistep signaling pathways and are activated via phosphorylation of their activation loops by RhoA-ROCK1/2, Rac/Cdc42-PAK1/2/4, Cdc42-MRCKα, and Ca^2+^-CaMKIV signaling cascades.^4,5^ Upon activation, LIMKs phosphorylate Ser 3 of the highly conserved actin-depolymerizing factors (ADFs), including cofilins (CFL1, CFL2) and destrin (DSTN). Phosphorylated cofilins no longer bind F-actin filaments, which maintain actin polymerization and contribute to actin cytoskeleton dynamics. Disruption of these signaling pathways and overexpression of LIMKs have been reported to contribute to disease development by dysregulating the actin cytoskeleton. Currently, LIMK dysregulation is strongly linked to cancer metastases in diverse tumor types.^6,7^

The best studied substrate of LIMKs are cofilins, key regulators of actin cytoskeleton dynamics. The Rho signaling pathways stringently regulates cofilin and LIMKs are direct modulators of cofilins. Due to their central role in this signaling network and their ability to modulate actin dynamics and cell motility, LIMKs have been identified as attractive drug targets.^1,8^ Consequently, several selective LIMK inhibitors and chemical probes have been developed including type I, type II and type III kinase inhibitors.^9–12^ However, data reported in the literature on phospho-cofilin (pCFL) levels after treatment with selective pan LIMK inhibitors show generally modest effects of cofilin phosphorylation suggesting that LIMKs are not the only kinases that phosphorylate cofilins.^12–15^ Although both LIMK1 and LIMK2 have been reported to be deregulated in diseases, an increasing number of reports have identified LIMK2-driven dysregulation in cancers^6^ and glaucoma.^16^ Developing isoform-specific inhibitors is challenging due to the high structural similarity between the two LIMK isoforms. Recently, our laboratory targeted a LIMK1-specific cysteine residue (C349) in the glycine-rich loop to develop a LIMK1-selective covalent chemical probe.^17^ However, there is no cysteine residue in LIMK2 that could be used for isoform-selective targeting. Although a sulfonamide-based type III inhibitor was initially reported to be LIMK2-specific,^18^ the developed inhibitor, and more potent derivatives such as the chemical probe TH257, lack isoform specificity when evaluated comprehensively in enzyme kinetic and cellular target engagement assays.^9,11^ Conventional targeting strategies have not yet succeeded in developing a selective chemical probe for the LIMK2 isoform that could be used to study its function in cellular environments.

Proximity-induced degradation to disrupt both catalytic and noncatalytic mediated functions and regulation represents a promising strategy to target the LIMKs. Proteolysis-targeting chimeras (PROTACs) are bifunctional molecules that initiate ubiquitin-mediated degradation of target proteins through proximity, utilizing a target-binding and an E3 ligase-binding warhead connected by a linker.^19^ Currently, 26 PROTAC degraders have been reported to have entered clinical testing, representing 12 target families (including 4 kinases), primarily for cancer therapy.^20,21^ An increasing number of PROTACs are also being used for target identification and validation,^21–23^ and as chemical probes.^24–26^

PROTACs developed using promiscuous kinase ligands identified LIM kinases as highly degradable.^27^ Recently, a TH257-based PROTAC achieved weak degradation of LIMK2. However, this degrader required high concentrations (10 μM) and long treatment times (72 h).^28^ Here, we describe the development of a highly potent (DC_50_ = 1 nM), efficacious (*D*_max_: 88% at 10 nM), and proteome-wide isoform-selective LIMK2 PROTAC. We rationalized the observed exclusive isoform-specific LIMK2 degradation through cellular ternary complex formation assays and structural models supported by molecular dynamics (MD) simulations.

## Results

### Click Chemistry Enabled Efficient Assembly of Bifunctional LIMK PROTACs

For the synthesis of bifunctional PROTAC molecules, we used LIMKi3, an orthosteric pan-LIMK type I inhibitor, as the target-binding moiety, and applied a click chemistry strategy for linker and E3 ligase ligand attachment.^29^ Alkyl and triazole-containing linker moieties have been routinely used, making up 44.8% and 12.1% of the current PROTAC linker landscape, respectively.^30^ Copper-catalyzed azide–alkyne cycloaddition (CuAAC) reactions rapidly generated bifunctional PROTACs containing triazole-alkyl linkers of varying lengths. We chose LIMKi3 as it is a highly potent and selective dual LIMK1/2 inhibitor with a known binding mode (Figure 1A,B).^31^ The solvent-exposed isobutyramide moiety served as an ideal exit vector attachment point, enabling us to successfully generate LIMKi3 building blocks with terminal azide moieties for attachment of alkyne-functionalized E3 ligands. The PROTAC design strategy outlined in Figure 1C included both von Hippel–Lindau (VHL) and cereblon (CRBN) recruiting PROTACs. The LIMKi3 building block was synthesized as previously described^17,31^ (Scheme S1), while the E3 handles and the final PROTACs were synthesized according to Schemes S2 and S3. A detailed evaluation of the physicochemical properties of all synthesized PROTACS is summarized in Figure S1.

**Figure 1:**
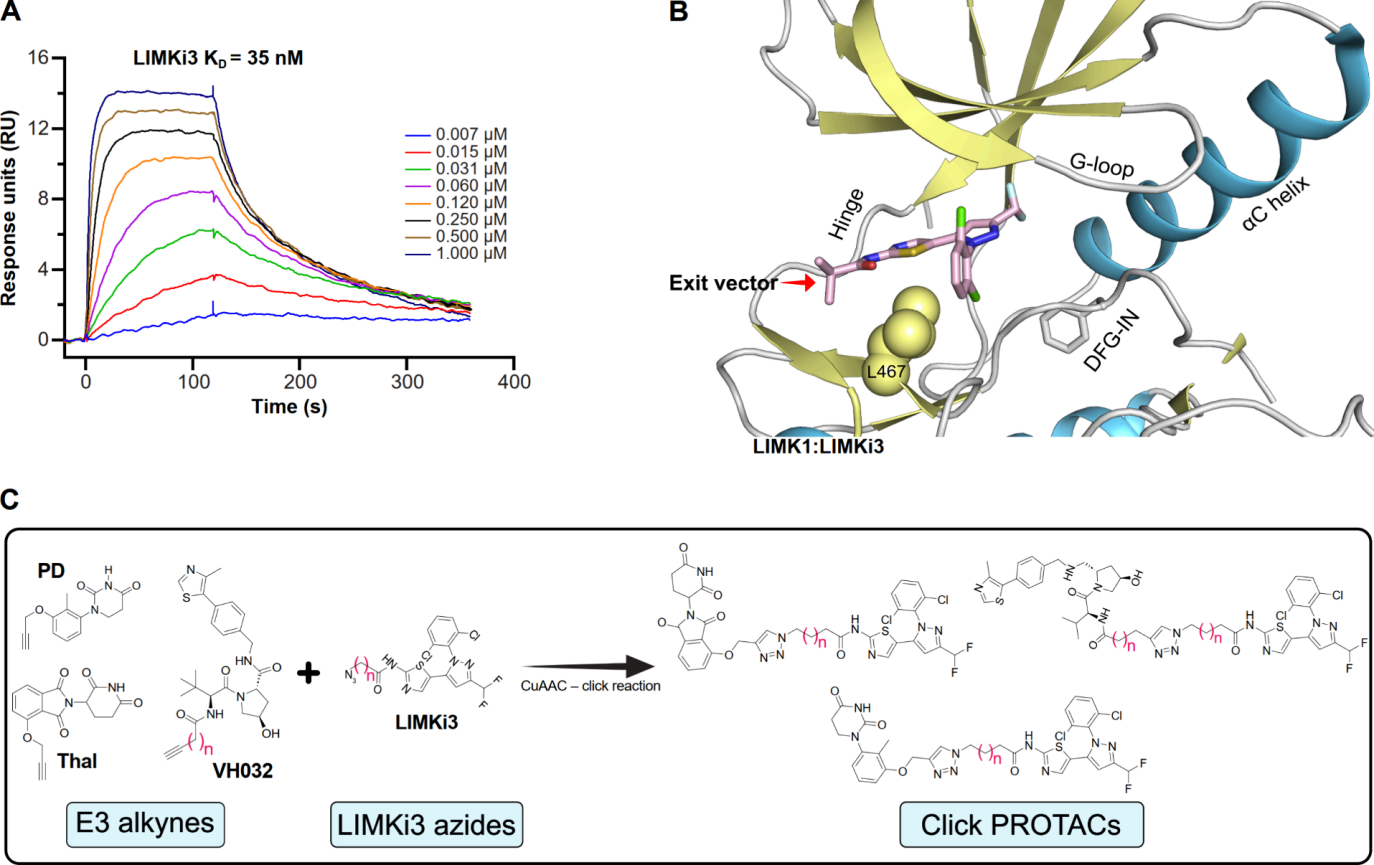
PROTACs warhead selection and synthesis strategy. (A) Binary complex affinity sensorgram between LIMK1 and LIMKi3, determined by surface plasmon resonance (SPR). Sensorgram at various concentrations of the compound is shown. (B) Co-crystal structure of LIMKi3 bound to LIMK1 (PDB: 8AAU). LIMKi3 (light pink) stabilizes LIMK1/2 in a type I kinase-binding configuration. The exit vector for PROTAC linker attachment is indicated (red arrow), and key structural elements are labeled. (C) Combinatorial synthetic strategy for LIMK-targeting PROTAC development. A copper-catalyzed click reaction linked the alkyne-containing E3 ligase ligands with the azide-functionalized LIMKi3 warhead, yielding LIMK PROTACs with variable triazole-alkyl linker lengths.

### CRBN-PROTACs Exhibit Strong Binary Target Affinities

To ensure that our linker attachment strategy did not compromise LIMKi3 inhibitor potency for LIMKs, formation of binary complexes was characterized in vitro using differential scanning fluorimetry (DSF) and SPR, and in live cells using the nanobioluminescence resonance energy transfer (NanoBRET) assay. The LIMK2 kinase domain is poorly expressed and unstable in vitro; hence, LIMK2 binary complex characterization was limited to NanoBRET analysis. Binary complex characterization across the three assay platforms is summarized in Figure 2A and Figure S2A–C.

**Figure 2:**
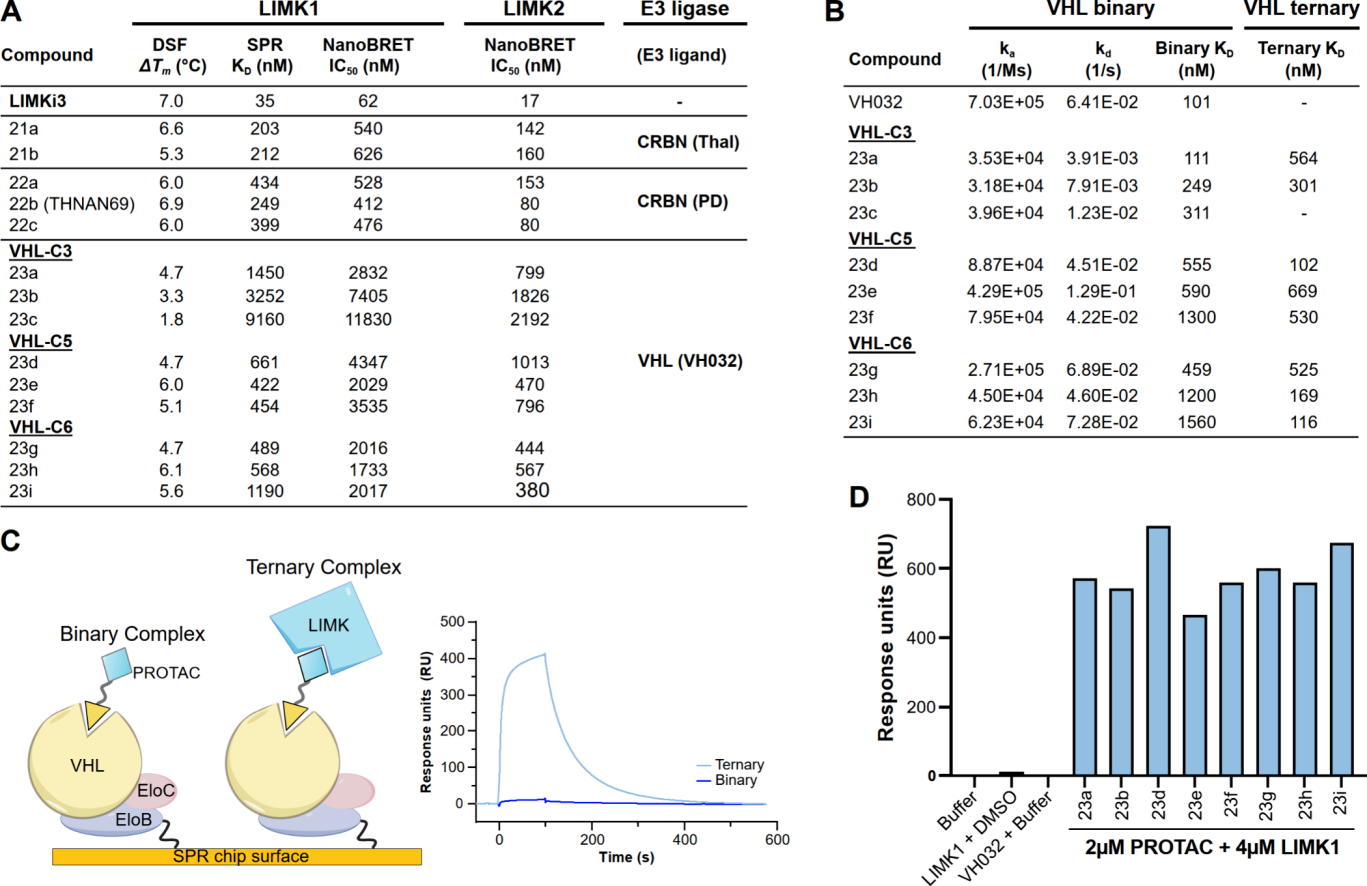
**Biophysical Characterization and Cellular Target Engagement of the Synthesized PROTACs. (A)** Binary complex characterization. Mean DSF, SPR, and NanoBRET binding values relative to LIMKi3 are summarized. All PROTACs showed reduced binary binding. (B) Binding kinetics of VHL-recruiting PROTACs. Binary and ternary complex affinities were determined as outlined in Figure 2C. Binary complex kinetics and affinities, as well as ternary complex affinities, are shown. Ternary complex kinetics could not be fitted to a 1:1 binding model, likely due to excess LIMK1 added to prevent the hook effect. (C) Graphical representation of the VHL ternary complex assay by SPR. Biotinylated VHL:ELOB:ELOC was immobilized onto a streptavidin-coated SPR chip. Ternary complex formation upon passing the binary complex over the surface increases the signal due to the higher molecular weight. (D) Ternary complex formation of VHL PROTACs by SPR. Steady-state sensorgram responses were quantified, plotted as a bar graph, and compared with controls (buffer, VH032, and LIMK1-DMSO).

All five CRBN-based PROTACs showed temperature shifts (Δ*T*_*m*_) in the DSF assay comparable to LIMKi3 (7.0 °C). However, determination of binding constants showed about a 5- to 10-fold decrease in affinity for both LIMK1 and LIMK2 by SPR and/or NanoBRET. In the NanoBRET assay, all the PROTACs maintained a 3.5-fold IC_50_ difference between LIMK1 and LIMK2, except for **22b** (THNAN69) and **22c**, which showed more potent IC_50_ values (80 nM). Additionally, the CRBN PROTACs exhibited similar target engagement in both digitonin-permeabilized and intact cells, indicating good cell permeability (Figure 2A and Figure S2B). In contrast, all 9 VHL PROTACs consistently showed poor target engagement across all assays compared to LIMKi3, and thus, only weakly associated with LIMKs, compared to the CRBN PROTACs. Comparison of NanoBRET data in intact and digitonin-permeabilized cells indicated poor cell penetrance of VHL–LIMK PROTACs (Figure S2B).

Surprised by the weak target engagement of VHL-based LIMK PROTACs, we investigated whether they form a stable ternary complex. To address this, we established an SPR-based ternary complex assay using immobilized VHL:ELOB/C complex on an SPR chip. In this assay format, the binary complex was formed by preincubating PROTACs with a 2-fold excess of purified LIMK1 kinase domain to avoid the hook effect. The binary complex was passed over an SPR surface immobilized with the VHL:ELOB/C complex. Ternary complex formation was detected as a large signal increase, as expected for the large molecular weight increase upon binding of the LIMK1 PROTAC complex (Figure 2C). In contrast, a lack of ternary complex formation resulted in a poor or undetectable signal.

Notably, despite their poor binary target engagement with LIMK1, all VHL-based PROTACs formed ternary complexes when screened at 2 μM PROTAC concentration (Figure 2D). However, our SPR assay was established using the LIMK1 kinase domain; therefore, we cannot entirely rule out the potential impact of the LIMK N-terminus on ternary complex formation. The VHL parent ligand, VH032, bound to VHL with an affinity of 101 nM and was used for comparison. Interestingly, PROTACs with shorter linker length flanking the triazole on the VHL side (VHL-C3) showed slower binary kinetics toward VHL. The PROTACs showed comparable binary affinity toward VHL, except for those with longer linkers – **23i**, **23h,** and **23f**, all of which exhibited affinities greater than 1 μM. Interestingly, these 3 PROTACs showed improved affinity upon formation of ternary complexes; in particular, **23i** and **23h** exhibited a 10-fold higher affinity. The long linker likely stabilized the ternary complex by forming additional interactions with LIMK1, thereby enhancing its stability (see Figure 2B).

### Degradability Screen Revealed Isoform-Specific LIMK2 Degradation by CRBN-based PROTACs

The human cholangiocellular carcinoma cell line HuCCT1, expressing both LIMKs, was chosen for an initial cellular screening of degradation efficiency by Western blot. Initially, two CRBN PROTACs, **21a** and **21b**, harboring a shorter and a longer linker, respectively, were tested across a wider concentration range to assess degradability and determine the most suitable concentrations for screening all PROTACs. We selected a treatment time of 6 h because short PROTAC incubation times are unlikely to result in significant cell death when protein levels are assessed.^32^ Interestingly, in this initial evaluation, PROTAC **21b** with the longer linker showed strong degradation of LIMK2 but notably not LIMK1, indicating isoform-specific degradation. In contrast, the short-linker analog **21a** did not degrade either isoform (Figure 3A). Encouraged by these results, we evaluated the selectivity of **21b** using quantitative global proteomics in HuCCT1 cells treated with 500 nM of the PROTAC. In agreement with our Western blot data, **21b** induced strong degradation of LIMK2, while LIMK1 protein levels remained unaffected (Figure 3B). Aurora A, a highly degradable kinase^33^ was identified as an off-target even though the ligand showed no measurable activity against this kinase.^11^ A few thalidomide-associated neo-substrates from the zinc-finger transcription factor family (ZFP91 and ZNF692) were degraded by **21b**, presumably due to its molecular glue activity. Additionally, deoxyguanosine kinase (DGUOK) was significantly downregulated, and a small number of proteins showed slightly elevated expression levels. Screening of **21b** in a second cell line, MOLT-4, revealed similar off-targets. Notably, the RING-type E3 ubiquitin ligase RNF166, along with the known CRBN-associated neo-substrates IKZF1 and IKZF3, was also found to be downregulated (Figure S3A). To reduce the risk of neosubstrate degradation, which is often observed with CRBN ligands based on thalidomide and lenalidomide, we used a recently published phenyl dihydrouracil (PD) ligand.^34^ An additional advantage of these achiral ligands is that they do not undergo isomerization; however, only a few PD-based PROTACs have been reported so far. Inspired by the PD handle, 2-methyl substituted phenyl dihydrouracil-based CRBN recruiting PROTACs were synthesized with one short and two long linker moieties attached at position 3 of the phenyl ring. We further synthesized a series of VHL-recruiting PROTACs to assess whether they also maintain isoform-specific degradation. Interestingly, none of the VHL-based PROTACs induced degradation of both LIMK isoforms, except for PROTAC **23c**, which exhibited modest LIMK2 degradation at 1 μM. Encouragingly, PROTAC **22b** (THNAN69), which contains a PD ligand, demonstrated strong LIMK2-selective degradation in Western blots (Figure 3C,D). However, although the intensities of the CFL bands varied slightly between the different lanes of the blot, the ratio of CFL to pCFL remained constant, suggesting that the levels of pCFL were unaffected when LIMK2 was selectively degraded. Our data also indicated that the dual LIMK inhibitor LIMKi3-based warheads used in PROTAC development did not enzymatically inhibit LIMKs at the tested concentrations. These data also suggested that LIMKs are redundant in regulating CFL phosphorylation levels.

**Figure 3:**
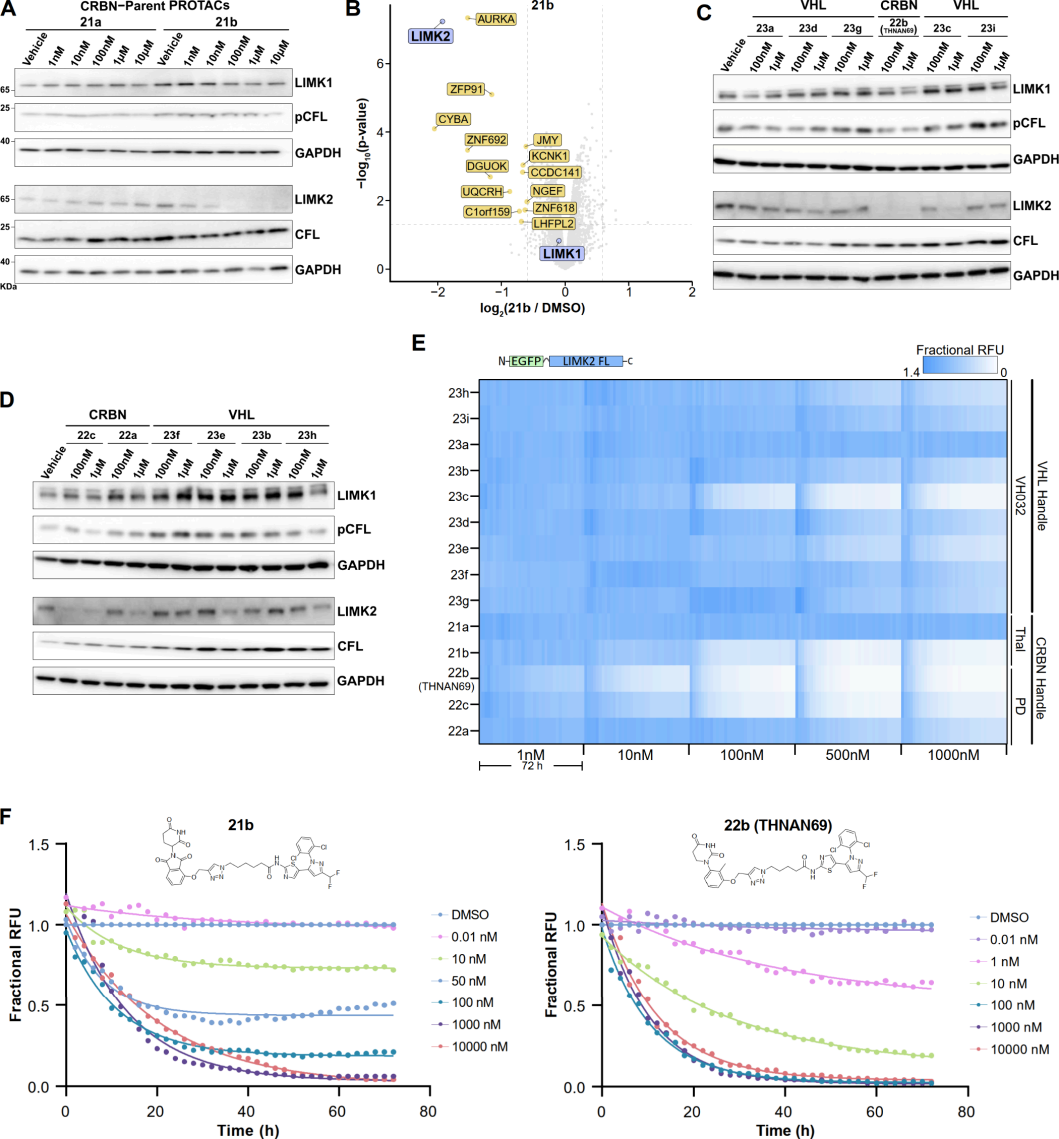
Screening and initial assessment of the degradation potency of PROTACs in HuCCT1 Cells. (A) LIMK1/2 Western blot analysis following 6 h PROTAC treatment across an extended concentration range. Degradation of both LIMK isoforms was assessed, and levels of the LIMK substrate cofilin were also monitored as phosphorylated cofilin (pCFL) and total cofilin (CFL). GAPDH served as a loading control. (B) Quantitative global proteomics of 21b following 6 h treatment. A volcano plot of differential protein abundance versus the DMSO control revealed isoform-specific degradation of LIMK2. LIMKs are highlighted in purple, while other significantly downregulated proteins are highlighted in yellow. (C,D) Western blot analysis of PROTACs screened after 6 h at 100 nM and 1 μM. In this series, compound 22b (THNAN69) induced the most potent degradation of LIMK2, while LIMK1 protein levels remained unaffected. (E) Heat map of EGFP–LIMK2. Fluorescence data were normalized to DMSO/mCherry controls and visualized to show LIMK2 degradation across all PROTACs. PROTACs were screened at five concentrations, and LIMK2 degradation was monitored in live cells over 72 h. (F) Degradation kinetics of the thalidomide-based PROTAC 21b and the PD-based PROTAC 22b (THNAN69). EGFP–LIMK2 fluorescence was monitored over 72 h as triplicates, and mean degradation curves were fitted using a one-phase exponential decay model.

To enable a more comprehensive and rapid evaluation of the synthesized PROTACs, we generated a stable, doxycycline-inducible HuCCT1 cell line that expressed an N-terminally fused EGFP–LIMK2 protein. This cell line enabled real-time monitoring of LIMK2 degradation in a time and concentration-dependent manner in living cells. The degradation activity of the synthesized PROTACs set was screened at five concentrations ranging from 1 nM to 1000 nM over 72 h using live-cell imaging (Figure 3E). These data enabled accurate determination of DC_50_ values, the assessment of maximum degradation levels (*D*_max_), as well as the time required to reach *D*_max_.

Consistent with the Western blot screen, our initial thalidomide-based PROTAC, **21b,** showed strong degradation potency at 100 nM. Transfer of the identical linker to the PD ligand system (PROTAC **22c**) further improved potency, while shortening its linker from six to five carbons led to the most potent PROTAC of this series, **22b** (THNAN69). The impact of linker length on degradation potency is summarized in Figure S3B. The structure–activity relationship (SAR) of PROTACs is often very steep, and small changes in linker length and composition can significantly affect PROTAC potency. Also in our attempts optimizing LIMK2 degradation activity we observed a dramatic effect of PROTAC activity reducing the linker from six to five carbons significantly improved potency. This is likely due to the optimal spacing and orientation of E3 ligase in the ternary complex. Real-time monitoring of EGFP–LIMK2 across an extended concentration range confirmed the improved potency of **22b** (THNAN69) compared to **21b** (Figure 3F). These data revealed that **21b**, as well as **22b** (THNAN69), achieved maximum degradation approximately 60 h after PROTAC treatment, reaching complete target degradation at least in this sensor cell line model ectopically expressing LIMK2. SAR analysis based on the developed sensor cell line confirmed the preference of LIMK2 degradation by the CRBN system. However, PROTAC **23c** demonstrated moderate LIMK2 degradation in the VHL system, suggesting that LIMK2 degraders may also be developed using VHL ligands. Nevertheless, **23c** performed poorly in biophysical assays (see Figure 2A) and showed only modest degradation in Western blots. Interestingly, isoform selectivity was also preserved for this VHL-based PROTAC (see Figure 3C).

### THNAN69 (22b) is a Potent LIMK2 Degrader

Consistent with findings from the EGFP–LIMK2 reporter cell line, Western blot analysis of HuCCT1 cells across an extended concentration range demonstrated near-complete depletion of endogenous LIMK2 at 100 nM for **21b** and 10 nM for THNAN69, respectively (Figure 4A). The DC_50_ values determined from the live-cell EGFP–LIMK2 assay for **21b**, **22c**, and THNAN69 were 33 nM, 13.7 nM, and 1 nM, respectively (Figure 4B and Figure S4A,B). Notably, replacing thalidomide in **21b** with the PD-based ligand enhanced the potency of **22c** and THNAN69 by 2.4-fold and 33-fold, respectively. THNAN69 differs from **22c** by only a single CH_2_ unit in the aliphatic linker. Remarkably, this subtle modification enhanced the potency of THNAN69 14-fold.

**Figure 4:**
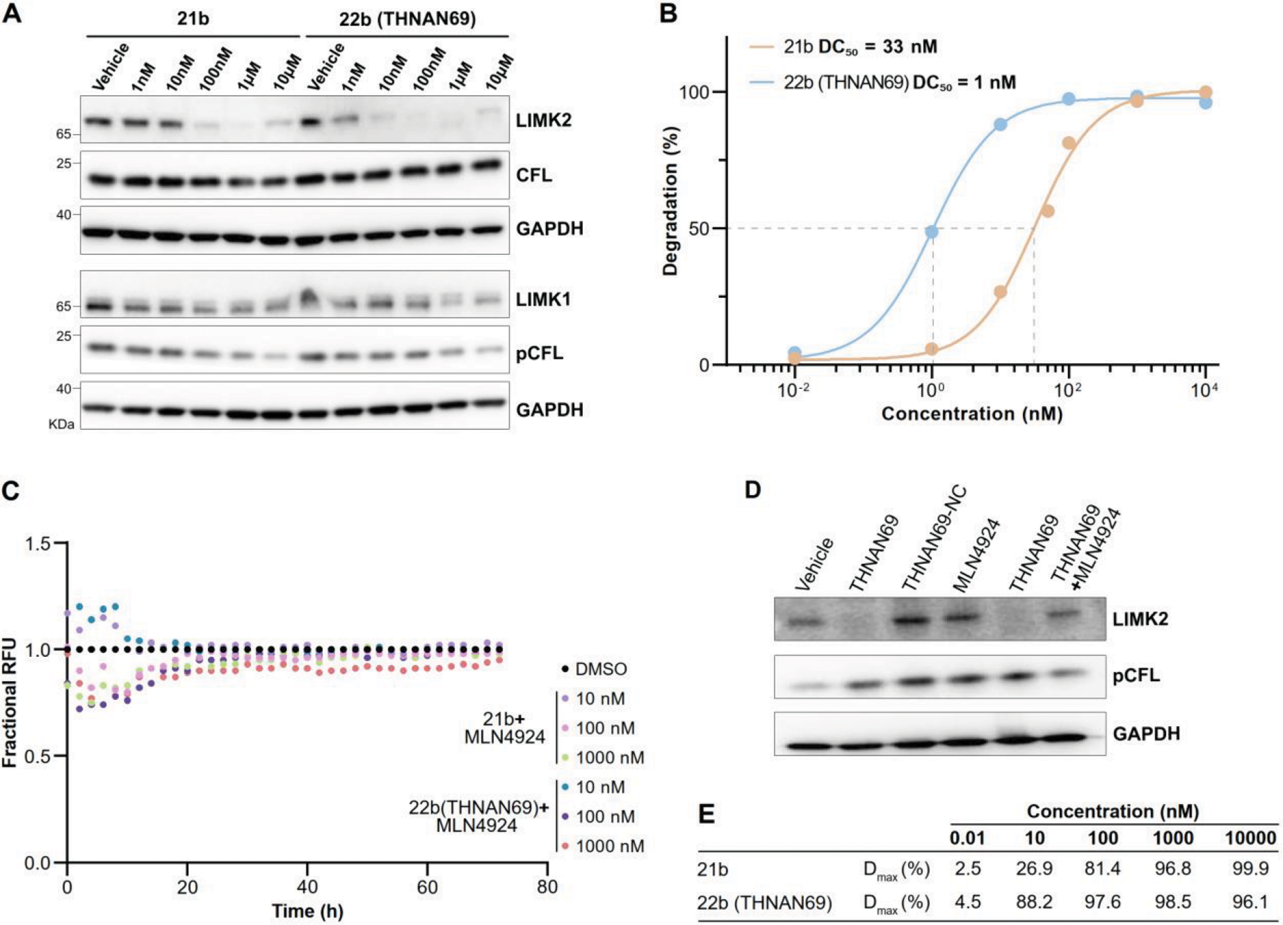
Cellular Characterization of 21b and THNAN69.(A) Western blot analysis of HuCCT1 cells following 6 h PROTAC treatment. LIMK isoforms, CFL, and pCFL levels were monitored, with GAPDH as a loading control. (B) Quantification of LIMK2 degradation potency. Mean maximum degradation (*D*_max_) and DC_50_ values were determined from the EGFP–LIMK2 kinetic assay data, as described by Riching et al.^35^ Data were fitted using a variable slope dose–response model. (C) Rescue of LIMK2 degradation by neddylation inhibition. CRBN-mediated cullin-RING ligase activity was assessed using the cellular EGFP–LIMK2 assay after cotreatment with three concentrations of 21b or THNAN69 and MLN4924 (1 μM). Fluorescence traces monitored remained stable over 72 h. (D) Validation of THNAN69-mediated endogenous LIMK2 degradation by Western blot (6 h). HuCCT1 cells were treated with THNAN69 (100 nM) ± MLN4924 (1 μM). THNAN69-NC (1 μM) was also included. LIMK2, pCFL, and GAPDH levels were monitored. (E) Summary of mean *D*_max_ values across varying PROTAC concentrations measured in the EGFP–LIMK2 cell-based assay.

To verify that LIMK2 degradation depends on cullin-RING ligase (CRL) activity via CRBN, we employed MLN4924 (Pevonedistat), a selective inhibitor of the NEDD8-activating enzyme. Using the developed EGFP–LIMK2 sensor cell line, we observed no LIMK2 degradation at three different PROTAC concentrations (10, 100, and 1000 nM) for both **21b** and THNAN69, supporting a CRL-dependent degradation mechanism via CRBN (Figure 4C). Moreover, the LIMKi3 warhead control alone did not induce any LIMK2 downregulation (Figure S4C). We used Western blotting to validate our proposed degradation mechanism for endogenous LIMK2. As an additional control, we included the negative control, THNAN69-NC, which was inactivated by methylation of the imide nitrogen in the PD ligand to prevent CRBN binding (see Schemes S2 and S3). As expected, MLN4924 rescued LIMK2 from THNAN69-mediated degradation, while THNAN69-NC did not induce any LIMK2 degradation (Figure 4D andFigure S4D). We observed no significant change in cofilin phosphorylation, indicating that, at the concentrations used, the kinase inhibitor moiety in THNAN69 did not cause enzymatic inhibition of LIM kinases. Moreover, degradation of LIMK2 alone was not sufficient to reduce cofilin phosphorylation levels, likely due to compensatory phosphorylation by LIMK1. Finally, we calculated maximum degradation levels (*D*_max_) from the EGFP–LIMK2 reporter data, showing that at 10 nM and 100 nM, LIMK2 protein levels were depleted by 88.2% and 97.6%, respectively, which correlates with the Western blot results (Figure 4E).

Both THNAN69 and its negative control showed no cytotoxic effects after 24-h treatment, as determined by the CellTiter-Glo cell viability assay (Figure S4E). We then evaluated metabolic stability in a physiologically relevant setting using rat liver microsomes. The PROTAC demonstrated high metabolic stability, with 85% of the parent PROTAC remaining after 60 min, as measured by High-Performance Liquid Chromatography (HPLC), further supporting our choice to incorporate click-chemistry-enabled alkyl-triazole linkers (Figure S4F).

### THNAN69 is Highly Selective in Proteome-Wide Screens

Since thalidomide-based degrader **21b** significantly degraded neo-substrates and Aurora kinase A (see Figure 3B), we next evaluated whether using the PD-CRBN ligand improves the selectivity of our developed LIMK2 degrader. Therefore, we evaluated THNAN69 using a proteome-wide screen after treating HuCCT1 cells for 6 h with 500 nM PROTAC. Encouragingly, we observed that LIMK2 was the only kinase that was selectively degraded. However, significantly lower protein levels were also detected for Cytochrome *b*-245 alpha chain (CYBA) and Deoxyguanosine kinase (DGUOK) (Figure 5A). As a control experiment, we performed a whole-cell proteomics analysis using LIMKi3 kinase inhibitor treatment alone under the same conditions. We observed similar fold changes and statistical significance for CYBA and DGUOK (Figure 5B). Thus, the observed downregulation of DGUOK and CYBA was independent of the ubiquitin system and related to the kinase inhibitor used in the PROTAC. Additionally, neither DGUOK nor CYBA was detected as downregulated when samples were analyzed 24 h after PROTAC treatment, further suggesting that the effect was transient (Figure S5A).

**Figure 5:**
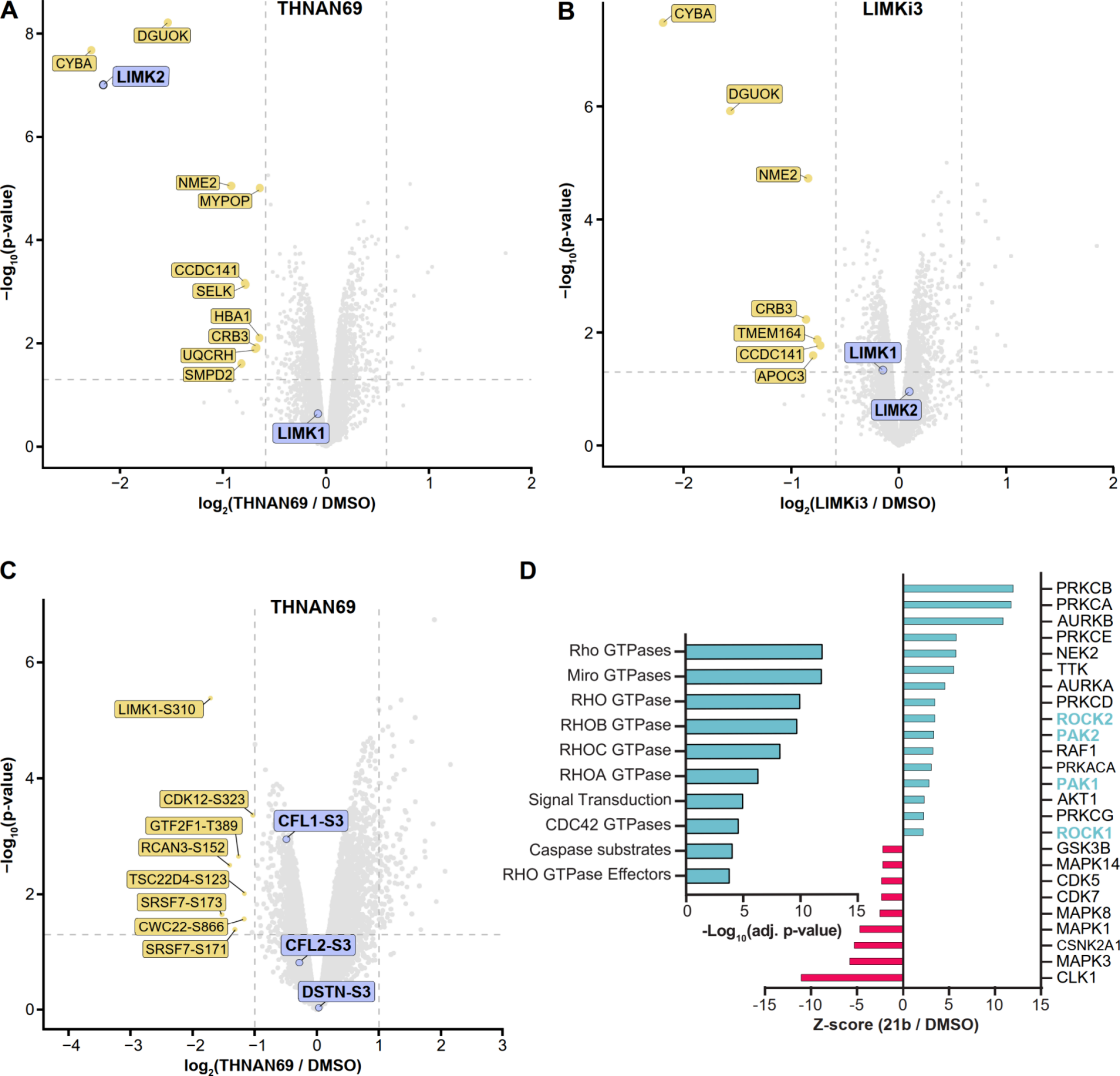
Selectivity of THNAN69 and Effects of LIMK2 Depletion on Phosphorylation Signaling. (A,B) Quantitative global proteomics of THNAN69 and LIMKi3 treatments in HuCCT1 cells (6 h). (A) Volcano plot of differential protein levels after THNAN69 treatment, revealed isoform-specific degradation of LIMK2. LIMKs are highlighted in purple, and other significantly downregulated proteins are shown in yellow. (B) Volcano plot of a proteomics experiment following LIMKi3 treatment under the same conditions. (C) Phospho-proteomics analysis of THNAN69 in HuCCT1 cells (6 h). Volcano plot revealed that LIMK phospho-Ser 3 (S3) substrates CFL1/2 and DSTN were not significantly downregulated (purple labels). (D) PROTAC treatment upregulated small G-protein-activated signaling pathways. Left panel: Global gene ontology analysis revealed upregulation of several Rho signaling pathways associated with PAK-ROCK-LIMK signaling. Right panel: KSEA Z-score analysis indicated increased phosphorylation and activation of kinases, including LIMK2-activating kinases ROCK1/2 and PAK1/2 (cyan). Kinases with decreased Z-scores are highlighted in red.

We hypothesized that the transient downregulation of DGUOK and CYBA was triggered by mitochondrial events. DGUOK is a mitochondrial kinase that phosphorylates deoxyribonucleosides and their analogs, thereby contributing to the maintenance of the mitochondrial DNA pool.^36,37^ Unphosphorylated serine 3 (Ser 3) CFL has been documented to associate with unphosphorylated serine 637 (Ser 637) dynamin-related protein 1 (Drp1) at the outer mitochondrial membrane, triggering mitochondrial fission.^38–40^ Initially, both LIMKi3 warhead-mediated inhibition of LIMKs and proteasome-mediated degradation of LIMK2 can increase the pool of unphosphorylated cofilin transiently, thereby enhancing mitochondrial fission, but the unaffected LIMK1 isoform likely compensates for LIMK2 degradation, facilitating the recovery of CFL phosphorylation. Moreover, dual LIMK inhibitors fail to completely eliminate CFL phosphorylation, suggesting that additional kinases may also target the CFL phosphorylation site. It is therefore possible that the downregulation of DGUOK and CYBA is an initial, transient byproduct of these events, with recovery likely driven by LIMK1 phosphorylation of CFL. We outlined a potential mechanism of this response in Figure S5B. Furthermore, when we investigated the selectivity of our parent PROTAC **21b** and LIMKi3 in the acute lymphoblastic leukemia (ALL) cell line MOLT-4, neither induced downregulation of CYBA and DGUOK, further suggesting that these two transient off-targets were specific to the HuCCT1 cell line (see Figures S3A and S5C).

### Selective Degradation of LIMK2 Did not Result in a Reduction of the pCFL Pool

CFL is a well-documented downstream effector in the LIMK-mediated signaling pathway. It is therefore expected that any significant depletion of LIMK2 via the PROTAC would affect CFL activity. Our initial Western blot analysis also monitored pCFL (see Figure 4A), but no significant changes in pCFL were observed, despite the potent degradation of LIMK2 by **21b** and THNAN69.

To analyze the effect of LIMK2 degradation on the phospho-proteome, we used proteomics to monitor changes in global phosphorylation sites. Both LIMK1 and LIMK2 have been reported to phosphorylate cofilin isoforms CFL1 and CFL2, as well as the actin-depolymerizing factor DSTN. Consistent with our Western blot data, no significant change in phosphorylation of these substrates was observed after treatment with 500 nM THNAN69 (Figure 5C). We hypothesized that LIMK1 could compensate by phosphorylating these well-documented substrates. At the same time, gene ontology analysis of upregulated phosphorylation events revealed upregulation of several Rho signaling pathways following PROTAC treatment (Figure 5D). Additionally, Kinase-Substrate Enrichment Analysis (KSEA) prediction analysis^41^ revealed elevated activity of LIMK1/2-activating kinases, PAK1/2, and ROCK1/2, along with several other kinases (Figure 5D), suggesting potential compensatory activation of small G-protein signaling pathways following PROTAC-mediated LIMK2 depletion.

### Rationalizing LIMK2 Isoform-Specific Degradation

It has been shown that both LIMK isoforms, which are highly conserved in domain architecture and structural fold (Figure 6A,B), are highly degradable kinases.^27^ These observations raise a key question regarding the selective degradation of LIMK2, but not LIMK1, by our PROTAC series. We also confirmed that isoform specificity was conserved in two diverse cell lines using two different assay systems.

**Figure 6:**
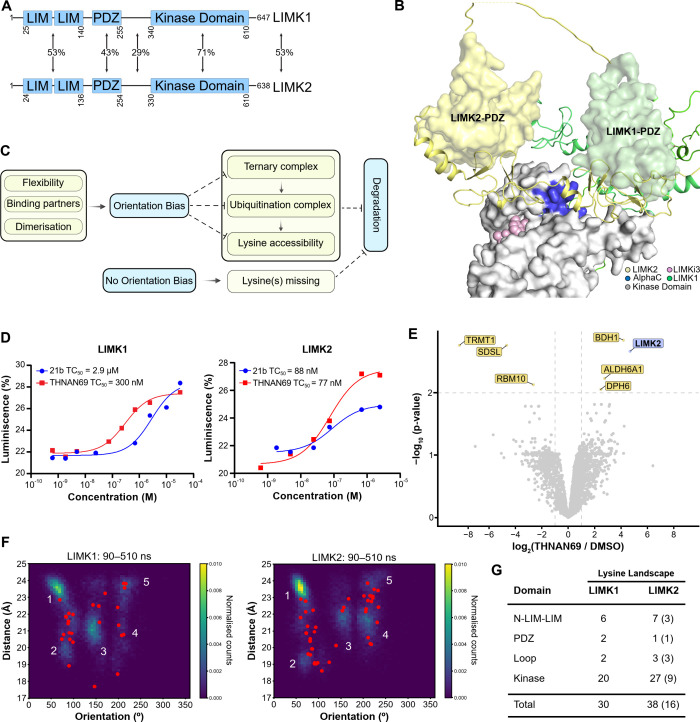
Reasons for Isoform Specificity. (A) Comparison of LIMK1 and LIMK2 at the domain and sequence level. Sequence conservation of full-length LIMKs, their individual domains, and the bridging loop in a pairwise alignment. (B) Crystal structure of LIMK1 bound to LIMKi3 (PDB: 8AAU), aligned with AlphaFold models of full-length LIMK1 and LIMK2 using PyMOL.^46^ (C) Mechanisms of isoform-specificity. Proposed schematic model depicting how potential variations in flexibility, interaction landscapes, and dimerization influence structural orientations and degradation outcomes. (D) LIMK1/2 cellular ternary complex formation. NanoBRET signals were measured at various PROTAC concentrations. The mean data were fitted to a variable-slope dose–response model to determine TC_50_ values. (E) CRBN:THNAN69 pull-down proteomics. Volcano plot of proteins identified by ProxiCapture pull-down from MOLT-4 cell lysate. Isoform-specific enrichment of LIMK2 (purple), but not LIMK1, was observed. Proteins significantly enriched or depleted relative to the control are highlighted in yellow. (F) MD simulations. LIMK1 kinase domain and LIMK2 kinase domain orientations relative to CRBN in the ternary complex, represented by the distance of the PROTAC warheads and their relative orientations. Initial starting structures are shown with red dots. Populated clusters 1–5 are highlighted. (G) Lysine landscape. The lysine landscape for each aligned domain in the 3D structural alignment is presented, with lysine in different orientations or positions noted in parentheses.

To understand isoform specificity, we present a schematic model outlining the potential sources of orientation bias and the hierarchical degradation-selectivity levels that can be affected, which in turn may induce degradation bias among closely related isoforms (Figure 6C). Conformational bias may stem from differences in structural flexibility, interaction partners, and dimerization events between the two LIMKs. These factors ultimately influence any of the three key layers of degradation selectivity: ternary complex formation, cullin-RING ligase (CRL) ubiquitination assembly, and lysine residue accessibility. Differences in conformational flexibility between the LIMK isoforms may originate from the flexible bridging-loop connecting the N-terminal LIM-LIM-PDZ domains to the kinase domain, which is poorly conserved (29% sequence identity), potentially contributing to differential conformational dynamics. To investigate how this flexibility can influence isoform-specific degradation, we aligned the full-length LIMKs with the LIMK1 kinase domain bound to LIMKi3 (PDB 8AAU). In our model, the LIMK2 PDZ domain was positioned about 20 Å above and in front of the kinase N-lobe, while the two LIM domains spanned the front-right face of the N-lobe. In contrast, for LIMK1, the PDZ domain was positioned about 15 Å above the activation loop with the two LIM domains extending diagonally up and across the back of the kinase N-lobe (see Figure 6B). The dynamic nature of the flexible-bridging loop allows LIMKs to adopt multiple conformations, and the AlphaFold structures reflected this by showing different average conformations for both the LIMKs, likely stemming from low confidence scores for the bridging loop. Additionally, differences in the interaction profiles and potential dimerization events between LIMK1 and LIMK2 were considered. For example, studies in the literature^42^ and our own mass photometry data (Figure S6A) showed that LIMK1 was capable of forming dimers, whereas no dimerization of LIMK2 has been reported so far.

Next, we assessed ternary complex formation for the parent and lead PROTACs in live cells using the NanoBRET proximity assay. A ternary complex assay system was established using NanoLuc-tagged LIMKs and Halo-tagged CRBN.^43^ Formation of the ternary complex upon PROTAC addition was quantified by measuring the BRET signal. Interestingly, we observed that THNAN69 induced ternary complexes for both LIMK1 and LIMK2. However, they differed in the strength of the ternary complex. LIMK2 formed a strong ternary complex with a ternary complex concentration 50 (TC_50_) of 77 nM, while LIMK1 formed one with a TC_50_ of 300 nM, which was 3.9-fold weaker than the LIMK2 complex (Figure 6D). Interestingly, THNAN69:LIMK2 also showed a similar binary complex IC_50_ window with an IC_50_ of 80 nM, which was 5-fold stronger than that of LIMK1 (see Figure 2A). We also confirmed the differences in ternary complex strength using the orthogonal PROXICapture approach,^44^ in which MOLT-4 cell lysate was passed over immobilized FLAG-tagged CRBN preincubated with THNAN69. Proteomic analysis of the elute revealed an interactome that selectively recruited and enriched LIMK2 but not LIMK1, suggesting that LIMK2 formed a stronger ternary complex compared to LIMK1 under the given selection conditions (Figure 6E).

In parallel, to further explore ternary complex structures and dynamics, we modeled 30 and 50 ternary complexes of the kinase domain, THNAN69, and CRBN for LIMK1 and LIMK2, respectively. These served as starting structures for a cumulative 40.8 μs of all-atom MD simulations. Distinct LIMK1 and LIMK2 orientations and distances to CRBN emerged after 90 ns of simulation time for each complex. After 500 ns, LIMK1 populated states 1, 2, 3, and 5, whereas LIMK2 additionally populated state 4 and reduced population in states 3 and 5 (Figure 6F and Figure S6B–E). Overall, the simulations suggested that LIMK2 adopted more distinct ternary complex orientations, whereas LIMK1 exhibited greater dynamics and potentially less stable ternary orientations, which may influence its degradability. To evaluate the ubiquitination potential of the two isoforms, we aligned representative structures from each simulated state to a static Cullin-4A E3 ligase complex system.^45^ Our analysis revealed that state 2 did not present any LIMK1/2 lysines in proximity to the terminal glycine of ubiquitin in the static ligase complex, whereas state 1 and state 3 provided a single lysine for potential ubiquitination. Notably, states 4 and 5 presented the most lysines in close proximity for potential ubiquitination. Taken together, differences in the accessible lysine profiles between states 4 and 5, along with stable and favorable ternary complex orientation states, may synergistically drive selective LIMK2 degradation (Figure S6F,G).

Finally, we mapped the lysine landscapes of both LIMK1 and LIMK2 across all domains, including the flexible bridging loop. Comparative domain alignments revealed differently positioned or absent lysine residues (Figure 6G and Figure S6H,I). LIMK2 contains eight additional lysine residues relative to LIMK1, seven of which are located within the kinase domain. Collectively, isoform-specific degradation of LIMK2, but not LIMK1, is likely driven by flexibility-induced orientation bias and/or enhanced lysine accessibility, coupled with stronger, stable, and favorable ternary complex formation. Ternary complex stability was also the major reason for investigating the efficacy of three different WDR5 PROTAC chemical series, which further highlights this property as an important predictor of PROTAC degradation efficacy.

## Discussion

Targeted degradation is increasingly being recognized for its therapeutic potential for the development of new therapies, as exemplified by the success of Bruton’s tyrosine kinase (BTK) degrader NX-2127.^47^ Despite growing interest in LIMKs as therapeutic targets and initial reports suggesting that they can be easily degraded, no potent and well-characterized degraders targeting LIM kinases have been reported to date. In this study, we developed a selective isoform-specific LIMK2 degrader showed excellent degradation potency (DC_50_), selectivity as well as high maximal depletion levels (*D*_max_). Comprehensive characterization of the LIMK2 selective THNAN69 confirmed proteasome-dependent degradation, favorable metabolic stability, and a lack of cytotoxicity. In addition, we developed a matching inactive negative control to complete the probe set targeting LIMK2. The physicochemical properties of THNAN69 fall outside conventional Lipinski roles, in particular regarding their molecular weight. Despite these properties, THNAN69 demonstrated excellent cellular activity and cell penetration, consistent with the ability of PROTACs to achieve effective target engagement also through cooperative ternary complex formation.^48^ Importantly, THNAN69 meets all key criteria for a high-quality chemical degrader probe for the evaluation of the cellular roles of LIMK2.^49^

In our study, we demonstrated that recent development of CRBN ligands can significantly improve PROTAC efficacy and avoid neosubstrate degradation. Additionally, optimization of the linker length proved critical for fine-tuning the degrader’s overall performance. In particular, removal of a single carbon likely reduced excessive flexibility, favoring a more constrained and productive ternary complex that facilitates subsequent ubiquitination. However, we did not extensively explore the chemical space during our optimization process. To facilitate such studies, our laboratory has established a ″direct-to-biology″ platform based on click chemistry, which should significantly accelerate SAR studies in the future.^50^ This approach may offer a promising strategy for expanding the degrader toolbox toward LIMK1-specific as well as pan LIMK degradation, which has also been identified as a “highly degradable” target.^27^ Rational design approach, combined with structural tuning of ligands and linkers, and high-throughput assay screening technology,^32,51^ highlights a powerful strategy for improving PROTAC selectivity and efficacy across diverse kinase targets.

Our findings from ternary complex assays, structural modeling, and MD simulations highlight the role of ternary complex formation, conformational flexibility, and lysine accessibility in contributing to isoform specificity. These observations highlight selective ternary and CRL complex assembly as a promising strategy for isoform-specific targeting, where conventional inhibitors fail. Future studies could explore how these determinants can be further leveraged to enhance selectivity across kinase subfamilies.

We anticipated THNAN69 to have an impact on pCLF levels due to its high potency (DC_50_ = 1 nM) and its ability to eliminate noncatalytic functions. However, previous studies in acute myeloid leukemia (AML)^6,52^ using LIMK2 knockdown studies revealed LIMK1 upregulation, suggesting a compensatory relationship. We also observed compensatory effects in the RHO-PAK-ROCK-LIMK pathway, which suggests that cells maintain homeostasis in the actin cytoskeleton. These findings have significant implications for the development of drugs targeting this pathway. Compensatory effects resulted also in our observation that the phosphorylation levels of the central target pCFL seemed to remain constant. In line with these findings, LIMK1/2 double knockout mice showed reduced pCFL levels, in contrast to individual knockouts of either LIMK1 or LIMK2.^53^ Supporting this, dependency data for LIMK1/2 from the DepMap portal^54^ (Public 24Q1 data set) revealed that individual silencing or knockout of either LIMK1 or LIMK2 did not impair the viability of several hundred cancer cell lines, indicating that neither isoform may be essential for cancer cell survival when modulated alone. These findings suggest that effective targeting of LIMK2-dependent pathways may require coinhibition of compensatory mechanisms to achieve sustained cytoskeletal disruption, for instance, by employing the existing array of LIMK/PAK inhibitors in combination with PROTAC and assessing tissue and context-specific dependencies on LIMK2 versus LIMK1.

## Conclusion

Despite advances in kinase inhibitor development and rational targeting strategies, isoform specificity often remains elusive. Here, we report on the development and comprehensive characterization of an isoform-specific LIMK2 degrader. This work also establishes the successful use of the phenyl dihydrouracil CRBN ligand and subtle linker optimization to enhance degradation potency and minimize neo-substrate degradation associated with thalidomide based CRBN ligands. We found that LIMK2 degradation stimulated compensatory Rho signaling, emphasizing the cellular importance of maintaining phospho-cofilin levels that are also maintained by the closely related LIMK1, which was not degraded. Structural comparison and molecular dynamics (MD) simulation revealed that isoform specificity likely stemmed from enhanced ternary complex formation, coupled with a more stable and favorable orientation for LIMK2 degradation associated with lysine accessibility. More broadly, our findings highlight how PROTACs can exploit structural nuances to achieve isoform-selectivity beyond the reach of conventional inhibitors. Finally, the developed PROTAC, THNAN69 serves as a valuable chemical probe for dissecting LIMK2 isoform-dependent biology.

## Methods

### Cell Lines

HEK293T cells (human embryonic kidney; sex: female; ATCC, CRL-3216).

MOLT-4 cells (human acute lymphoblastic leukemia; sex: male; ATCC, CRL-1582).

HuCCT1 cells (human intrahepatic cholangiocarcinoma; sex: male; ATCC, CRL-1997).

HEK293T cells were cultured in DMEM, while MOLT-4 and HuCCT1 were cultured in RPMI-1640 medium. Media was supplemented with 10% fetal bovine serum (FBS) and 1% penicillin-streptomycin at 37 °C in a humidified incubator with 5% CO_2_. Cells were routinely tested for mycoplasma contamination using the MycoAlert Mycoplasma Detection Kit (Lonza), and authentication was performed by ATCC using short tandem repeat (STR) profiling.

### DSF Assay

A 2 μM LIMK1 solution in 20 μL assay buffer (20 mM HEPES, pH 7.4, 150 mM NaCl, 0.5 mM TCEP, 5% glycerol) was mixed 1:1000 with SYPRO Orange dye (Sigma) and compounds (10 μM final concentration). Fluorescence was monitored from 25–95 °C using an Mx3005P PCR instrument (excitation/emission: 465/590 nm). Data were analyzed with MxPro software.

### Recombinant LIMK1 Expression and Purification

Human LIMK1­(330–S637), LIMK1-avi­(330–S637) (C-terminally Avi-tagged), and full-length LIMK1 expression constructs, tagged with an N-terminal TEV-cleavable 6 × His-Z-tag, were cloned into the pFB-6HZB vector and expressed in insect cells following baculoviral transfection. LIMK1-avi­(330–S637) was coexpressed from a dual pFB-6HZB vector encoding BirA, with biotin supplemented in the culture medium to enable in vivo biotinylation. Briefly, exponentially growing insect cells (2 × 10^6^ cells/mL, Novagen) cultured in serum-free Insect-Xpress Medium (Lonza) were infected with recombinant baculovirus stock and incubated for 66 h at 27 °C with shaking. Cells were harvested by centrifugation and resuspended in lysis buffer (50 mM HEPES, pH 7.4, 500 mM NaCl, 20 mM imidazole, 0.5 mM TCEP, 5% glycerol), followed by sonication.

After clarification by centrifugation, the lysate was loaded onto pre-equilibrated Ni-NTA Sepharose beads (Qiagen). After washing with lysis buffer, His6-tagged proteins were eluted with lysis buffer containing 300 mM imidazole. The NaCl concentration of the eluate was then reduced to 250 mM before loading onto an SP Sepharose column (Cytiva). Proteins were eluted using a salt gradient from 250 mM to 2.5 M NaCl.

Fractions containing LIMK1 were pooled, and the N-terminal tag was cleaved overnight by TEV protease. Contaminating proteins, cleaved tags, and TEV protease were removed by passage over reverse SP Sepharose and Ni-NTA columns. The purified LIMK1 protein was concentrated and further purified by size-exclusion chromatography using an AKTA pure system connected to a S200 16/600 gel filtration column (GE Healthcare), equilibrated in buffer containing 20 mM HEPES, pH 7.4, 150 mM NaCl, 0.5 mM TCEP, 5% glycerol. Pooled fractions were analyzed by SDS-PAGE to assess purity. Intact mass spectrometry was performed to confirm molecular weight and successful biotinylation.

### SPR Binding Assays

The SPR analysis was performed on a Biacore T200 (Cytiva Life Sciences) at 25 °C. Approximately 7000 RU of biotinylated LIMK1 was loaded onto a Series S CM5 chip coated with Streptavidin. All experiments were performed at 25 °C. The chip was equilibrated with a running buffer containing 20 mM HEPES, pH 7.4, 150 mM NaCl, 0.5 mM TCEP, and 0.05% Tween 20. A titration of serially diluted compounds was performed. The compounds were allowed to bind over the surface at a flow rate of 30 μL/min for 60 s, followed by a disassociation wash for 180 s. The sensorgrams were double-reference subtracted and analyzed using the Biacore evaluation software, and curves were fitted to a steady-state affinity fit model.

### VHL Binary and Ternary Complex Formation

Approximately 1000 RU of biotinylated VHL:ELOB:ELOC was loaded onto a Series S CM5 chip coated with Streptavidin. All experiments were performed at 25 °C. The chip was equilibrated with a running buffer containing 20 mM HEPES, pH 7.4, 150 mM NaCl, 0.5 mM TCEP, 1% BSA, and 0.05% Tween 20.

To assess ternary complex formation, 1 μM of VHL-binding PROTAC was preincubated with a 2-fold molar excess of LIMK1 to promote binary complex formation and minimize the hook effect. The resulting binary complex was injected over the immobilized VHL:ELOB:ELOC surface at a flow rate of 30 μL/min for 60 s, followed by a dissociation phase of 180 s. An increase in resonance signal relative to the binary complex alone was used as an indicator of ternary complex formation. In separate experiments, binary and ternary complex affinities were measured and compared using serial dilutions as described for LIMK1. Additionally, the association rate constant (*k*_a_), dissociation rate constant (*k*_d_) were determined for the binary complexes.

### NanoBRET Assays

Gene encoding LIMK1 (Promega, NV3391) and LIMK2 (Promega, NV1531), CRBN (Promega, N2741) proteins cloned in frame with NanoLuc fusion tag at the C-terminus, were transfected into HEK293T cells using FuGENE HD (Promega, E2312) following manufacture’s protocol and proteins were allowed to express for 20 h at 37 °C and 5% CO_2_. Additionally, for CRBN nanoBRET, Human DNA damage-binding protein (DDB1) expression vector (Promega, N2761) was cotransfected along with the E3 ligase vector to improve the overall assay quality. The 10 μL of transfected cells after trypsinization and resuspending in Opti-MEM (Life Technologies, 31985070) were dispensed into each well of the 384-well plate (Greiner 781207) at a cell density of 2 × 10^5^ cells/mL. For dose–response BRET measurements, compounds at various concentrations, immediately followed by Tracer K10 (Promega, Tracer DB ID: T000008) for LIMK1 and LIMK2, CRBN Tracer (Promega, Tracer DB ID: T000018) for CRBN at an optimum KD concentration chosen from TracerDB (tracerdb.org), were pipetted using an Echo acoustic dispenser (Labcyte). The system was allowed to equilibrate for 2 h at 37 °C and 5% CO_2_ before BRET measurements. To measure BRET signal, the NanoBRET NanoGlo Substrate (Promega, N1573) was added as per the manufacturer’s protocol, and filtered luminescence was measured on a PHERAstar plate reader (BMG Labtech) equipped with a luminescence filter pair (450 nm BP filter (donor) and 610 nm LP filter (acceptor)). For lysed NanoBRET measurement, 25 nL of Digitonin (0.05 μg/μL) was pipetted to each well using Labcyte, and the plate was incubated for 5 min at 37 °C and 5% CO_2_. After incubation, the BRET signals were measured by a PHERAstar plate reader following the same procedure as intact NanoBRET measurement. In our assay we have 2 controls for normalization, 100% control - DMSO treated + Tracer, 0% control - DMSO treated without tracer. The BRET ratios are normalized to both of them in the prism output data file. Competitive displacement data were then normalized to controls and were graphed using GraphPad Prism 9 software employing a normalized 3-parameter curve fit with the following equation: Y = 100/(1 + 10̂(X – logIC_50_)).

### NanoBRET LIMK1/2 Ternary Complex Formation

Gene encoding LIMK1 (Promega, NV3391) and LIMK2 (Promega, NV1531) proteins cloned in frame with NanoLuc fusion tag at the C-Terminus and gene encoding cereblon (generated in-house) cloned in frame with Halo Tag at the N-Terminus, were used to measure the end-point ternary complex formation between the PROTACs and LIMK1 and LIMK2, respectively. The essential plasmids were transfected into HEK293T cells using FuGENE HD (Promega, E2312) following the manufacturer’s protocol, and proteins were allowed to express for 20 h at 37 °C and 5% CO_2_ in a T25 flask. After trypsinization and resuspension in Opti-MEM Reduced Serum Medium (Life Technologies, 31985070), 10 μL of the transfected cells were pipetted into each well of a 384-well plate (Greiner, 781207) at a cell density of 2.5 × 10^5^ cells/mL. The cells were allowed to equilibrate for 1 h at 37 °C and 5% CO_2_ and 10 nL of HaloTag NanoBRET 618 Ligand (PROMEGA, G9801) was added to the cells using an Echo acoustic dispenser (Labcyte) and the cells were incubated for additional 20 h at 37 °C and 5% CO_2_. Four hours before the BRET measurement, 750 nM of MG132 was added to all the measurement wells, using an Echo acoustic dispenser (Labcyte) and the cells were incubated for 1 h at 37 °C and 5% CO_2_. Following 1-h incubation with MG132, the compounds were pipetted onto the cells using an Echo acoustic dispenser (Labcyte), and the cells were incubated for a further 3 h at 37 °C and 5% CO_2_ to allow ternary complex formation. To measure BRET, NanoBRET NanoGlo Substrate + Extracellular NanoLuc Inhibitor (Promega, N2162) was added to cells as per the manufacturer’s protocol, and filtered luminescence was measured on a PHERAstar FSX plate reader (BMG Labtech) equipped with a luminescence filter pair (450 nm BP filter (donor) and 610 nm LP filter (acceptor)). Stimulation data were then graphed using GraphPad Prism 9 software using a normalized 3-parameter curve fit with the following equation: Y = Bottom + (Top-Bottom)/(1 + 10̂((LogEC_50_-X))). Donor-contributed background or bleedthrough was corrected for by subtracting BRET ratios from no HaloTag NanoBRET 618 Ligand control cells from treated cells.

### Cell Line Treatment and Immunoblotting

HuCCT1 cell line was cultured in Roswell Park Memorial Institute (RPMI) 1640 medium supplemented with GlutaMAX, 10% fetal bovine serum (FBS), and 1% penicillin/streptomycin. The cell line was maintained in an incubator with 5% CO_2_ at 37 °C. For the treatment, 3 × 10^5^ cells were plated and treated with the compounds and DMSO as the control for 6 h. The cells were then lysed using RIPA buffer (50 mM Tris-HCl pH 7.4, 150 mM NaCl, 1% Triton X-100, 1% NaDOC, 0.1% SDS, 1 mM EDTA), freshly supplemented with protease and phosphatase inhibitor cocktails (Sigma-Aldrich, Darmstadt, Germany). After 1 h incubation on ice, the lysates were centrifuged for 30 min at 250 × g, and the protein-containing supernatant was collected. The protein concentrations were determined using Protein Assay Dye Reagent Concentrate (Bradford, Bio-Rad, Hercules, CA, USA) and Pre-Diluted Protein Assay Standards: Bovine Serum Albumin (BSA) Set (Thermo Fisher Scientific, Darmstadt, Germany). Then, equal amounts of protein was mixed with Roti-Load 1 dye (Carl Roth, Karlsruhe, Germany) and 4x Laemmli Buffer (Bio-Rad, Hercules, CA, USA), denatured at 95 °C, and run on NuPAGE 4–12% bis-Tris gels (Thermo Fisher Scientific). Proteins were blotted on methanol-activated PVDF Transfer Membranes (Thermo Fisher Scientific) using the wet transfer method with the transfer buffer (Thermo Fisher Scientific) and 20% methanol. Afterward, the membranes were blocked with 5% milk or 3% BSA in 0.1% TBS-T for 1 h at RT and incubated overnight at 4 °C with primary antibodies: GAPDH (catalog no. 2118, Cell Signaling Technology, MA, USA, 1:1000 dilution), LIMK1 (catalog no. 3842, Cell Signaling Technology, 1:1000 dilution), LIMK2 (catalog no. 3845, 1:500 dilution), phospho-Cofilin (catalog no. 3313, Cell Signaling Technology, 1:1000 dilution), Cofilin (catalog no. 5175, Cell Signaling Technology, 1:1000 dilution). Then, the membranes were washed with 0.1% TBS-T and incubated with secondary horseradish-peroxidase (HRP)-conjugated antibody for 2 h (catalog no. 7074, Cell Signaling Technology, 1:3000 dilution). The membranes were washed again with 0.1% TBS-T before they were developed using X-ray films (Fujifilm, Dusseldorf, Germany).

### EGFP-LIMK2 Depletion Assay

LIMK2 was cloned into pcDNA5-EGFP-NLS-P2A-mCherry-PTS1 (Addgene plasmid #87803) by replacing the NLS sequence with the LIMK2 sequence. pcDNA5-EGFP-NLS-P2A-mCherry-PTS1 was a gift from Andrea Musacchio. Subsequently, EGFP-YTHDF2-P2A-mCherry was cloned into the lentiviral vector pCW57-MCS1-P2A-MCS2 (Hygro) (Addgene plasmid #80922) to generate pCW57-HygB-EGFP-LIMK2-P2A-mCherry. pCW57-MCS1-P2A-MCS2 (Hygro) was a gift from Adam Karpf.

HEK293T cells (ATCC, CRL-3216) were cultured in Dulbecco’s Modified Eagle’s Medium (DMEM), while HUCCT1 cells (ATCC, CRL-1997) were cultured in RPMI medium supplemented with 10% fetal bovine serum (FBS) and 1% penicillin/streptomycin (P/S) antibiotic mix (all from Sigma-Aldrich, St. Louis, Missouri, USA). Lentiviral particles were produced in HEK293T cells using pCW57-HygB-EGFP-LIMK2-YTHDF2-P2A-mCherry, pMD2.G, and psPAX2. HUCCT1 cells were transduced with lentivirus to express LIMK2.

#### Generation of Stable Cell Lines

Briefly, the cells were plated and propagated in 6-well plates overnight. The next day, transduction with viruses was performed. Two days later, transduced cells with pCW57-HygB-EGFP-LIMK2-P2A-mCherry plasmids were selected using 100 μg/mL Hygromycin B. After at least three passages, the cells were sorted for the same populations of RFP- and GFP-expressing cells using the SH800S Cell Sorter (Sony Biotechnology, San Jose, CA, USA). Stable pools of cells were maintained in the same medium used for selection.

#### Incucyte Experiments

To assess the turnover of LIMK2, we used the Incucyte S3 or XS5 live-cell imaging system and performed the assay as previously described.^56,57^ Briefly, 2,000 HUCCT1 cells stably expressing EGFP-LIMK2-P2A-mCherry were seeded per well in a 384-well plate in 50 μL of RPMI medium supplemented with 10% fetal bovine serum (FBS) and 1% penicillin/streptomycin. Cells were incubated for 24 h before treatment. To induce LIMK2 expression, 1 μg/mL of doxycycline (Sigma-Aldrich, D9891–1G) was added at the time of seeding.

After 24 h, 50 μL of medium containing either 0.1% DMSO or the desired concentration of PROTACs was added. Fluorescence in three channels—GFP, RFP, and phase contrast (cell confluence)—was monitored every 2 h for 72 h using the Incucyte S3 or XS5 (Sartorius, Germany). LIMK2 turnover was quantified by calculating the ratio of total fluorescence intensity of RFP to GFP. Each time point represents the average ratio from three technical replicates. The assay was independently repeated at least three times (biological replicates) with consistent results.

### Cell Viability Assay

The effects of the PROTAC on cell viability were determined using the CellTiter-Glo 2.0 Cell Viability Assay (Promega, G9241) following the manufacturer’s protocol. 40 μL of HEK293T cells were seeded with 10000 cells/well into white 384-well plates (Greiner, 781207) and the cells were allowed to attach for 24 h at 37 °C and 5% CO_2_. After incubation, PROTACs were titrated using an Echo acoustic dispenser (Labcyte), and the plate was further incubated for 24 h at 37 °C with 5% CO_2_. 40 μL of assay reagent was added and incubated for 10 min at RT. Filtered luminescence was measured using a PHERAstar plate reader (BMG Labtech), and data were analyzed using GraphPad Prism 9.

### Microsomal Stability Assay

The solubilized test compound (5 μL, final concentration 10 μM) was preincubated at 37 °C in 432 μL of 0.1 M phosphate buffer (pH 7.4) together with 50 μL of an NADPH-regenerating system containing 30 mM glucose-6-phosphate, 4 U/mL glucose-6-phosphate dehydrogenase, 10 mM NADP, and 30 mM MgCl_2_. After 5 min, the reaction was initiated by adding 13 μL of rat liver microsomes (Sprague–Dawley, Invitrogen; 20 mg mL^–1^ protein in 0.1 M phosphate buffer) and incubated in a shaking water bath at 37 °C. The reaction was quenched at 0, 15, 30, and 60 min by adding 500 μL of ice-cold methanol. Samples were centrifuged at 5,000 × g for 5 min at 4 °C, and the supernatants were analyzed by HPLC to quantify the remaining parent compound. HPLC conditions were as follows: mobile phase composed of methanol (40–90%) and water with 0.1% formic acid (10–60%), optimized for the test compound; flow rate 1 mL/min; stationary phase Purospher STAR RP-18 column (5 μm, 125 × 4 mm) with a guard column (5 μm, 4 × 4 mm); detection wavelengths at 254 and 280 nm; injection volume 50 μL. Controls included samples without NADPH (to control for nonenzymatic degradation), with heat-inactivated microsomes (20 min at 90 °C), and without test compound (to determine baseline signal). Compound concentrations were determined using an external calibration curve. Data are expressed as mean ± SEM of the remaining parent compound from three independent experiments.

### Mass Photometry Analysis

Purified recombinant full-length LIMK1 was used for mass photometry experiments. All measurements were performed using a Refeyn TwoMP instrument. High-precision microscope cover glasses (Thorlabs, CG15KH) were cleaned and prepared with Grace Bio-Laboratories CultureWell gaskets (Sigma-Aldrich, GBL103350). For autofocus, 15 μL of dilution buffer (20 mM HEPES, pH 7.5, 150 mM NaCl, 0.5 mM TCEP) was added to each well, followed by in-drop dilution of LIMK1 to a final concentration of 50 nM. Videos were recorded for 1 min at RT, and calibration was performed using standard proteins: carbonic anhydrase (29 kDa), albumin (66 kDa), and β-amylase (56, 112, and 224 kDa). The ratiometric contrast data were analyzed using Refeyn DiscoverMP software, with molecular masses determined based on the experimentally derived calibration curve.

### Mass Spectrometry-Based Proteomics

Cells were lysed in SDS-lysis buffer (50 mM Tris, pH 8.5, 2% SDS, 10 mM TCEP, 40 mM chloroacetamide, protease inhibitor cocktail, phosphatase inhibitor) and boiled at 95 °C for 10 min. Proteins were precipitated using methanol-chloroform and resuspended in 8 M urea, 50 mM Tris, pH 8.5. Proteins were digested with 1:50 w/w LysC (Wako Chemicals, cleaves at the carboxylic side of lysine residue) and 1:100 w/w trypsin (Promega, Sequencing-grade) overnight at 37 °C after dilution to a final urea concentration of 1 M using 50 mM Tris pH 8.5. Digested peptides were then acidified (pH 2–3) using trifluoroacetic acid (TFA) and purified using C18 SepPak columns (Waters). Desalted peptides were dried and resuspended in TMT-labeling buffer (200 mM EPPS, pH 8.2, 20% acetonitrile). 10 μg of peptides per condition were subjected to TMT labeling with a 1:2.5 peptide TMT ratio (w/w) for 1 h at RT. The labeling reaction was quenched by the addition of hydroxylamine to a final concentration of 0.5% and incubation at RT for 15 min. Successful TMT labeling was verified by mixing equimolar ratios of peptides and subjecting the mix to single-shot liquid chromatography–tandem mass spectrometry (LC-MS) analysis. For high pH reversed phase fractionation on a Dionex analytical HPLC, 50 μg of pooled and purified TMT labeled samples were resuspended in 10 mM ammonium-bicarbonate (ABC), 5%ACN, and separated on a 250 mm long C18 column (Aeris Peptide XB-C18, 4.6 mm ID, 2.6 μm particle size; Phenomenex) using a multistep gradient from 100% Solvent A (5% ACN, 10 mM ABC in water) to 60% Solvent B (90% ACN, 10 mM ABC in water) over 70 min. Eluting peptides were collected every 45 s into a total of 96 fractions, which were cross-concatenated into 24 fractions and dried in a vacuum concentrator and resuspended in 3% ACN, 0.1% TFA for LC-MS analysis.

#### Mass Spectrometry Data Acquisition (Proteome of HuCCT1 Cells)

Tryptic peptides were analyzed on Orbitrap Ascend coupled to a VanquishNeo (ThermoFisher Scientific) using a 25 cm long, 75 μm ID fused-silica column packed in-house with 1.9 μm C18 particles (Reprosil pur, Dr. Maisch), and kept at 50 °C using an integrated column oven (Sonation). HPLC solvents consisted of 0.1% formic acid in water (Buffer A) and 0.1% formic acid, 80% acetonitrile in water (Buffer B). Assuming equal amounts in each fraction, 400 ng of peptides were eluted by a nonlinear gradient from 7 to 40% B over 90 min, followed by a stepwise increase to 90%B in 6 min, which was held for another 9 min. A synchronous precursor selection (SPS) multinotch MS3 method was used to minimize ratio compression as previously described.^58^ Full-scan MS spectra (350–1400 *m*/*z*) were acquired at a resolution of 120,000 at *m*/*z* 200, a maximum injection time of 50 ms, and an AGC target value of 4 × 10^5^. The most intense precursors with charge state between 2 and 6 were selected for fragmentation (“Top Speed” with a cycle time of 1.5 s) and isolated with a quadrupole isolation window of 0.7 Th. MS2 scans were performed in the Ion trap (Turbo) using a maximum injection time of 35 ms, AGC target value of 10000, and fragmented using CID with a normalized collision energy (NCE) of 35%. SPS-MS3 scans for quantification were triggered only after a successful Real-time search against the human canonical reference proteome from SwissProt. Criteria for passing the search were Xcorr: 1, dCn: 0.1, and precursor mass accuracy: 10 ppm. Maximum search time was 35 ms. MS3 acquisition was performed on the 10 most intense MS2 fragment ions with an isolation window of 0.7 Th (MS) and 2 *m*/*z* (MS2). Ions were fragmented using HCD with an NCE of 50% and analyzed in the Orbitrap with a resolution of 45,000 at *m*/*z* 200, scan range of 100–200 *m*/*z*, AGC target value of 150000, and a maximum injection time of 91 ms. Repeated sequencing of already acquired precursors was limited by setting a dynamic exclusion of 60 s and 7 ppm, and advanced peak determination was deactivated. All spectra were acquired in centroid mode.

#### Mass Spectrometry Data Acquisition (proteome of MOLT-4 Cells)

Tryptic peptides were analyzed on an Orbitrap Lumos coupled to an easy nLC 1200 (ThermoFisher Scientific) using a 35 cm long, 75 μm ID fused-silica column packed in-house with 1.9 μm C18 particles (Reprosil pur, Dr. Maisch), and kept at 50 °C using an integrated column oven (Sonation). HPLC solvents consisted of 0.1% formic acid in water (Buffer A) and 0.1% formic acid, 80% acetonitrile in water (Buffer B). Assuming equal amounts in each fraction, 400 ng of peptides were eluted by a nonlinear gradient from 7 to 40% B over 90 min, followed by a stepwise increase to 90%CB in 6 min, which was held for another 9 min. A synchronous precursor selection (SPS) multinotch MS3 method was used to minimize ratio compression as previously described. Full-scan MS spectra (350–1400 *m*/*z*) were acquired at a resolution of 120,000 at *m*/*z* 200, a maximum injection time of 100 ms, and an AGC target value of 4 × 10^5^. The most intense precursors with charge state between 2 and 6 were selected for fragmentation (“Top Speed” with a cycle time of 1.5 s) and isolated with a quadrupole isolation window of 0.7 Th. MS2 scans were performed in the Ion trap (Turbo) using a maximum injection time of 50 ms, AGC target value of 15000, and fragmented using CID with a normalized collision energy (NCE) of 35%. MS3 acquisition was performed on the 10 most intense MS2 fragment ions with an isolation window of 0.7 Th (MS) and 2 *m*/*z* (MS2). Ions were fragmented using HCD with an NCE of 50% and analyzed in the Orbitrap with a resolution of 50,000 at *m*/*z* 200, scan range of 100–500 *m*/*z*, AGC target value of 150000, and a maximum injection time of 86 ms. All spectra were acquired in centroid mode.

#### Mass Spectrometry Data Acquisition (Phospho-proteome of HuCCT1 cells)

Tryptic peptides were analyzed on an Orbitrap Lumos coupled to an easy nLC 1200 (ThermoFisher Scientific) using a 35 cm long, 75 μm ID fused-silica column packed in-house with 1.9 μm C18 particles (Reprosil pur, Dr. Maisch), and kept at 50 °C using an integrated column oven (Sonation). HPLC solvents consisted of 0.1% Formic acid in water (Buffer A) and 0.1% Formic acid, 80% acetonitrile in water (Buffer B). Peptides were eluted by a nonlinear gradient from 3% to 35% over 120 min, followed by a stepwise increase to 90% B in 6 min, which was held for another 9 min. Full scan MS spectra (350–1400 *m*/*z*) were acquired with a resolution of 120,000 at *m*/*z* 200, maximum injection time of 100 ms, and AGC target value of 4 × 10^5^. The most intense precursors with a charge state between 2 and 5 per full scan were selected for fragmentation (“Top Speed” with a cycle time of 1.5 s) and isolated with a quadrupole isolation window of 0.7 Th. MS2 scans for quantification were performed on the 10 most intense MS ions with an isolation window of 0.7 Th. Ions were fragmented using HCD with a normalized collision energy of 35% and analyzed in the Orbitrap with a resolution of 50,000 at *m*/*z* 200, scan range of 100–500 *m*/*z*, AGC target value of 1 × 10^5^, and a maximum injection time of 86 ms. All spectra were acquired in centroid mode.

#### Mass Spectrometry Data Analysis (Proteome/Phospho-proteome)

MS raw data were analyzed using MaxQuant (2.4.2.0).^59^ Acquired spectra were searched against a database containing 20.594 human protein sequences (Taxonomy ID 9606) downloaded from UniProt (released 02–2023) and a collection of common contaminants using the Andromeda search engine integrated in MaxQuant.^60^ Identifications were filtered to obtain false discovery rates (FDR) below 1% for both - peptide spectrum matches (PSM; minimum length of 7 amino acids) and proteins - using a target-decoy strategy.^61^ Spectra were searched with a mass tolerance of 6 ppm in MS mode, 20 ppm in HCD MS2 mode, strict trypsin specificity, and allowing up to 2 miscleavages. Carbamidomethylated cysteine was set as a fixed modification, and oxidation of methionine and N-terminal protein acetylation as variable modifications, allowing up to 5 modifications per peptide. Phosphorylation of serine, threonine, and tyrosine was set as a variable modification in the case of phospho-proteomics. The obtained data was further processed using the R Studio environment. Only proteins quantified in all replicates after standard filtering were used for statistical analysis. TMT channels were normalized using quantile normalization from the Limma package.^62^ Proteins were deemed significantly regulated using the moderated Limma *t* test. Phosphopeptides were additionally filtered for Localization probability >0.75, Delta score >0, and Score difference >0.

### PROXICapture Pull-Down Proteomics

Purified FLAG-CRBN was conjugated to FLAG beads (Chromotek, cat. no. ffak) in coimmunoprecipitation buffer (50 mM Tris pH-7.5, 120 mM NaCl, 1% NP40, 0.5 mM EDTA) for 1 h at 4 °C on a rotating shaker, before addition of DMSO or 5 μM THNAN69 for 30 min at 4 °C. Subsequently, ∼ 1 mg of freshly prepared protein lysate from Molt4 cells was added to the prepared beads in IP lysate buffer (50 mM Tris pH-7.5, 120 mM NaCl, 1% NP40, 0.5 mM EDTA, protease inhibitors, phosphatase inhibitors and NEM) for 1 h at 4 °C while rotating. The beads were washed three times with IP buffer and afterward used either for Western blotting or trypsin digestion followed by LC-MS/MS analysis.

#### Mass Spectrometry Sample Preparation

Protein-bound flag-CRBN beads were incubated with 20 μL SDC buffer (3% sodium deoxycholate in 50 mM Tris-HCl, pH 8.5) and heated for 5 min at 65 °C, and the supernatant was collected. This step was repeated one more time to get an elute of 40 μL. Further, reduction and alkylation were performed using 5 μL of 5 mM TCEP, 20 mM CAA in 50 mM Tris-HCl, pH 8.5, at 95 °C for 10 min. 500 ng of trypsin in 45 μL 50 mM Tris-HCl (pH 8.5) was added to each sample and kept for digestion at 37 °C overnight. The digestion was stopped upon the addition of 150 μL of 1% TFA in isopropanol. Peptide cleanup was performed using SDB-RPS stage tips (Sigma-Aldrich). Peptides were added to stage tips and washed first with 1% TFA in isopropanol and then with 0.2% TFA in water. Lastly, peptides were eluted in 80% acetonitrile plus 1.25% ammonia and dried in a vacuum concentrator.

#### Mass Spectrometry Data Acquisition

Dried peptides were resuspended in 2%ACN with 0.1% TFA and used for LC-MS/MS analysis on a QExactive HF mass spectrometer coupled to an easy nLC 1200 (Thermo Fisher Scientific) fitted with a 35 cm long, 75 μm ID fused-silica column packed in-house with 1.9 μm C18 particles (Reprosil pur, Dr. Maisch). The column was maintained at 40 °C using an integrated column oven (Sonation). Peptides were eluted in a nonlinear gradient of 5–40% acetonitrile over 60 min and sprayed into the mass spectrometer equipped with a nanoFlex ion source (Thermo Fisher Scientific). Full-scan MS spectra (300–1,650 *m*/*z*) were acquired in profile mode at a resolution of 60,000 at *m*/*z* 200, a maximum injection time of 20 ms, and an AGC (automatic gain control) target value of 3 × 10^6^. Up to 10 of the most intense peptides per full scan were isolated using a 1.4-Th window for fragmentation by higher energy collisional dissociation (normalized collision energy of 27). MS/MS spectra were acquired in centroid mode with a resolution of 30,000, a maximum injection time of 54 ms, and an AGC target value of 1 × 10^5^. Single charged ions, ions with a charge state of more than seven, and ions with unassigned charge states were not considered for fragmentation, and dynamic exclusion was set to 20 s to minimize the acquisition of fragment spectra representing already acquired precursors.

#### Mass Spectrometry Data Analysis

MS raw data were analyzed using FragPipe v21.1, with MSFragger v.4.0 ^63^ and Philosopher v.5.1.0.^64^ The built-in workflow “LFQ-MBR” was used with a precursor mass tolerance of 20 ppm and fragment mass tolerance of 20 ppm. The human proteome database used by FP (ID: UP000005640, 20/08/2024) comprised 20,468 reviewed sequences only and their corresponding decoys, including common contaminant proteins. Identifications were filtered to obtain false discovery rates (FDR) below 1% for both peptide spectrum matches (minimum peptide length of 7) and proteins using a target-decoy strategy. For all searches, carbamidomethylated cysteine was set as a fixed modification and oxidation of methionine and N-terminal protein acetylation as variable modifications, allowing up to 3 modifications per peptide. Strict trypsin cleavage was set as the protein digestion rule. Label-free quantification was performed using IonQuant v.1.10.27.^65^ Data were further processed using FragPipe Analyst.^66^ Subsequently, the data were plotted in R using custom scripts.

### Molecular Modeling and Simulation

Initial structures were taken from published structures for LIMK1 with the LIMKi3 ligand (PDB: 8AAU), LIMK2 with the LIMKi3 ligand (PDB: 8WSW), and CRBN (PDB: 8OIZ). The ligand orientations of LIMKi3 in the binding pockets of LIMK1 and LIMK2 differ due to a rotated thiazole group. We propose that LIMKi3 binds in the same pose for both LIMK1 and LIMK2. Therefore, the LIMK1 structure with the rotated thiazole of LIMKi3 was used for LIMK2 by aligning LIMK2 onto the Cα positions of LIMK1 with LIMKi3, and saving the LIMK2 coordinates along with the LIMKi3 coordinates from LIMK1 in VMD.^67^

Proteins and ligands were further processed in Schrödinger (version 2024–02) using the standard procedure, LigPrep for the ligand and protein preparation for the proteins. THNAN69 was also built in Schrödinger and prepared with LigPrep. The resulting structures were used to build initial ternary structure models without steric clashes using protein_degrader_sampler.py from Schrödinger.^68^ 30 ternary complexes containing CRBN, NAN69 and LIMK1, and 50 ternary complex structures containing CRBN, NAN69 and LIMK2 were generated.

#### System Preparation for MD Simulations

Initially, GAFF force field parameters^69^ for NAN69 were assigned using ANTECHAMBER. Partial charges were determined using the RESP-fitting approach.^70^ The system was prepared using tleap as part of the AmberTools package.^71^ Ternary complexes were solvated with OPC water^72^ at a distance of at least 15 Å from the periodic, truncated octahedral box boundaries. Proteins were described with AMBER FF19SB^73^ and the PROTAC with GAFF. Systems were neutralized, and 150 mM NaCl excess salt was added. A Zn^2+^ ion was added to the corresponding binding site of CRBN, and the coordinating cysteines were negatively charged. Minimization and the first two equilibration steps were done with the Sander CPU code of AMBER22. Final equilibrations and production runs were performed using the pmemd.cuda GPU code of AMBER22.

#### Energy Minimization

Every system was first energy minimized, with a maximum of 1000 steps using the steepest descent algorithm, followed by 1000 steps using the conjugate gradient algorithm. Nonbonded interactions were truncated after 10 Å. Position restraints of 500 kcal/mol/Å^2^ were applied to Zn^2+^, the protein atoms, and PROTAC atoms.

In a second minimization, position restraints of 1000 kcal/mol/Å^2^ were applied to the same residues, except the cysteines at the Zn^2+^ binding site of CRBN. The third minimization was then performed with 5000 steps of the steepest descent algorithm and 5000 steps of the conjugate gradient algorithm. Backbone and PROTAC atoms were fixed with position restraints of 500 kcal/mol/Å^2^.

During the fourth minimization, weaker position restraints of 250 kcal/mol/Å^2^ were applied to the PROTAC and backbone atoms of CRBN. In the final minimization step, the system was relaxed without position constraints.

#### MD Equilibration

Systems were equilibrated in four steps:

(i) The Zn^2+^, protein, and ligand atoms were weakly fixed in the simulation boxes (k = 10.0 kcal/mol/Å^2^). The systems were heated from 0 to 300 K using Langevin dynamics with a collision frequency of γ = 1.0 ps^–1^ over 100 ps. Random velocities were drawn from a Maxwell–Boltzmann distribution. SHAKE^74^ was applied to hydrogen-containing bonds, enabling a time step of 2 fs.
(ii) The systems were equilibrated for 500 ps employing position restraints on the protein backbone atoms and the PROTAC (k = 25 kcal/mol/Å^2^). A constant temperature of 300 K was maintained using Langevin dynamics (γ = 1.0 ps^–1^). The Berendsen barostat,^75^ with a pressure relaxation time of τ = 2.0 ps, maintained the pressure at 1 bar. Random seeds were set at the beginning of the equilibrations.
(iii) The same equilibration parameters were used, except position restraints were applied only to the CRBN backbone atoms and PROTAC atoms.
(iv) Finally, the systems were free to relax within 5 ns using the previous equilibration parameters.

#### Production Run

Production runs of 510 ns for each complex were carried out with the previous equilibration parameters, i.e., 300 K were maintained using Langevin dynamics (γ=1.0/ps) and 1 bar using the Berendsen barostat (τ = 2.0 ps). MD simulations were performed in 20 ns (LIMK1) and 30 ns (LIMK2) simulation chunks with a time step of 2 fs. For LIMK1, the last 10 ns between 500 to 510 ns were simulated in a 10 ns chunk to get 510 ns simulations for both LIMK1 and LIMK2 ternary complexes. Each chunk started with the same velocities and coordinates as the previous production run chunk. A cutoff of 10 Å for nonbonded interactions was set. Shake was enabled for hydrogen-containing bonds. A new random seed was set at chunk to avoid synchronization artifacts.^76^ Atomic coordinates were wrapped in the primary box.

### Chemical Synthesis

All compounds were synthesized following procedures described in the Supporting Information. Detailed experimental conditions and characterization data are provided therein.

## Supplementary Material



## Notes

Synthetic schemes for all compounds (Schemes S1–S3); detailed experimental procedures and full synthetic chemistry descriptions, including reagents, reaction conditions, and compound characterization methods; ^1^H NMR spectra and mass spectrometry (ESI) data for all newly synthesized compounds; supplemental figures supporting the main text and primary figures of the manuscript, including control experiments, and extended data sets (Figures S1–S6), together with supplemental references (PDF)

## Notes

The mass spectrometry proteomics data have been deposited to the ProteomeXchange Consortium via the PRIDE^55^ partner repository with the data set identifiers PXD067272 (whole-cell proteome of MOLT-4 cells), PXD067224 (ProxiCapture of MOLT-4 cells), and PXD067130 (whole-cell proteome of HuCCT1 cells). Any additional information required to reanalyze the data reported in this paper is available from the lead contact upon request.
