# Supplementary material for: Discovery and Development of a Potent LIMK2 Isoform-Specific Degrader
Source: ACS Chem Biol. 2026 May 1;21(5):1158–76. doi: 10.1021/acschembio.6c00137 (PMC13184981; doi:10.1021/acschembio.6c00137)
Supplement: Supplementary file 1 [file cb6c00137_si_001.pdf]

Supplementary information (SI)

Discovery and Development of a Potent LIMK2 Isoform-specific Degrader

Kamal Rayees Abdul Azeez,[1,2,†] Hayuningbudi Saraswati,[3,†] Thorsten Mosler,[4] Thomas Hanke,[1,2] Hung Ho-Xuan,[4,5] Noah Neder,[1,2] Saran Aswathaman Sivashanmugam,[1,2] Marcel Heinz,[6] Martin-Peter Schwalm,[1,2] Giulio Giuliani,[4,5] Rajeshwari Rathore,[4] Rubina Kazi,[4] Rahul Kumar,[3] Marko Mitrovic,[1,2] Cristian Prieto-Garcia,[4] Henry J. Bailey,[4,5] Sebastian Mathea,[1,2] Gerhard Hummer,[6,7] Ivan Đikić,[4,5] Susanne Müller,[1,2] Alexandra Stolz,[4,5] Daniela S Krause,[3,8,9,‡] and Stefan Knapp[1,2,8,‡,*]

[1]Institute of Pharmaceutical Chemistry, Goethe University, Max-von-Laue-Str. 9, 60438 Frankfurt am Main, Germany
[2]Structural Genomics Consortium (SGC), Buchmann Institute for Life Sciences, Max-von-Laue-Str. 15, 60438 Frankfurt am Main, Germany
[3]Institute of Transfusion Medicine−Transfusion Centre, Johannes Gutenberg University Medical Center, 55131 Mainz, Germany
[4]Institute of Biochemistry II, School of Medicine, Goethe University Frankfurt, Frankfurt am Main 60590, Germany
[5]Buchmann Institute for Molecular Life Sciences (BMLS), Goethe University, Max-von-Laue-Str. 15, 60438 Frankfurt am Main, Germany
[6]Department of Theoretical Biophysics, Max Planck Institute of Biophysics, Frankfurt am Main 60438, Germany
[7]Institute for Biophysics, Goethe University Frankfurt, Frankfurt am Main 60438, Germany
[8]German Cancer Consortium (DKTK) Mainz/Frankfurt, German Cancer Research Center (DKFZ), 69120 Heidelberg, Germany
[9]Research Center for Immunotherapy (FZI), University Medical Center, University of Mainz, 55131 Mainz, Germany
[†] These authors contributed equally
[‡] These authors contributed equally as co-senior authors
*Correspondence: knapp@pharmchem.uni-frankfurt.de

TABLE OF CONTENTS

SECTIONS		PAGE
**SCHEMES AND FIGURES**		
**Schemes/Figures**	**Title / Description**	
Scheme S1	Synthesis of LIMKi3 azide handles	S3
Scheme S2	Synthesis of CRBN and VHL alkyne handles	S3
Scheme S3	Synthesis of PROTACs	S4
Figure S1	Physicochemical properties of synthesized PROTACs	S5
Figure S2	Biophysical characterization and cellular target engagement of the synthesized PROTACs	S6
Figure S3	Screening and initial assessment of the degradation potency of developed PROTACs	S10
Figure S4	Cellular characterization of PROTACs and control compounds	S11
Figure S5	Assessment of CYBA and DGUOK downregulation	S12
Figure S6	Structural assessment of LIMK2 isoform specificity	S13
**EXPERIMENTAL SECTION**		
Chemical synthesis: general procedures		S18
A) Synthesis of LIMKi3 azide handles		S19
B) Synthesis of CRBN and VHL alkyne handles		S27
C) Synthesis of LIMK-PROTACs		S34
**REFERENCES**		S51

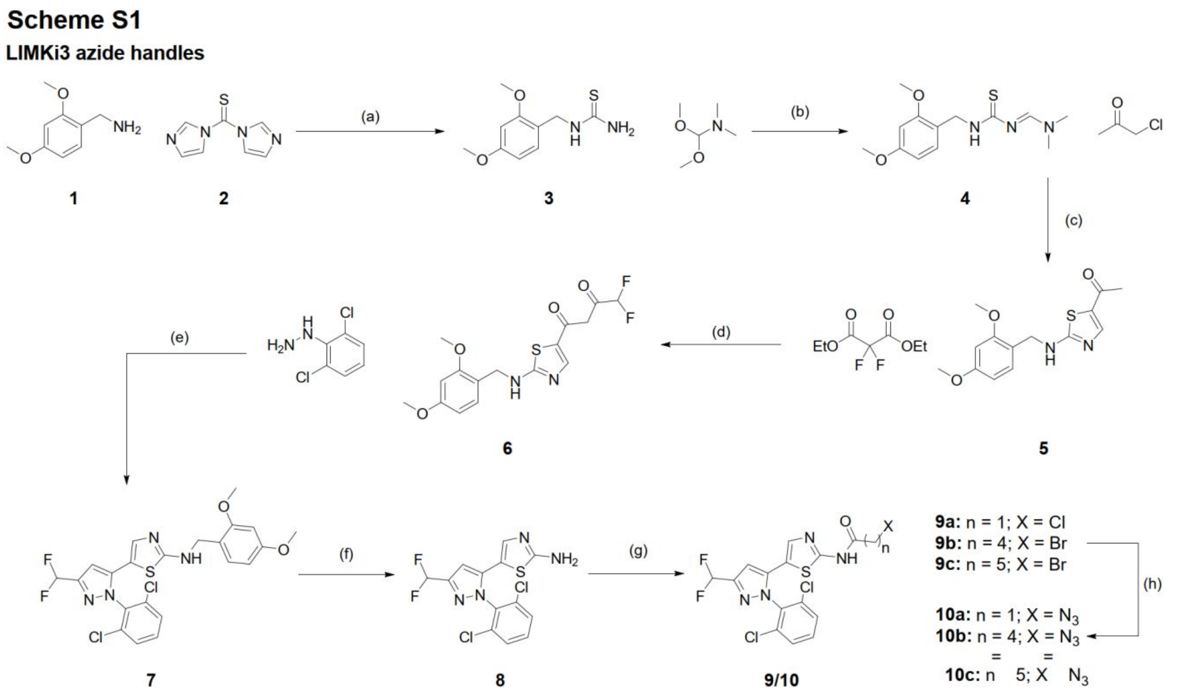

Scheme S1. Synthesis of LIMKi3 Building Block with Azide Linkers.
Reagents and conditions: (a) i) DCM, RT, 3 h; ii) NH_3, MeOH, RT, 16 h; (b) EtOH, 80 °C, 1 h; (c) MeCN, 75 °C, 3 h; (d) NaOEt, EtOH, 75 °C, 5 h; (e) EtOH, 75 °C, 20 h; (f) H_2O/TFA, RT, 20 h; (g) Acyl chloride, pyridine, DCM, RT, 16 h; (h) NaN_3, acetone, RF, 16 h.

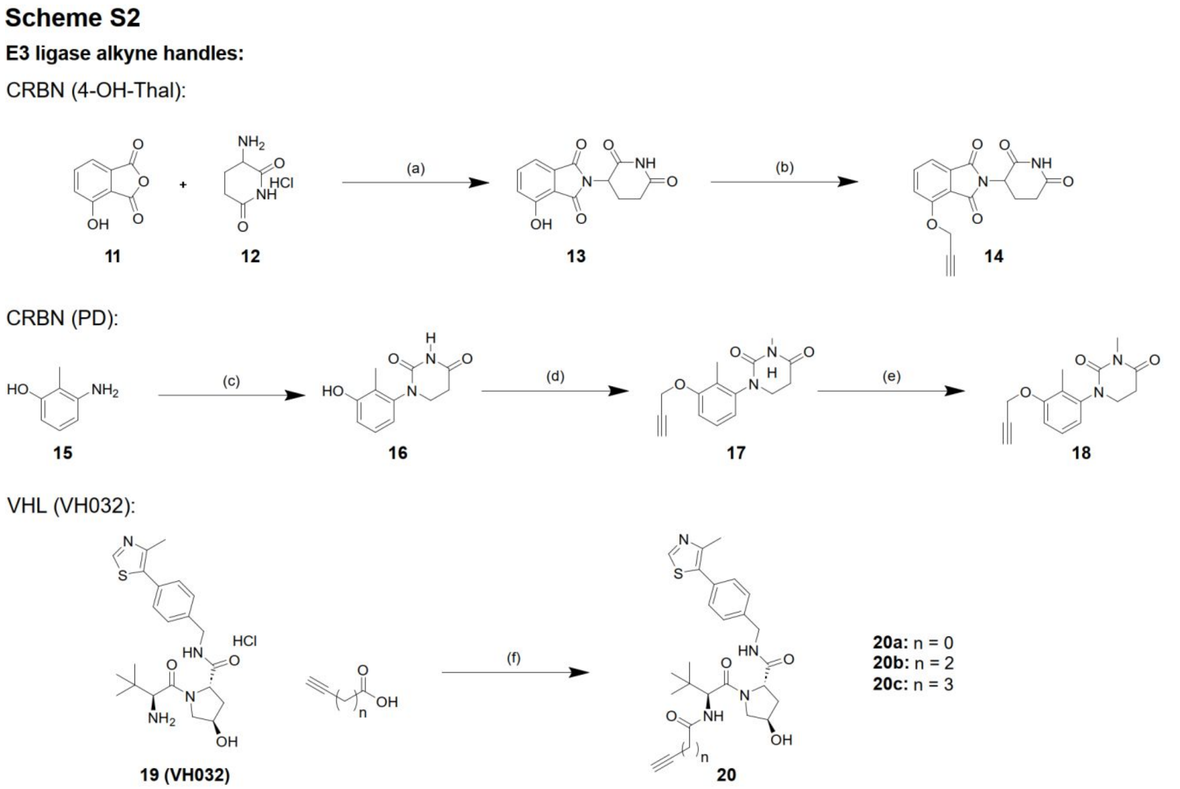

Scheme S2. Synthesis of CRBN and VHL Handles with Terminal Alkyne Linkers.

Reagents and conditions: (a) NaOAc, AcOH, 110 °C, 12 h; (b) Propargyl bromide, Na_2CO_3, DMF, 60 °C, 3 h; (c) i) Acrylic acid, toluene, 110 °C, 3 h; ii) Urea, AcOH, 110 °C, 18 h; (d) Propargyl bromide, K_2CO_3, DMF, RT, 2 h; (e) CH_3I, Cs_2CO_3, DMF, RT, 18 h; (f) DIPEA, HATU, DMF, RT, 3h.

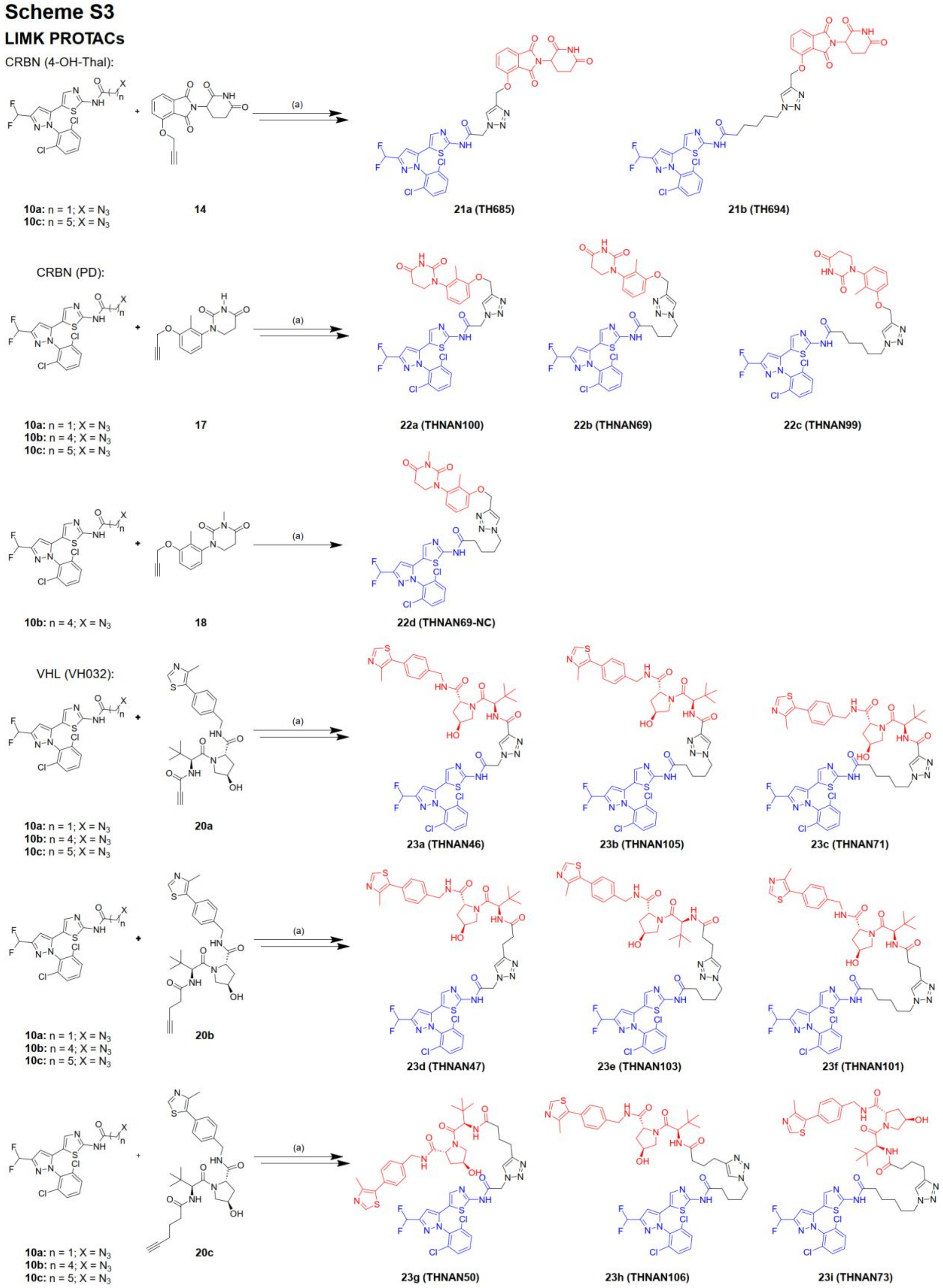

Scheme S3. Synthesis of LIMK-PROTACs.

Reagents and conditions: (a) $CuSO_4$ * 5 H_2O, NaAsc, THF/H_2O, RT, 16 h.

ID	Molecule	MW (Da)	LogP	pKa-ma	pKa-mb	PSA	LogD	HBA	HBD	RotB	SMILES
LIMKi3	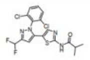	430.0	5.5	11.5	1.2	71.5	5.5	4	1	6	CC(C)C(Nc1ncc(c2cc(C(F)F)nn2c2c(cccc2[Cl])[<br>Cl])s1)=O
**21b**	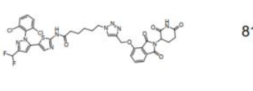	811.1	3.7	11.5	1.9	263.1	3.7	15	2	15	C(CCC(Nc1ncc(c2cc(C(F)F)nn2c2c(cccc2[Cl])[Cl])<br>s1)=O)CCn1cc(<br>COc2cccc3C(N(C4CCC(NC4=O)=O)C(c23)=O)=O<br>)nn1
**21a**	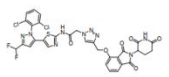	755.1	3.2	11.7	3.4	273.9	3.2	15	2	11	C1CC(NC(C1N1C(c2cccc(c2C1=O)OCc1cn(CC(N<br>c2ncc(c3cc(C(F)<br>F)nn3c3c(cccc3[Cl])[Cl])s2)=O)nn1)=O)=O)=O
**22a**	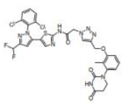	701.1	5.2	10.0	3.4	214.1	5.2	11	2	11	Cc1c(cccc1OCc1cn(CC(Nc2ncc(c3cc(C(F)F)nn3c3<br>c(cccc3[Cl])[Cl])s2)=O)nn1)N1CCC(NC1=O)=O
**22b**	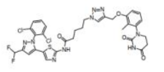	743.1	5.2	10.0	1.9	203.7	5.2	11	2	14	Cc1c(cccc1OCc1cn(CCCCC(Nc2ncc(c3cc(C(F)F)<br>nn3c3c(cccc3[Cl]<br>)[Cl])s2)=O)nn1)N1CCC(NC1=O)=O
**22c**	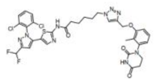	757.2	5.7	10.0	1.9	203.3	5.7	11	2	15	Cc1c(cccc1OCc1cn(CCCCCC(Nc2ncc(c3cc(C(F)F<br>)nn3c3c(cccc3[C<br>l])[Cl])s2)=O)nn1)N1CCC(NC1=O)=O
**23i**	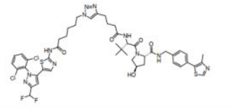	1023.3	6.2	11.5	2.8	246.7	6.2	14	4	25	Cc1c(c2ccc(CNC([C@@H]3C[C@H](CN3C([C@<br>H](C(C)(C)C)NC(<br>CCCc3cn(CCCCCC(Nc4ncc(c5cc(C(F)F)nn5c5c(c<br>ccc5[Cl])[Cl])s4) =O)nn3)=O)=O)O)=O)cc2)scn1
**23c**	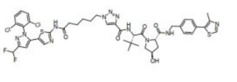	981.3	5.9	11.5	2.8	243.5	5.9	14	4	22	Cc1c(c2ccc(CNC([C@@H]3C[C@H](CN3C([C@H<br>](C(C)(C)C)NC(c<br>3cn(CCCCCC(Nc4ncc(c5cc(C(F)F)nn5c5c(cccc5[<br>Cl])[Cl])s4)=O)n n3)=O)=O)O)=O)cc2)scn1
**23f**	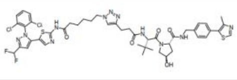	1009.3	5.8	11.5	4.1	249.1	5.8	14	4	24	Cc1c(c2ccc(CNC([C@@H]3C[C@H](CN3C([C@<br>H](C(C)(C)C)NC(<br>CCc3cn(CCCCCC(Nc4ncc(c5cc(C(F)F)nn5c5c(cc<br>cc5[Cl])[Cl])s4)= O)nn3)=O)=O)O)=O)cc2)scn1
**23h**	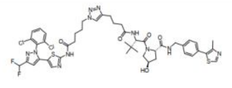	1009.3	5.7	11.5	2.8	247.0	5.7	14	4	24	Cc1c(c2ccc(CNC([C@@H]3C[C@H](CN3C([C@<br>H](C(C)(C)C)NC(<br>CCCc3cn(CCCCC(Nc4ncc(c5cc(C(F)F)nn5c5c(cc<br>cc5[Cl])[Cl])s4)= O)nn3)=O)=O)O)=O)cc2)scn1
**23g**	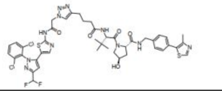	967.2	5.6	11.7	3.4	257.4	5.6	14	4	21	Cc1c(c2ccc(CNC([C@@H]3C[C@H](CN3C([C@H<br>](C(C)(C)C)NC(<br>CCCc3cn(CC(Nc4ncc(c5cc(C(F)F)nn5c5c(cccc5[C<br>l])[Cl])s4)=O)nn 3)=O)=O)O)=O)cc2)scn1
**23b**	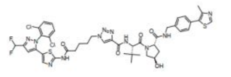	967.2	5.4	11.5	2.8	243.9	5.4	14	4	21	Cc1c(c2ccc(CNC([C@@H]3C[C@H](CN3C([C@H<br>](C(C)(C)C)NC(c<br>3cn(CCCCC(Nc4ncc(c5cc(C(F)F)nn5c5c(cccc5[Cl]<br>)[Cl])s4)=O)nn 3)=O)=O)O)=O)cc2)scn1
**23a**	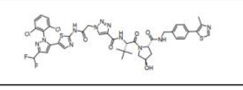	925.2	5.4	11.8	3.4	254.2	5.4	14	4	18	Cc1c(c2ccc(CNC([C@@H]3C[C@H](CN3C([C@H<br>](C(C)(C)C)NC(c<br>3cn(CC(Nc4ncc(c5cc(C(F)F)nn5c5c(cccc5[Cl])[Cl])<br>s4)=O)nn3)=O)=O)O)=O)cc2)scn1
**23e**	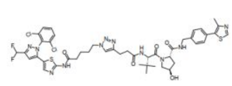	995.3	5.3	11.5	4.1	249.4	5.3	14	4	23	Cc1c(c2ccc(CNC([C@@H]3C[C@H](CN3C([C@H<br>](C(C)(C)C)NC(<br>CCc3cn(CCCCC(Nc4ncc(c5cc(C(F)F)nn5c5c(cccc<br>5[Cl])[Cl])s4)=O) nn3)=O)=O)O)=O)cc2)scn1
**23d**	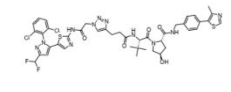	953.2	5.3	11.7	3.4	259.8	5.3	14	4	20	Cc1c(c2ccc(CNC([C@@H]3C[C@H](CN3C([C@H<br>](C(C)(C)C)NC(<br>CCc3cn(CC(Nc4ncc(c5cc(C(F)F)nn5c5c(cccc5[Cl])<br>[Cl])s4)=O)nn3)=O)=O)O)=O)cc2)scn1

Figure S1. Physicochemical Properties of LIMK2 PROTACs.

A

Compound	Average (°C)	SD	T_m shift (°C)
DMSO	46.5		
LIMKi3	53.5	1.5	7.0
21a	52.5	2.1	6.0
21b (TH694)	51.9	0.1	5.3
22a	52.5	0.5	6.0
22b (THNAN69)	53.5	0.4	6.9
22c	52.5	0.3	6.0
23a	51.2	0.2	4.7

Compound	Average (°C)	SD	T_m shift (°C)
23b	49.8	0.4	3.3
23c	48.3	0.6	1.8
23d	51.2	0.9	4.7
23e	52.5	0.5	6.0
23f	51.7	0.5	5.1
23g	51.3	0.4	4.7
23h	52.6	0.8	6.1
23i	52.2	1.3	5.6

B

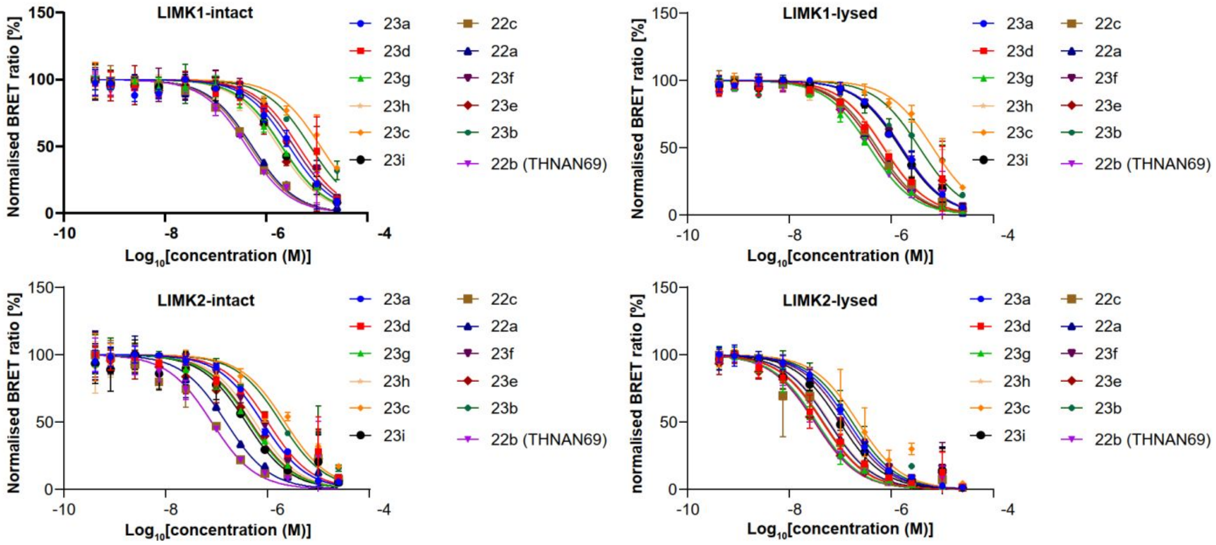

VHL-C3: IC_{50} (nM)

PROTAC	LIMKi3 azide	LIMK1-intact	LIMK1-lysed	LIMK2-intact	LIMK2-lysed
**23a**	LIMKi3-C2	2832	1568	799	143
**23b**	LIMKi3-C5	7405	3555	1826	171
**23c**	LIMKi3-C6	11830	6517	2192	220

PD-C3: IC_{50} (nM)

PROTAC	LIMKi3 azide	LIMK1-intact	LIMK1-lysed	LIMK2-intact	LIMK2-lysed
**22a**	LIMKi3-C2	528	527	153	58
**22b**	LIMKi3-C5	412	377	80	32
**22c**	LIMKi3-C6	476	532	80	47

VHL-C5: IC_{50} (nM)

PROTAC	LIMKi3 azide	LIMK1-intact	LIMK1-lysed	LIMK2-intact	LIMK2-lysed
**23d**	LIMKi3-C2	4347	701	1013	44
**23e**	LIMKi3-C5	2029	465	470	31
**23f**	LIMKi3-C6	3535	1608	796	122

VHL-C6: IC_{50} (nM)

PROTAC	LIMKi3 azide	LIMK1-intact	LIMK1-lysed	LIMK2-intact	LIMK2-lysed
**23g**	LIMKi3-C2	2016	377	444	34
**23h**	LIMKi3-C5	1733	732	567	62
**23i**	LIMKi3-C6	2017	1480	380	96

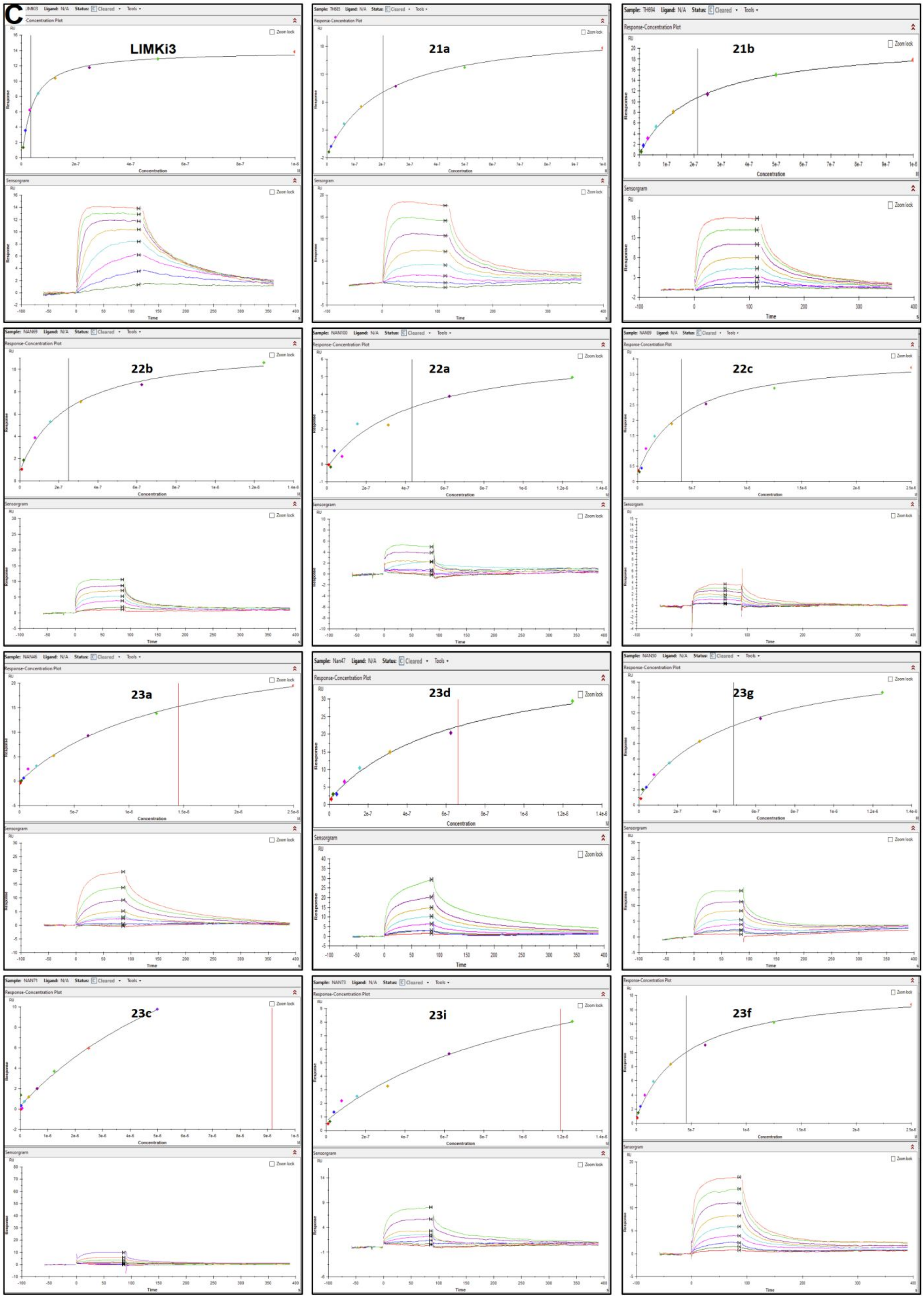

C
LIMKi3
21a
21b
22b
22a
22c
23a
23d
23g
23c
23i
23f

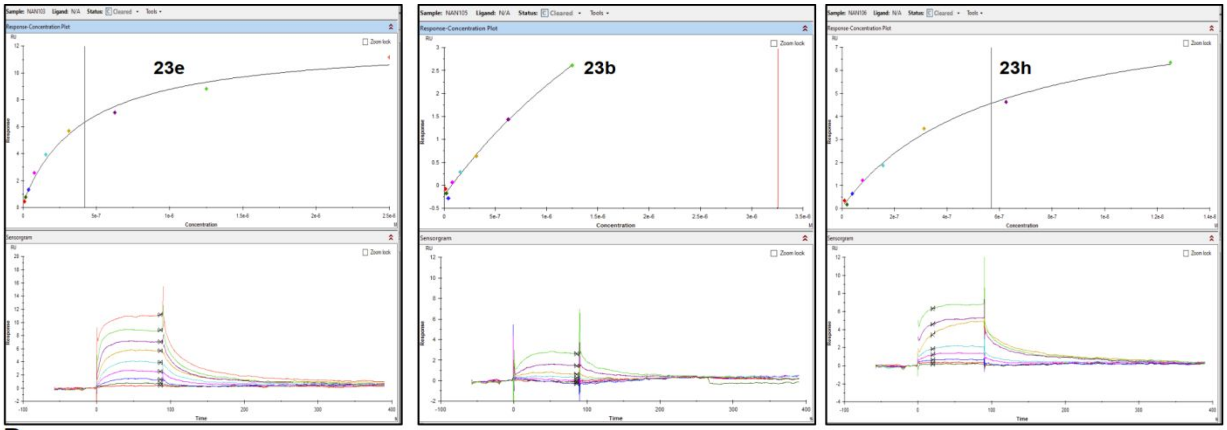

D

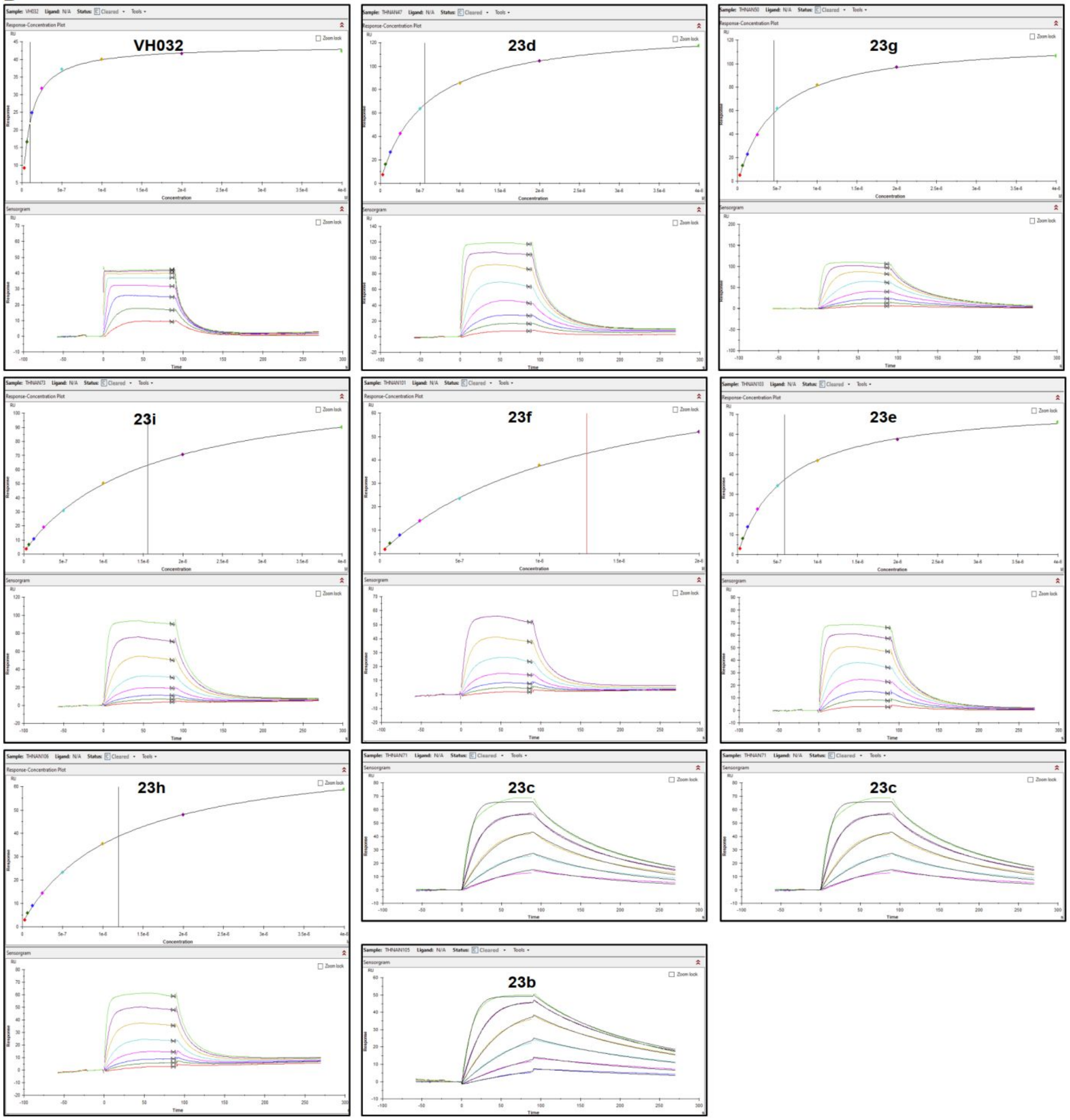

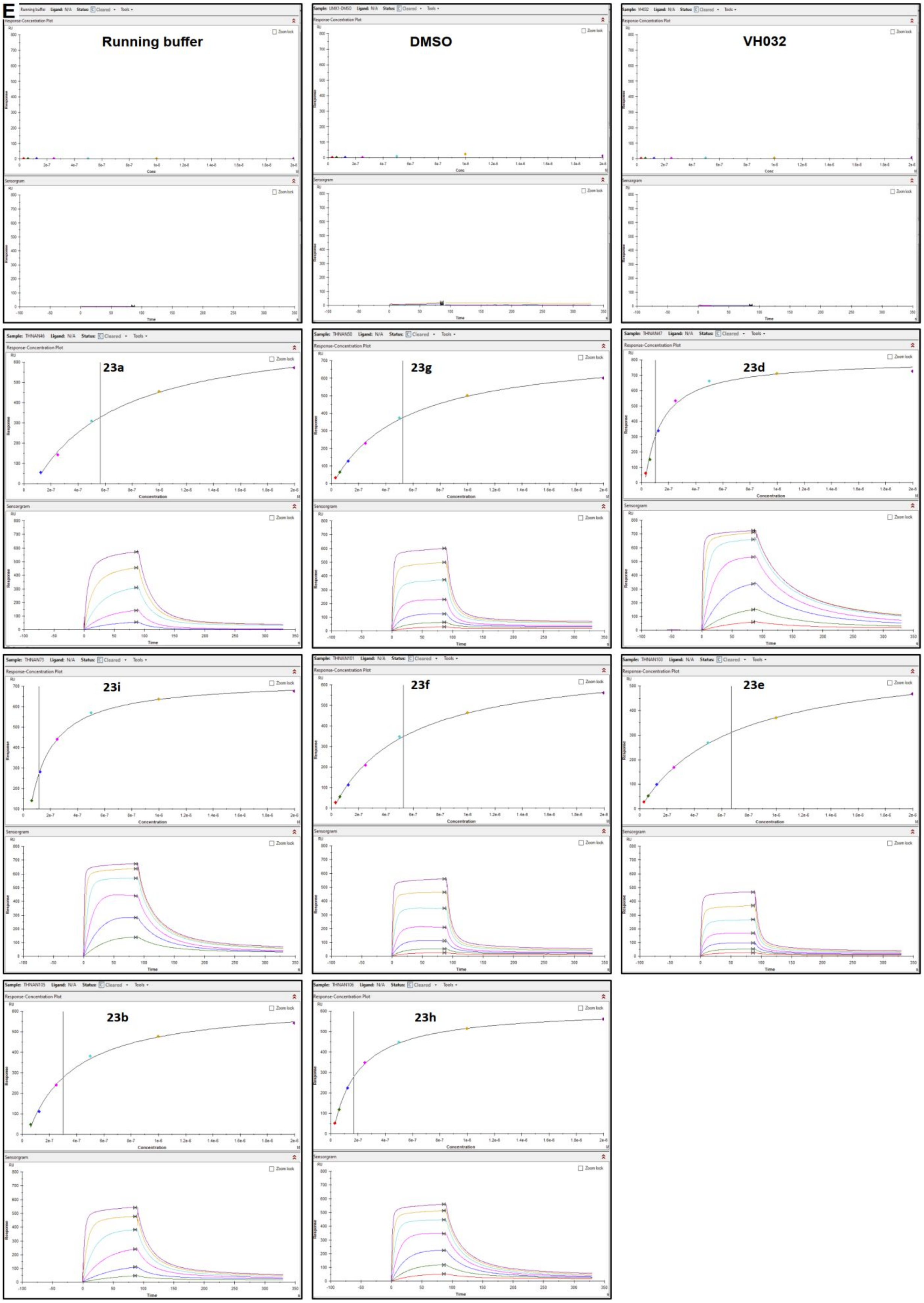
E
Running buffer
DMSO
VH032
23a
23g
23d
23i
23f
23e
23b
23h

Figure S2: Biophysical Characterization and Cellular Target Engagement of the Synthesized PROTACs.
(A) Differential scanning fluorimetry (DSF) melt curve temperature shifts (ΔT_m) for LIMK1.
(B) NanoBRET IC_{50} curves for LIMK1/2:PROTAC binary complex. Data represent mean ± SEM from duplicate experiments conducted in both intact and permeabilized cell modes. Corresponding IC_{50} values are summarized.
(C) LIMK1:PROTAC binary complex sensorgrams and K_D plots. Related to Figure 2A. Binary complex of all PROTACs to LIMK1 measured by SPR, with LIMK1 immobilized on the chip surface. K_D values were determined using the steady-state 1:1 binding model. Comparative data, including LIMKi3, are summarized in Figure 2A.
(D–E) VHL:PROTAC binary and ternary complex analysis by SPR.
(D) Binary complex affinity of all PROTACs measured by SPR with VHL:ELOB:ELOC immobilised on the chip. K_D values were determined using the steady-state 1:1 binding model; kinetic model fitting was used for THNAN50, THNAN71, and THNAN105.
(E) Ternary complex formation assessed by SPR with VHL:ELOB:ELOC immobilised on the chip. K_D values were fitted as described in (D) and are summarized in Figure 2B.

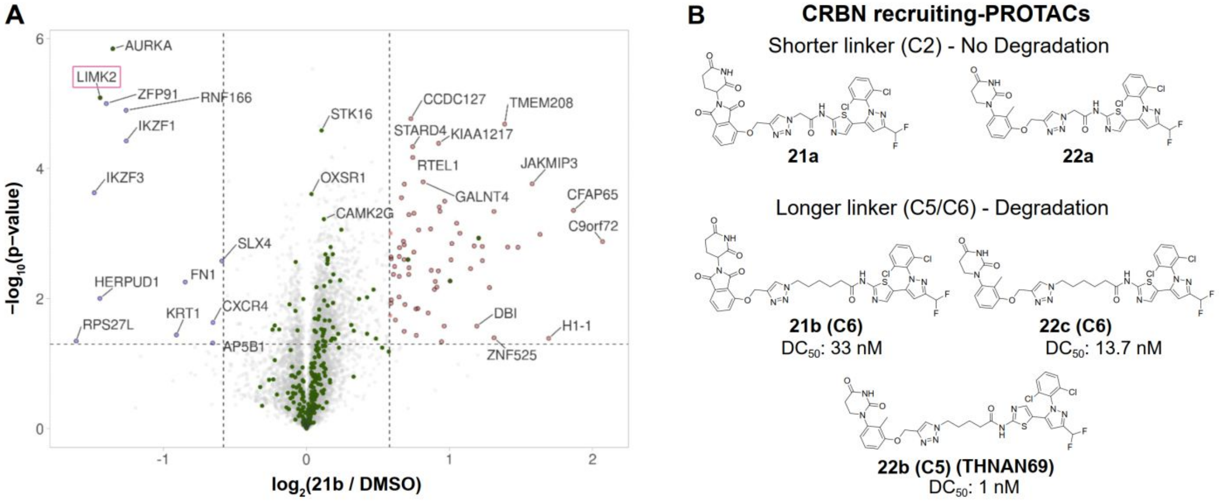

Figure S3. Screening and Initial Assessment of the Degradation Potency of Developed PROTACs.
(A) Quantitative proteomics analysis of 21b in the MOLT-4 cell line (6 h). Volcano plot from quantitative proteomics following 1 µM PROTAC treatment, showing isoform-specific degradation of LIMK2 (red box) and no downregulation of CYBA and DGUOK in this cell line. Significantly upregulated and downregulated proteins are labeled.
(B) Impact of linker length on degradability of CRBN-recruiting PROTACs. Lewis structures of PROTACs, with the aliphatic linker length flanking the triazole indicated in parentheses, and their respective calculated DC_{50} values are shown.

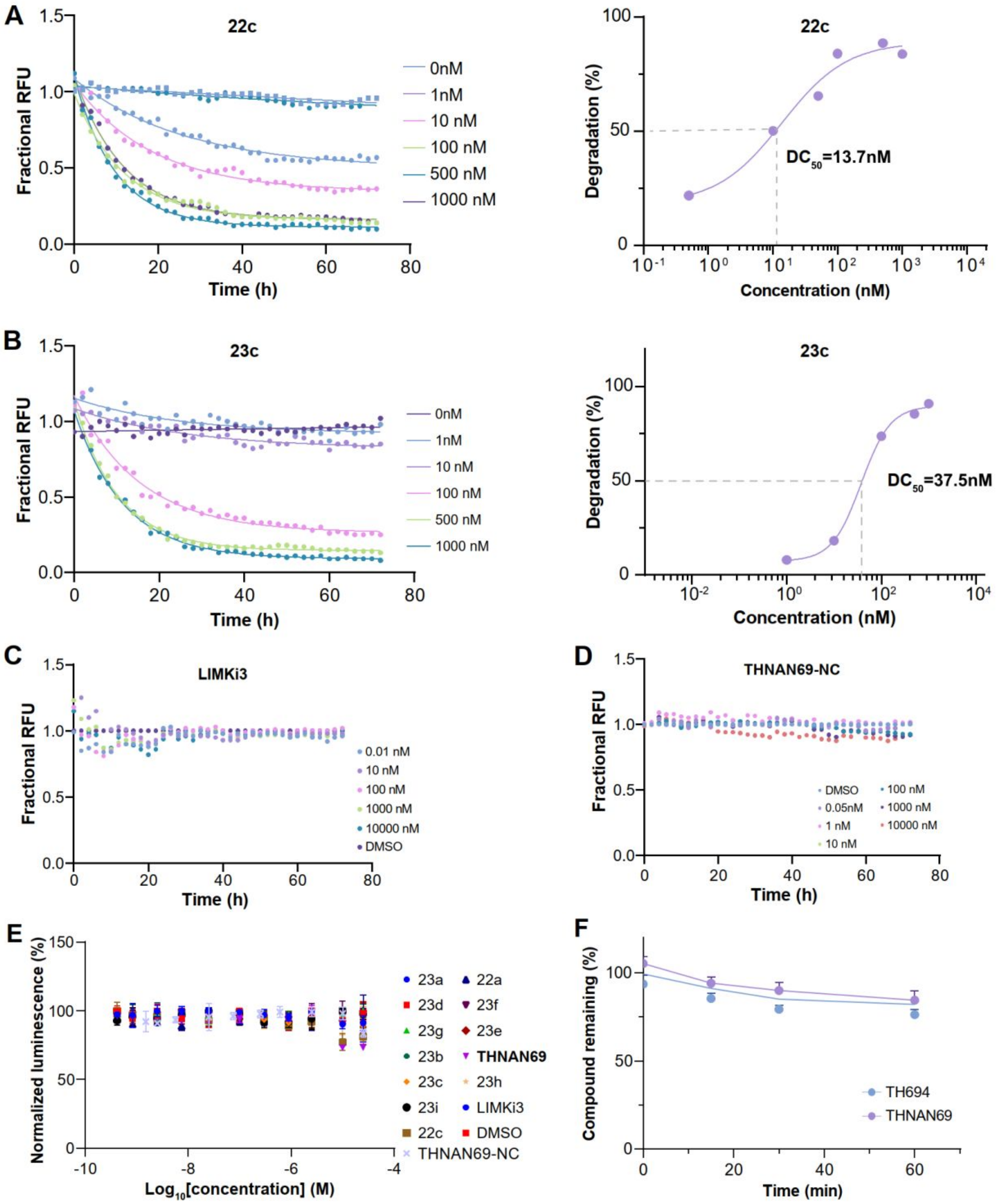

Figure S4. Cellular Characterization of PROTACs and Control Compounds.

(A–B) EGFP–LIMK2 degradation kinetics and DC_{50} plots for PROTACs 22c and 23c, as previously described. LIMK2-EGFP degradation assay was performed using an extended concentration range. Fractional relative fluorescence units (RFU) are plotted over time (left panels), and DC_{50} was calculated (right panels).

(C–D) LIMK2–EGFP depletion assay using an extended concentration range of LIMKi3 (left panel) and THNAN69-NC (right panel). Fractional relative fluorescence units (RFU) are plotted over time. Fluorescence traces remain stable over a 72-h period, indicating no degradation of LIMK2 by either LIMKi3 or the negative control THNAN69-NC.

(E) Cell viability assay using CellTiter-Glo® 2.0 viability assay (24 h). Viability was measured following compound exposure over a concentration range of 25 nM to 20 µM. No significant cytotoxicity was observed, as indicated by a stable signal across the tested range.
(F) 21b and THNAN69 have favorable metabolic stability. Metabolic stability was evaluated using rat liver microsomes. The percentage of parent PROTAC remaining over time was quantified by HPLC.

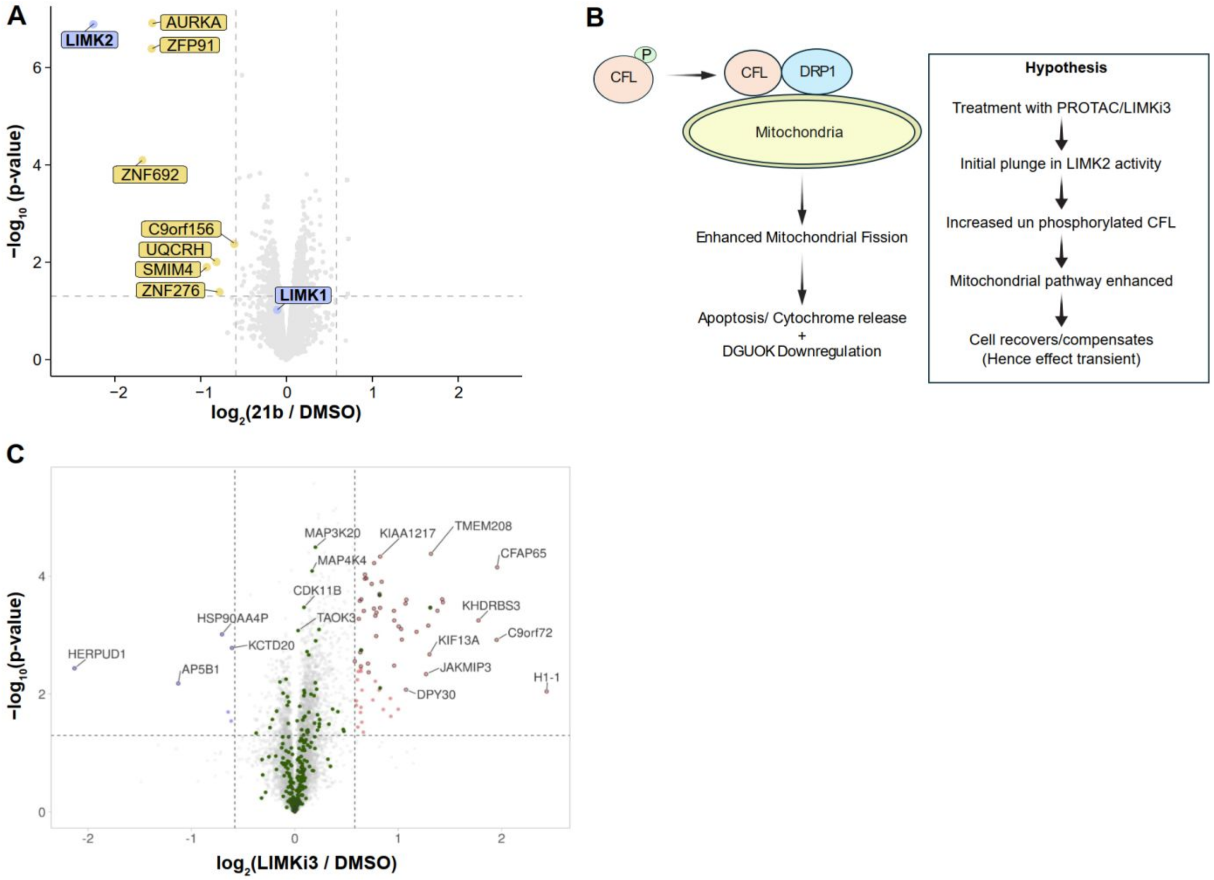

Figure S5. CYBA and DGUOK Downregulation.
(A) CYBA and DGUOK downregulation was transient in the HuCTT1 cell line. Volcano plot of quantitative proteomics analysis following 500nM 21b PROTAC treatment, analyzed after 24 h. Neither CYBA nor DGUOK was downregulated at this time point, while LIMK2 degradation persisted. Significantly downregulated proteins are labeled in yellow.
(B) Conceptual scheme for CYBA and DGUOK downregulation. Schematic depicting the proposed transient mechanism responsible for the observed downregulation of CYBA and DGUOK in the HuCTT1 cell line.
(C) LIMKi3 did not downregulate CYBA and DGUOK in the MOLT-4 cell line (6 h). Volcano plot of quantitative proteomics analysis following 1 µM LIMKi3 treatment. Neither CYBA nor DGUOK was detected in this cell line. Significantly upregulated and downregulated proteins are labeled.

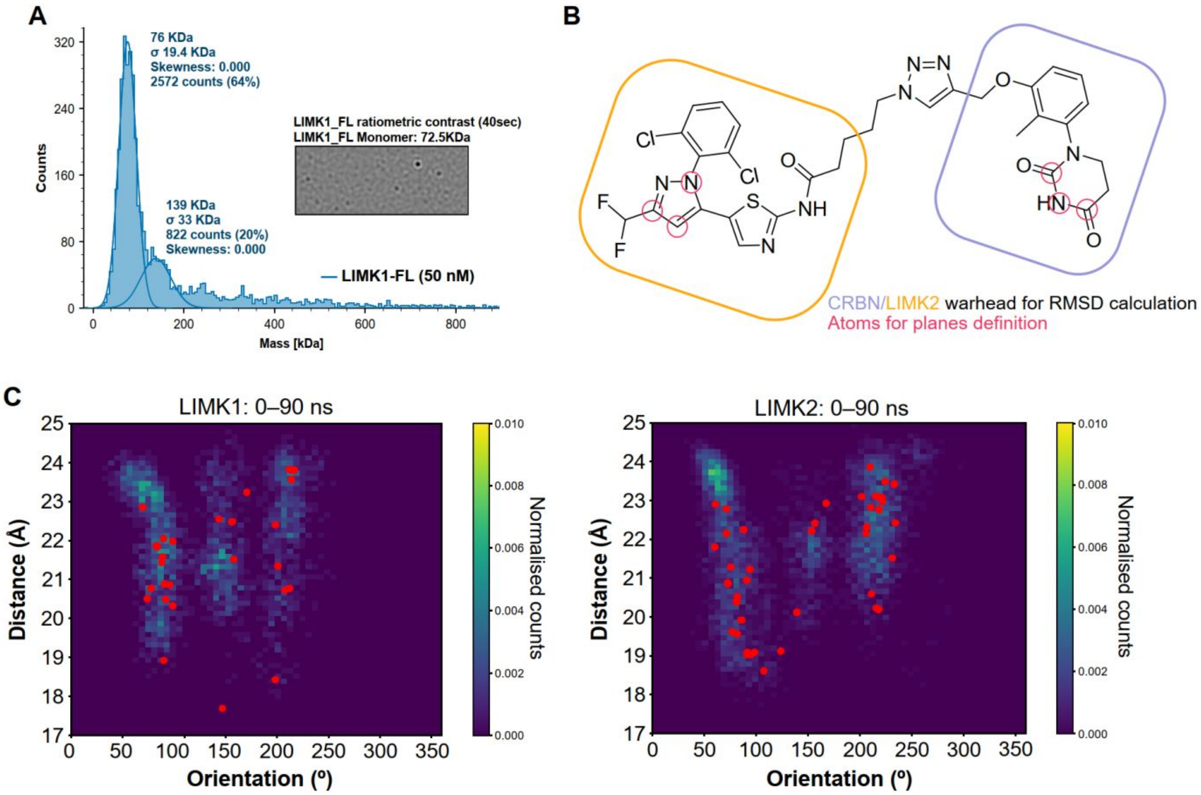
A
76 KDa
σ 19.4 KDa
Skewness: 0.000
2572 counts (64%)
139 KDa
σ 33 KDa
822 counts (20%)
Skewness: 0.000
LIMK1_FL ratiometric contrast (40sec)
LIMK1_FL Monomer: 72.5KDa
LIMK1-FL (50 nM)
Counts
Mass [kDa]
B
CRBN/LIMK2 warhead for RMSD calculation
Atoms for planes definition
C
LIMK1: 0–90 ns
LIMK2: 0–90 ns
Distance (Å)
Orientation (º)
Normalised counts

D

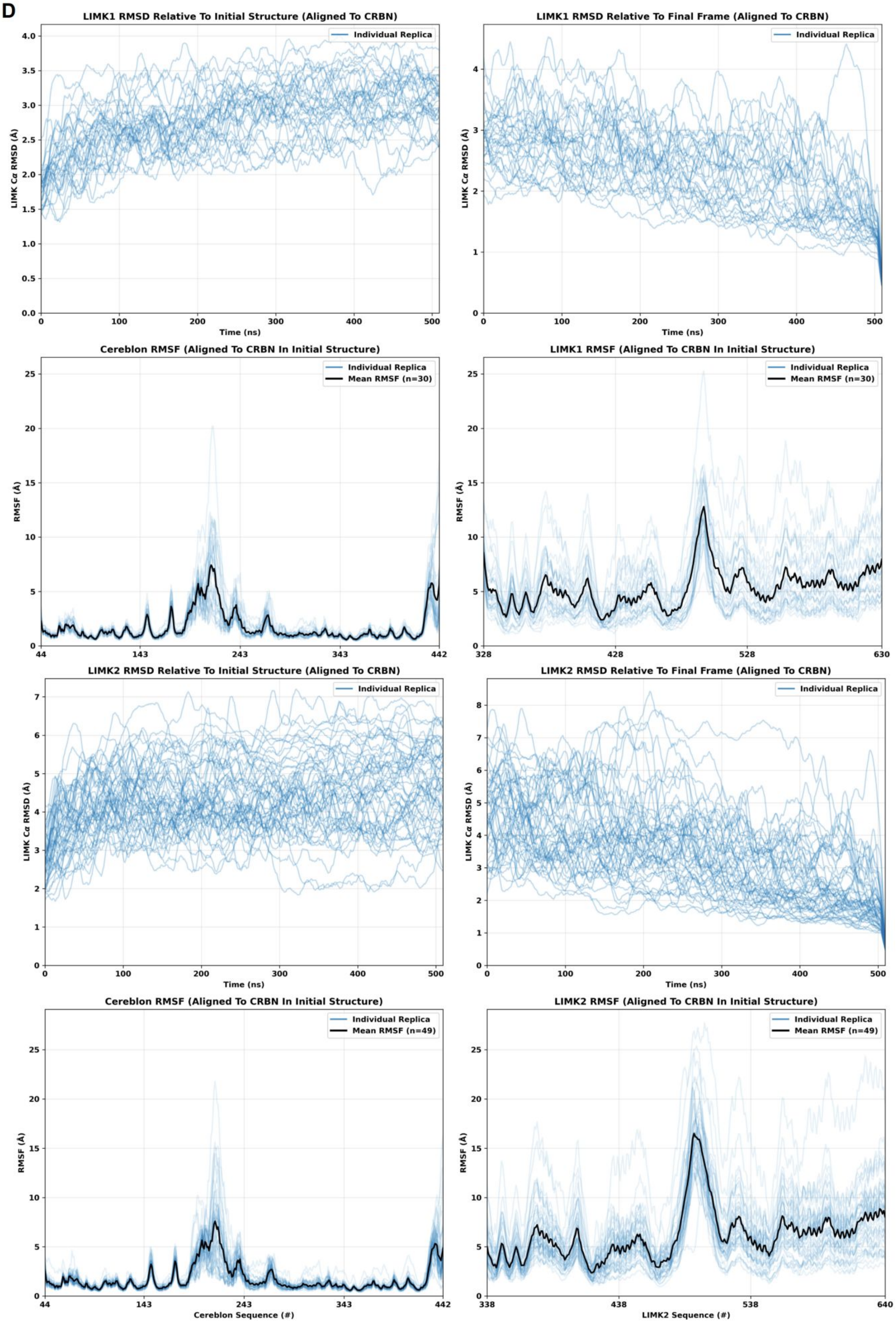

LIMK1 RMSD Relative To Initial Structure (Aligned To CRBN)
Individual Replica
LIMK Cα RMSD (Å)
Time (ns)
LIMK1 RMSD Relative To Final Frame (Aligned To CRBN)
Individual Replica
LIMK Cα RMSD (Å)
Time (ns)
Cereblon RMSF (Aligned To CRBN In Initial Structure)
Individual Replica
Mean RMSF (n=30)
RMSF (Å)
LIMK1 RMSF (Aligned To CRBN In Initial Structure)
Individual Replica
Mean RMSF (n=30)
RMSF (Å)
LIMK2 RMSD Relative To Initial Structure (Aligned To CRBN)
Individual Replica
LIMK Cα RMSD (Å)
Time (ns)
LIMK2 RMSD Relative To Final Frame (Aligned To CRBN)
Individual Replica
LIMK Cα RMSD (Å)
Time (ns)
Cereblon RMSF (Aligned To CRBN In Initial Structure)
Individual Replica
Mean RMSF (n=49)
RMSF (Å)
Cereblon Sequence (#)
LIMK2 RMSF (Aligned To CRBN In Initial Structure)
Individual Replica
Mean RMSF (n=49)
RMSF (Å)
LIMK2 Sequence (#)

E

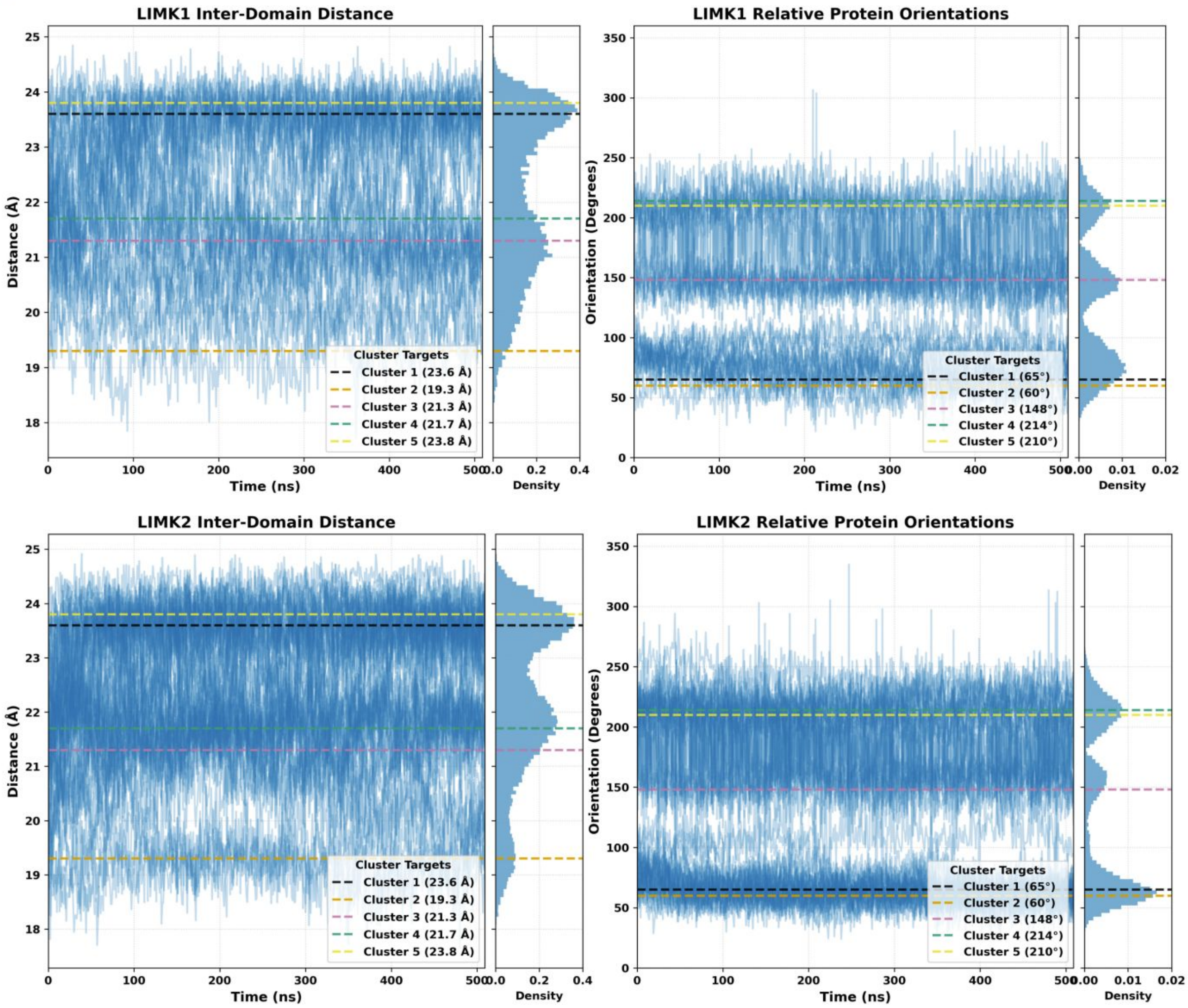
LIMK1 Inter-Domain Distance
Distance (Å)
Time (ns)
Density
Cluster Targets
Cluster 1 (23.6 Å)
Cluster 2 (19.3 Å)
Cluster 3 (21.3 Å)
Cluster 4 (21.7 Å)
Cluster 5 (23.8 Å)
LIMK1 Relative Protein Orientations
Orientation (Degrees)
Time (ns)
Density
Cluster Targets
Cluster 1 (65°)
Cluster 2 (60°)
Cluster 3 (148°)
Cluster 4 (214°)
Cluster 5 (210°)
LIMK2 Inter-Domain Distance
Distance (Å)
Time (ns)
Density
Cluster Targets
Cluster 1 (23.6 Å)
Cluster 2 (19.3 Å)
Cluster 3 (21.3 Å)
Cluster 4 (21.7 Å)
Cluster 5 (23.8 Å)
LIMK2 Relative Protein Orientations
Orientation (Degrees)
Time (ns)
Density
Cluster Targets
Cluster 1 (65°)
Cluster 2 (60°)
Cluster 3 (148°)
Cluster 4 (214°)
Cluster 5 (210°)

F

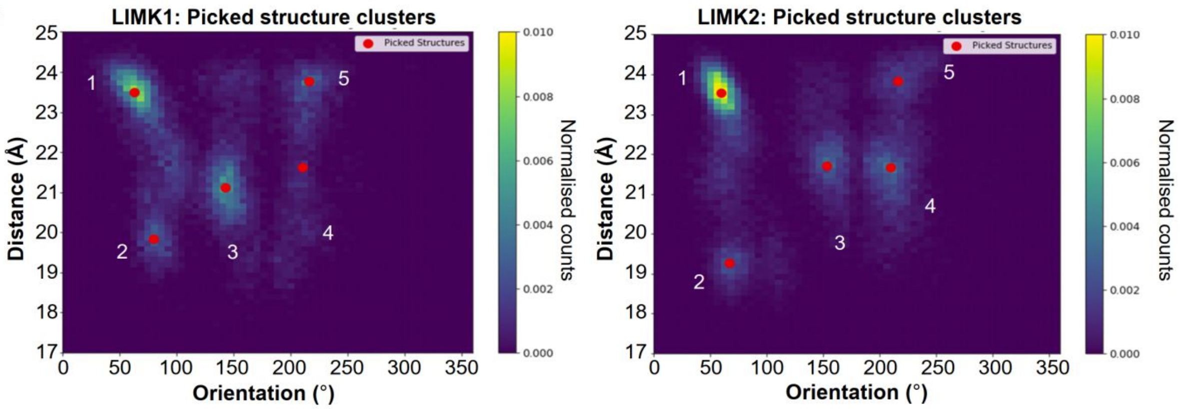
LIMK1: Picked structure clusters
Picked Structures
Distance (Å)
Orientation (°)
Normalised counts
1
2
3
4
5
LIMK2: Picked structure clusters
Picked Structures
Distance (Å)
Orientation (°)
Normalised counts
1
2
3
4
5

G

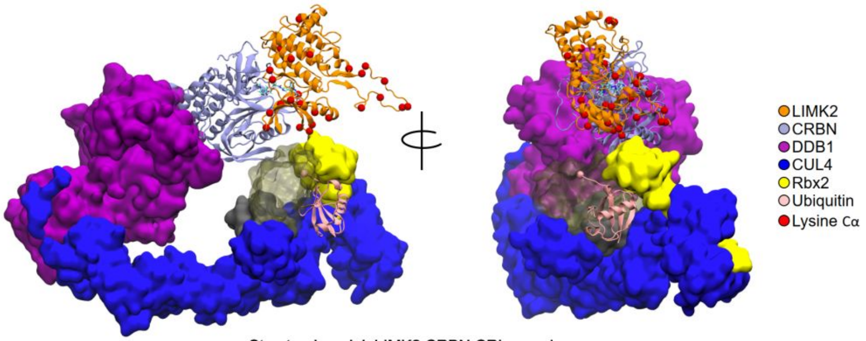

Strcutural model: LIMK2:CRBN CRL complex

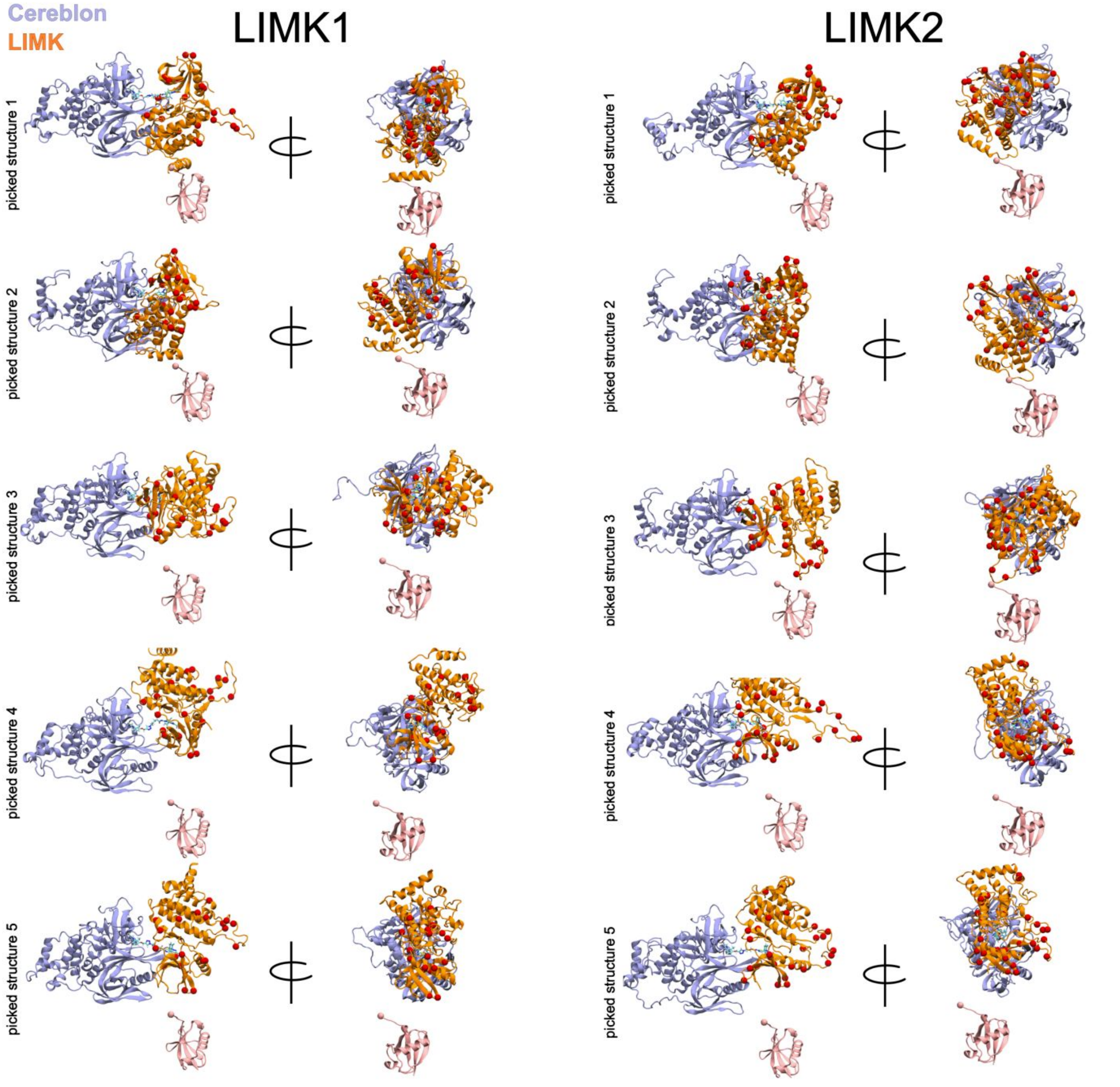

H

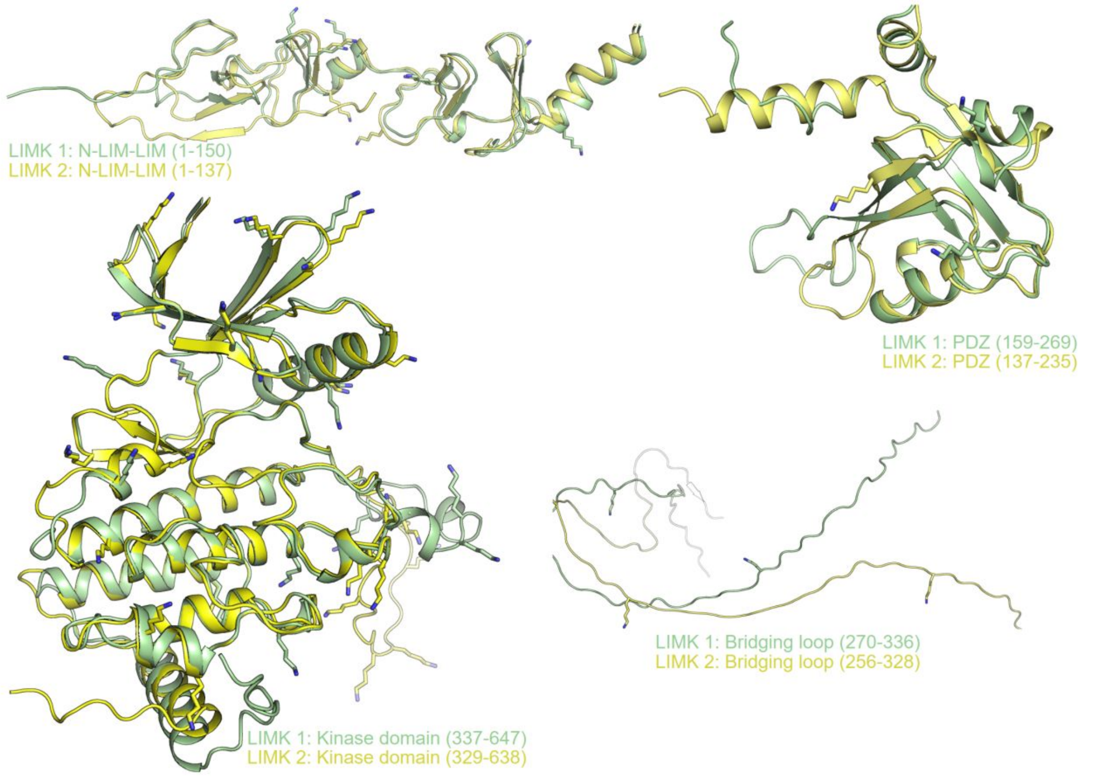

I

```
                      10        20        30        40        50        60
hLIMK1/1-647   1 MRLTLLCCTWREERMGEEGSELPVCASCGQRIYDGQ-YLQALNADWHADCFRCCDCSASLSHQYYEKDGQ  69
hLIMK2/1-638   1 -------------MSALAGEDVWRCPGCGDHIAPSQIWYRTVNETWHGSCFRCSECQDSLTNWYYEKDGK  57

                      80        90        100       110       120       130
hLIMK1/1-647  70 LFCKKDYWARYGESCHGCSEQITKGLVMVAGELKYHPECFICLTCGTFIGDGDTYTLVEHSKLYCGHCYY 139
hLIMK2/1-638  58 LYCPKDYWGKFGEFCHGCSLLMT-GPFMVAGEFKYHPECFACMSCKVIIEDGDAYALVQHATLYCGKCHN 126

                      150       160       170       180       190       200
hLIMK1/1-647 140 QTVVTPVIEQILPDSPGSHLPHTVTLVSIPASSHGKRGLSVSIDPPHGPPGCGTEHSHTVRVQGVDPGCM 209
hLIMK2/1-638 127 EVVLAPMFERLSTESVQEQLPYSVTLISMPATTEGRRGFSVSVES------ACSNYATTVQVKEVNRMHI 190

                      220       230       240       250       260       270
hLIMK1/1-647 210 SPDVKNSIHVGDRILEINGTPIRNVPLDEIDLLIQETSRLLQLTLEHDPHDT------LGHGLGPETS-- 271
hLIMK2/1-638 191 SPNNRNAIHPGDRILEINGTPVRTLRVEEVEDAISQTSQTLQLLIEHDPVSQRLDQLRLEARLAPHMQNA 260

                      290       300       310       320       330       340
hLIMK1/1-647 272 --PLSSPAYTPSGEAGSSARQKPVLRSCSIDRSPGAGSLGSPASQRKDLGRSESLRVVCR-PHRIFRPSD 338
hLIMK2/1-638 261 GHPHALSTLDTKENLEGTLRRRSLRRSNSISKSPGPSSPKEPLLFSRDISRSESLRCSSSYSQQIFRPCD 330

                      360       370       380       390       400       410
hLIMK1/1-647 339 LIHGEVLGKGCFGQAIKVTHRETGEVMVMKELIRFDEETQRTFLKEVKVMRCLEHPNVLKFIGVLYKDKR 408
hLIMK2/1-638 331 LIHGEVLGKGFFGQAIKVTHKATGKVMVMKELIRCDEETQKTFLTEVKVMRSLDHPNVLKFIGVLYKDKK 400

                      430       440       450       460       470       480
hLIMK1/1-647 409 LNFITEYIKGGTLRGIIKSMDSQYPWSQRVSFAKDIASGMAYLHSMNIIHRDLNSHNCLVRENKNVVVAD 478
hLIMK2/1-638 401 LNLLTEYIEGGTLKDFLRSMD-PFPWQQKVRFAKGIASGMAYLHSMCIIHRDLNSHNCLIKLDKTVVVAD 469

                      500       510       520       530       540       550
hLIMK1/1-647 479 FGLARLMVDEKTQP------EGLRSLKKPDRKKRYTVVGNPYWMAPEMINGRSYDEKVDVFSFGIVLCEI 542
hLIMK2/1-638 470 FGLSRLIVEERKRAPMEKATTKKRTLRKNDRKKRYTVVGNPYWMAPEMLNGKSYDETVDIFSFGIVLCEI 539

                      570       580       590       600       610       620
hLIMK1/1-647 543 IGRVNADPDYLPRTMDFGLNVRGFLDRYCPPNCPPSFFPITVRCCDLDPEKRPSFVKLEHWLETLRMHLA 612
hLIMK2/1-638 540 IGQVYADPDCLPRTLDFGLNVKLFWEKFVPTDCPPAFFPLAAICCRLEPESRPAFSKLEDSFEALSLYLG 609

                      640       650       660
hLIMK1/1-647 613 GH-LPLGPQLEQLDRGFWETYRRGESGLPAHPEVPD                                   647
hLIMK2/1-638 610 ELGIPLPAELEELDHTVSMQYGLTRDSPP-------                                   638
```

Figure S6. Structural Assessment of LIMK2 Isoform Specificity.
(A) Mass photometry analysis of full-length LIMK1. Mass distribution shows distinct peaks corresponding to monomeric and dimeric species. An exemplary ratiometric contrast image taken at 40 seconds is shown, highlighting individual particle landing events on the glass surface. Quantification confirms the presence of stable dimer formation alongside the monomer population.
(B) Atoms used for RMSD calculations to assess warhead rigidity within the protein binding pockets are indicated by orange boxes. Atoms used for plane and geometric centre calculations are circled in red.
(C) MD simulations (0–90 ns). Orientations of the LIMK1 and LIMK2 kinase domains relative to CRBN in the ternary complex, represented by the distance between the PROTAC warheads and their relative orientations. Initial starting structures are shown as red dots.
(D) MD simulations. Complex flexibilities and binding pose stabilities for LIMK1/2 kinase domain in ternary complex with CRBN. The Cα position RMSD of LIMK2 of each individual replica (aligned on CRBN Cα positions in the initial starting structure, and last frame) reveal relative stability after the initial reorientation phase. The RMSF values of CRBN, and LIMK2 reveal major flexibilities in loop regions (LIMKs) or helix connected via two larger loops (CRBN) while the remaining protein regions are less flexible. Individual replicas are shown in shallow blue lines. A running average of 10 ns was applied to the LIMK1/2 RMSD values.
(E) MD simulations. LIMK1 kinase domain and LIMK2 kinase domain orientations relative to CRBN in the ternary complex, represented by the distance of the PROTAC warheads and their relative orientations over the simulation time for each replica in blue (LIMK1: 30; LIMK2: 50). Populated clusters 1-5 are indicated with their respective values.
(F) Distribution of plane angles and distances in LIMK1/2 ternary complexes, used to select representative structures for alignment onto the static E3 ligase complex to evaluate lysine accessibility for ubiquitination. Selected structures correspond to the following angle–distance pairs: (i) 60° and 23.5 Å, (ii) 67° and 19.3 Å, (iii) 153° and 21.7 Å, (iv) 210° and 21.7 Å, and (v) 216° and 23.8 Å. Clusters are numbered as in Figure 6F.
(G) LIMK1 and LIMK2 ternary complex structures in MD simulations. Selected MD simulation structures were aligned to the CRBN-containing static Cullin-4A E3 ligase complex structure. Proteins are shown in surface representation: DDB1 (purple), Cullin-4A (blue), UBE2D2 (tan, transparent), NEDD8 (gray), and RBX1 (yellow). Ubiquitin (Ub) is shown in cartoon representation (pink). MD structures are shown as cartoons: CRBN (ice blue), LIMK1 or LIMK2 (orange). Cα atoms of lysine residues on LIMK1/LIMK2 are shown as red spheres; the Cα atom of the terminal ubiquitin glycine is shown as a pink sphere. Representative structures (1–5) from MD simulations of LIMK1 and LIMK2 ternary complexes are shown only with the Ub of the E3 ligase complex for visual inspection.
(H)LIMK1/2 individual domain and sequence alignment. Structural alignment of individual LIMK1 and LIMK2 domains was performed using PyMOL.[1] All lysine residues are shown as stick representations.
(I) Pairwise sequence alignment of LIMK1 and LIMK2. Alignments were generated using Jalview,[2] with lysine residues highlighted in blue.

EXPERIMENTAL SECTION

Chemical Synthesis: General Procedures

Starting materials were purchased from commercial suppliers and used without further purification. NMR spectra were recorded on a Bruker Avance (500 MHz, 400 MHz, 300 MHz, or 250 MHz), and chemical shifts (δ) are reported in ppm, using residual protic solvent as reference. Thin-layer chromatography was used to check the progress of the reaction and the purity of a substance. Ready-to-use ALUGRAM Xtra SIL

G/UV254 polyester sheets from Machery-Nagel were used for this purpose. Detection was achieved using UV light at the wavelengths λ = 254 nm and λ = 336 nm. The puriFlash® X420Plus system with a UV-VIS multi-wave detector (200 – 400 nm) from Interchim was used to perform column chromatography. Pre-packed silica gel cartridges were used as the stationary phase. In more detail, normal phase PF-SIHP silica columns with a particle size of 15, 30, or 50 µm and reversed phase RP-C18 columns. Technical-grade solvents were used as the mobile phase. The Agilent HPLC system 1260 Infinity II was used to evaluate the purity of the compounds. This system included a single quadrupole LC/MSD system InfinityLab (G6125B, ESI pos. 100-1000), a diode array detector 1260 DAD HS (G7177C), a flexible pump (G7104C), a multi-column thermostat (G7166A), a multisampler (G7167A), and a column compartment (G7117A). A Poroshell 120 EC-C18 column from Agilent (3.0 x 150 mm, 2.7 µm) served as the stationary phase. A gradient of H_2O (A) /ACN (B) with 0.1% formic acid was used as the mobile phase. UV detection was performed at 254 nm, 280 nm, and 310 nm. Two different gradient methods were used with a flow rate of 0.6 mL/min: Method 1: 0 min, 5% B – 2 min, 80% B – 5 min, 95% B – 7 min, 95% B. Method 2: 0 min, 5% B – 0.4 min 5% B – 8 min, 100% B – 10 min, 100% B. The chemicals purchased commercially were used without further purification. All final compounds were obtained with a purity of>95% if not otherwise mentioned. Mass spectrometry (ESI+/ESI-) was measured on a VG Platform II spectrometer from Fisons. HRMS was measured on a ThermoScientific MALDI LTQ Orbitrap XL or a Bruker micOTOF.

A) Synthesis of LIMKi3 Azide Handles:

Synthesis of 1-(2,4-Dimethoxybenzyl)thiourea (**3**)

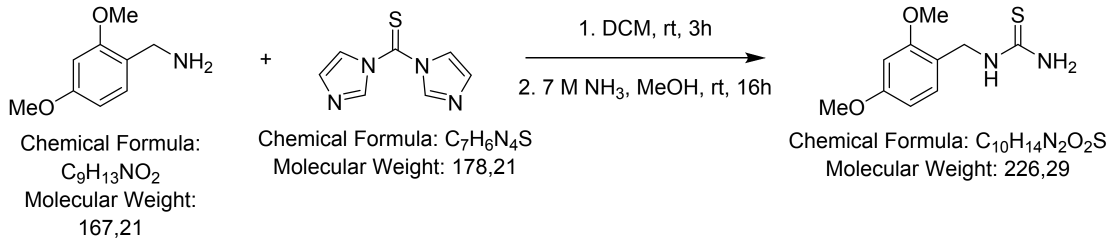

1,1'-thiocarbonyldiimidazole (3 g, 16,83 mmol, 1.5 eq.) was dissolved in 150 mL DCM, and 2,4-Dimethoxybenzylamine (1.69 mL, 11.22 mmol, 1 eq.) was dissolved in 75 mL DCM and slowly added to the solution over 3 h. The reaction was stirred for another hour, and 8.6 mL 7 M NH_3 in MeOH was added. The reaction mixture was stirred overnight. The next day, the precipitate was filtered off and washed with DCM, before removing all volatile compounds under reduced pressure. Yielding an orange/brown powder as the product.

Theoretical Yield: 1.42 g

Yield: 1.140 g ≡ 44%

^{1}H NMR (250 MHz, $(CD_3)_2SO$): δ = 7.66 (s, 1H), 7.12 (d, J_{3H-H} = 8.3 Hz, 1H), 6.98 (s, 1H), 6.55 (d, J_{4H-H} = 2.4 Hz, 1H), 6.49 (d, J_{3H-H} = 8.3, 2.4 Hz, 1H), 4.45 (s, 2H), 3.79 (s, 3H), 3.74 (s, 3H).
MS (ESI+): *m/z* = 227.0 [M+H]$^+$

Synthesis of N'-((2,4-Dimethoxybenzyl)carbamothioyl)-N,N-dimethylformamidin (**4**)

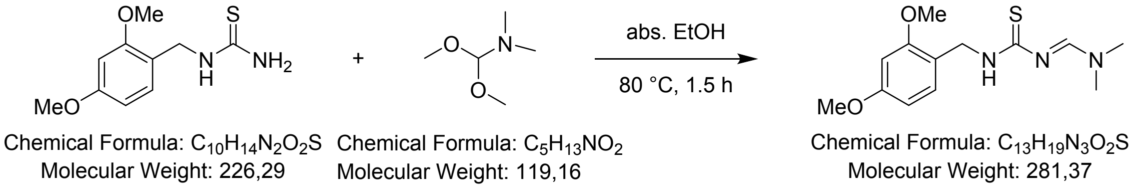

1-(2,4-Dimethoxybenzyl) thiourea (1.14 g, 5.04 mmol, 1.0 eq.) was added to 6 mL of abs. EtOH and stirred under an argon atmosphere. Thereafter, Dimethylformamid-dimethylacetal (1.08 mL, 8.06 mmol, 1.6 eq) was added to the solution with the unsolved reactant. The reaction was heated to 80 °C and stirred for 1.5 h, after which the precipitation had dissolved completely. After the solution was cooled down to room temperature, it was stored in a fridge overnight. The next day, a white precipitation had formed in the otherwise clear and golden solution. The precipitation was filtered over a frit and washed with EtOH. The white powder was dried in vacuo at 50 °C overnight.
Theoretical Yield: 1.42 g
Yield: 1.093 g ≡ 77%
^{1}H NMR (250 MHz, $(CD_3)_2SO$): Conformer 1: 8.76 (t, J_{3H-H} = 6.1 Hz, 1H), 8.71 (s, 1H), 7.02 (d, J_{3H-H} = 8.3, 1H), 6.52 (m, 1H), 6.49-6.41 (m, 1H), 4.63 (d, J_{3H-H} = 6.0 Hz, 2H), 3.78 (s, J_{4H-H} = 3.7 Hz, 3H), 3.73 (s, 3H), 3.13 (s, 3H), 3.00 (s, 3H).
Conformer 2: 8.76 (t, J_{3H-H} = 6.1 Hz, 1H), 8.66 (s, 1H), 7.03 (d, J_{3H-H} = 8.3, 1H), 6.52 (m, 1H), 6.49-6.41 (m, 1H), 4.63 (d, J_{3H-H} = 6.0 Hz, 2H), 3.78 (s, J_{4H-H} = 3.7 Hz, 3H), 3.73 (s, 3H), 3.13 (s, 3H), 2.98 (s, 3H).
MS (ESI+): *m/z* = 282.10 [M+H]$^+$

Synthesis of 1-(2-((2,4-Dimethoxybenzyl)amino)thiazol-5-yl)ethan-1-on (**5**)

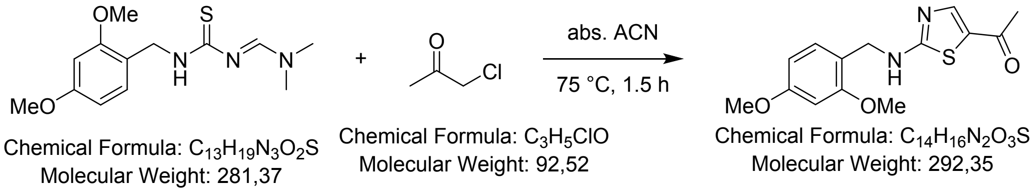

N'-((2,4-Dimethoxybenzyl)carbamothioyl)-N,N-dimethylformamidin (1.09 g, 3.87 mmol, 1.0 eq.) was dissolved in 15 mL of abs. ACN and chloroacetone (0.374 mL, 4.65 mmol, 1.2 eq.) were added to the

solution. The mixture was refluxed under argon at 75 °C for 1.5 h. After the solution cooled down to rt 12 mL H_2O and 3.5 mL saturated $NaHCO_3$ solution were added. This caused precipitation of the product, which was filtered off over a frit and washed three times with 25 mL Et_2O:pentane (1:4). The product was dried at 50 °C in vacuo.

Theoretical Yield: 1.13 g

Yield: 920 mg ≡ 81%

^{1}H NMR (250 MHz, $(CD_3)_2SO$): δ = 8.83 (s, 1H), 7.97 (s, 1H), 7.15 (d, $J_{3H\text{-}H}$ = 8.3 Hz, 1H), 6.57 (d, $J_{4H\text{-}H}$ = 2.4 Hz, 1H), 6.48 (dd, $J_{3H\text{-}H}$ = 8.3, $J_{3H\text{-}H}$ = 2.4 Hz, 1H), 4.37 (d, $J_{3H\text{-}H}$ = 5.3 Hz, 2H), 3.80 (s, 3H), 3.74 (s, 3H), 2.34 (s, 3H) ppm.

MS (ESI+): *m/z* = 293.05 $[M+H]^+$

Synthesis of (Z)-1-(2-(2,4-dimethoxybenzylamino)thiazol-5-yl)-4,4-difluoro-3-hydroxybut-2-en-1-one (**6**)

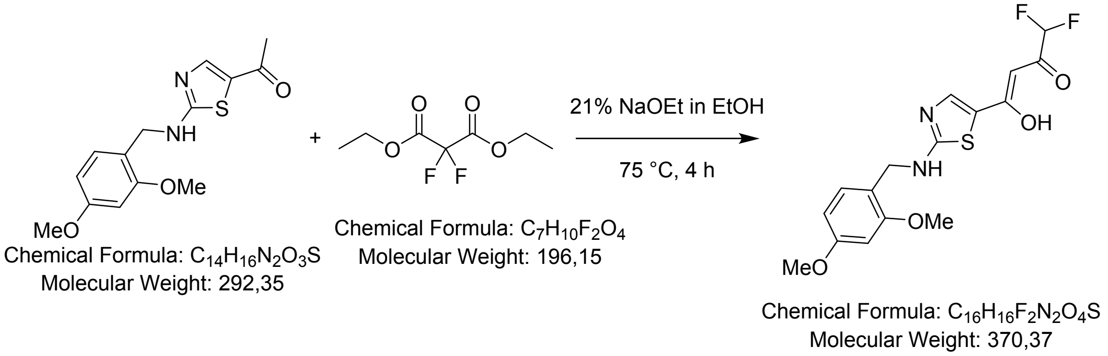

1-(2-((2,4-Dimethoxybenzyl)amino)thiazol-5-yl)ethan-1-on (3.00 g, 10.3 mmol, 1.0 eq.) and Diethyldifluoromalonate (5.66 mL, 41.05 mmol, 4.0 eq.) were dissolved in 15.0 mL NaOEt (21% mass) in EtOH under argon atmosphere. The solution was stirred under reflux for 16 h. After the solution was cooled down to rt, it was transferred to a conical flask and diluted with 20 mL EtOH and 45 mL H_2O. The pH of the mixture was adjusted to 5-6 through the addition of 3 mL glacial acetic acid. This occurring orange precipitation was separated via filtration and washed with 50 mL H_2O:MeOH (2:1) and 50 mL hexane:Et_2O (4:1). The remaining powder was dried at 50 °C in vacuo.

Theoretical Yield: 3.80 g

Yield: 2.59 g ≡ 68%

^{1}H NMR (250 MHz, $(CD_3)_2SO$): δ = 9.25 (s, 1H), 8.33 (s, 1H), 7.19 (d, $J_{3H\text{-}H}$ = 8.3 Hz, 1H), 6.58 (d, $J_{4H\text{-}H}$ = 1.9 Hz, 1H), 6.51 (d, $J_{4H\text{-}H}$ = 2.4 Hz, 1H), 6.48 (t, $J_{2H\text{-}F}$ = 53.4 Hz, 1H), 4.42 (d, $J_{3H\text{-}H}$ = 5.6 Hz, 2H), 3.80 (s, 3H), 3.75 (s, 3H) ppm.

MS (ESI+): *m/z* = 371.05 $[M+H]^+$

Synthesis of N-(2,4-Dimethoxybenzyl)-5-(1-(2,6-dichlorophenyl)-3- (difluoromethyl)-1H-pyrazol-5-yl)thiazol-2-amine (7)

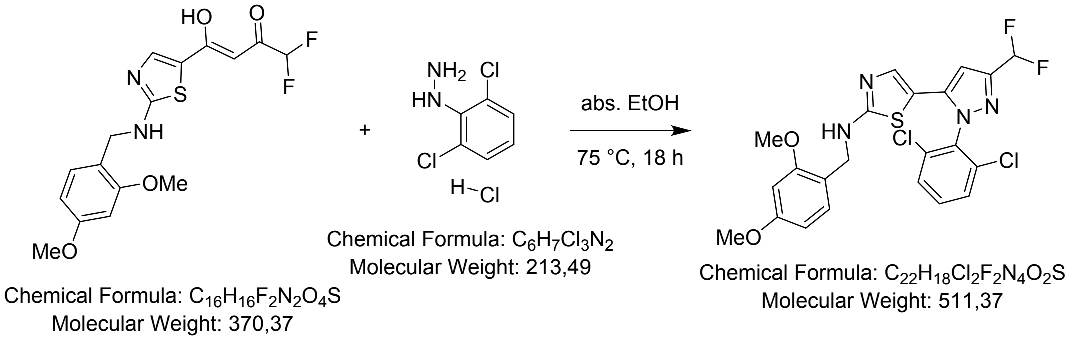

(*Z*)-1-(2-(2,4-dimethoxybenzylamino)thiazol-5-yl)-4,4-difluoro-3-hydroxybut-2-en-1-one (300 mg, 810 µmol, 1.0 eq.) and 2,6-dichlorophenylhydrazine hydrochloride (207.51 mg, 972 µmol, 1.2 eq.) were dissolved in 6 mL abs. EtOH. The reaction mixture was refluxed at 75 °C for 24 h under argon. To stop the reaction, 3 mL of water and 1 mL of saturated $NaHCO_3$ solution were added, which caused precipitation of a yellow powder. The resulting slurry was filtered off over a frit and was washed with MeOH:H_2O (1:2), as well as Et_2O:hexane (1:4). The product was dried at 50 °C in vacuo.

Theoretical Yield: 414.21 mg

Yield: 270 mg ≡ 65%

^{1}H NMR (250 MHz, $(CD_3)_2SO$): δ = 8.13 (t, $J_{3H\text{-}H}$ = 5.6 Hz, 1H), 7.86-7.61 (m, 3H), 7.21 (s, 1H), 7.08 (t, $J_{2H\text{-}F}$ = 54.5 Hz, 1H), 7.07 (s, 1H), 7.01 (s, 1H), 6.53 (d, $J_{4H\text{-}H}$ = 2.4 Hz, 1H), 6.45 (dd, $J_{3H\text{-}H}$ = 8.3, $J_{4H\text{-}H}$ = 2.4 Hz, 1H), 4.25 (d, $J_{3H\text{-}H}$ = 5.6 Hz, 2H), 3.76 (s, 3H), 3.73 (s, 3H) ppm.

MS (ESI–): *m/z* = 511.05 [M–H]$^-$

Synthesis of 4-(2-amino)thiazol-5-yl)-1,1-difluoro-4-hydroxybut-3-en-2-on (**8**)

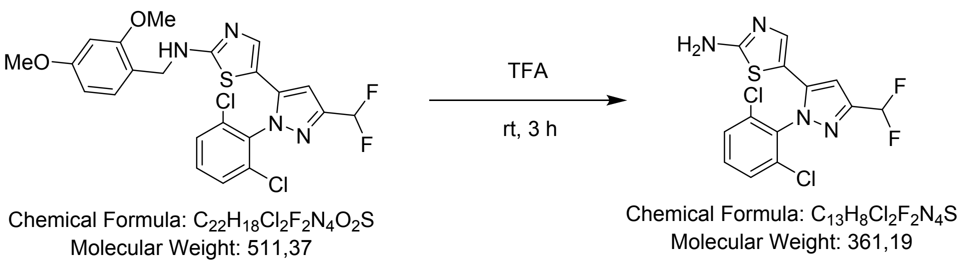

4-(2-((2,4-Dimethoxybenzyl)amino)thiazol-5-yl)-1,1-difluoro-4-hydroxybut-3-en-2-on (350 mg, 684,44 µmol, 1.0 eq.) was solved in TFA (6 mL, 80,77 mmol, 118 eq.) and stirred at rt under argon for 3 h. To the violet reaction solution, 20 mL of water was added, and its pH was adjusted to 7 by adding saturated

$NaHCO_3$ solution. While neutralizing the solution lost its colour, and a white precipitate formed in the now clear solution. Also, a black residue formed on top of the solution. The solids were filtered off and washed with water. To obtain the product purification via flash column chromatography was performed with hexane:EE (1:1→ 0:1).

Theoretical Yield: 247.21 mg

Yield: 200 mg ≡ 81%

^{1}H NMR (250 MHz, $(CD_3)_2SO$): δ = 7.82 – 7.66 (m, 3H), 7.46 (s, 2H) 7.24 (s, 1H), 7.07 (t, $J_{2H\text{-}F}$ = 54.3 Hz, 1H), 7.05 (s, 1H) ppm.

MS (ESI–): *m/z* = 360.95 [M–H]$^-$

TLC: (n-hexane : EE) (1:1) R_f = 0.36

Synthesis of 2-chloro-N-(5-(1-(2,6-dichlorophenyl)-3-(difluoromethyl)-1H-pyrazol-5-yl)thiazol-2-yl)acetamide (**9a**)

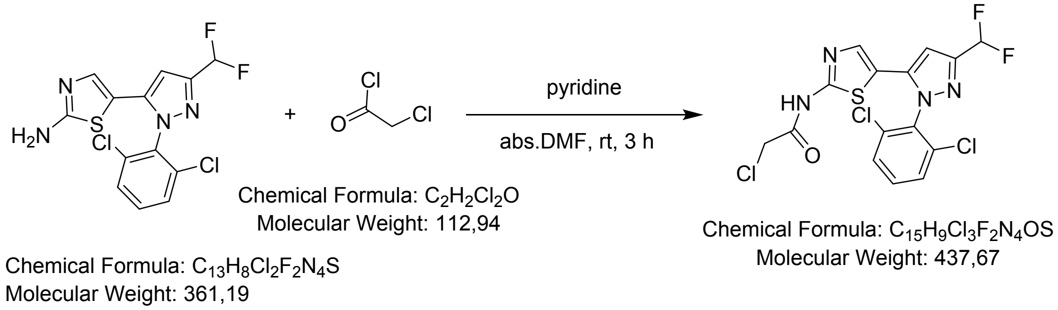

4-(2-amino)thiazol-5-yl)-1,1-difluoro-4-hydroxybut-3-en-2-on (250.0 mg, 692.2 µmol, 1.0 eq.) and chloroacetyl chloride (171.1 µL, 1.73 mmol, 2.5 eq.) were dissolved in abs. DMF and pyridine (139.4 µL, 1.73 mmol, 2.5 eq.) were added. The solution was stirred at rt for 3 h and was quenched with 15 mL H_2O after the conversion was monitored via HPLC-MS (ESI). The product was first extracted with 20 mL EE three times, then dried over Na_2SO_4 and subsequently purified via reverse phase (RP)-flash column chromatography (H_2O: ACN) (9:1 → 0:1).

Theoretical Yield: 303 mg

Yield: 255 mg ≡ 84%

^{1}H NMR (500 MHz, $(CD_3)_2SO$): δ = 12.70 (s, 1H), 7.88 – 7.69 (m, 5H), 7.28 (s, 1H), 7.14 (t, $J_{2H\text{-}F}$ = 54.2 Hz, 1H), 4.35 (s, 2H) ppm.

MS (ESI–): *m/z* = 436.95 [M–H]$^-$

TLC: (n-hexane : EE) (1:1) R_f = 0.30

Synthesis of 5-chloro-N-(5-(1-(2,6-dichlorophenyl)-3-(difluoromethyl)-1H-pyrazol-5-yl)thiazol-2-yl)pentanamide (**9b**)

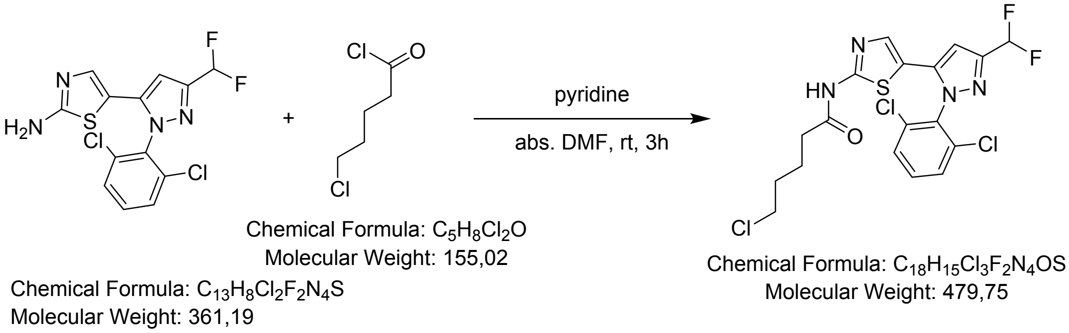

4-(2-amino)thiazol-5-yl)-1,1-difluoro-4-hydroxybut-3-en-2-on (250.0 mg, 692.2 µmol, 1.0 eq.) and 5-chlorovaleryl chloride (97.9 µL, 761.4 µmol, 1.1 eq.) were solved in abs. DMF and pyridine (61.3 µL, 761.4 µmol, 1.1 eq.) were added. The solution was stirred at rt for 3 h and was quenched with 15 mL H_2O with 0.1% formic acid (FA). Celite was added to the mixture, and the solvent was evaporated under reduced pressure. The purification was accomplished via RP-flash column chromatography (H_2O:ACN) (9:1 → 0:1). The product yielded was obtained as a brown oil.

Theoretical Yield: 332 mg

Yield: 268 mg ≡ 81%

^{1}H NMR (250 MHz, $(CD_3)_2SO$): δ =12.31 (s, 1H), 7.96 – 7.69 (m, 3H), 7.67 (s, 1H), 7.23 (s, 1H), 7.12 (t, J_{2H-F} = 54.2 Hz, 1H), 3.92 – 3.56(m, 2H), 2.43 (t, J_{3H-H} = 7.3 Hz, 2H), 1.86 – 1.41 (m, 2H) ppm.

MS (ESI+): *m/z* = 481.07 $[M+H]^+$

TLC: (ACN : H_2O) (1:1) R_f = 0.07

Synthesis of 6-bromo-N-(5-(1-(2,6-dichlorophenyl)-3-(difluoromethyl)-1H-pyrazol-5-yl)thiazol-2-yl)hexanamide (**9c**)

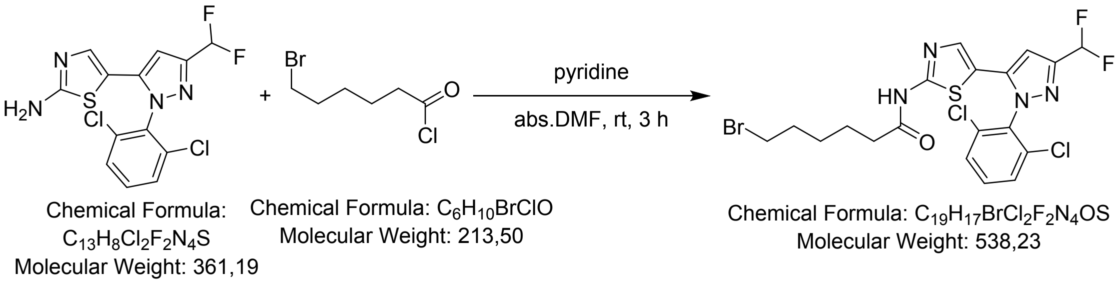

4-(2-amino)thiazol-5-yl)-1,1-difluoro-4-hydroxybut-3-en-2-on (250.0 mg, 692.2 µmol, 1.0 eq.) and 6-chlorohexanyl bromide (264.8 µL, 1.73 mmol, 2.5 eq.) were solved in abs. DMF and pyridine (640 µL, 1.73 mmol, 2.5 eq.) were added. The solution was stirred at rt for 3 h and was quenched with 15 mL H_2O. Then

the product was extracted three times with 15 mL EE and dried over Na_2SO_4. Celite was added to the mixture, and the solvent was evaporated under reduced pressure. The purification was accomplished via RP-flash column chromatography (H_2O:ACN) (9:1 → 0:1). The product yielded was obtained as a brown oil.

Theoretical Yield: 372.54 mg

Yield: 294 mg ≡ 79 %

^{1}H NMR (250 MHz, $(CD_3)_2SO$): δ = 12.29 (s, 1H), 7.98 – 7.70 (m, 3H), 7.67 (s, 1H), 7.23 (s, 1H), 7.12 (t, $J_{2H\text{-}F}$ = 54.2 Hz, 1H), 2.40 (t, $J_{3H\text{-}H}$ = 7.3 Hz, 2H), 2.21 (t, $J_{3H\text{-}H}$ = 7.1 Hz, 2H), 1.79 (m, 4H), 1.62 – 1.51 (m, 2H) ppm.

MS (ESI+): *m/z* = 538.95 $[M+H]^+$

Synthesis of 2-azido-N-(5-(1-(2,6-dichlorophenyl)-3-(difluoromethyl)-1H-pyrazol-5-yl)thiazol-2-yl)acetamide (**10a**)

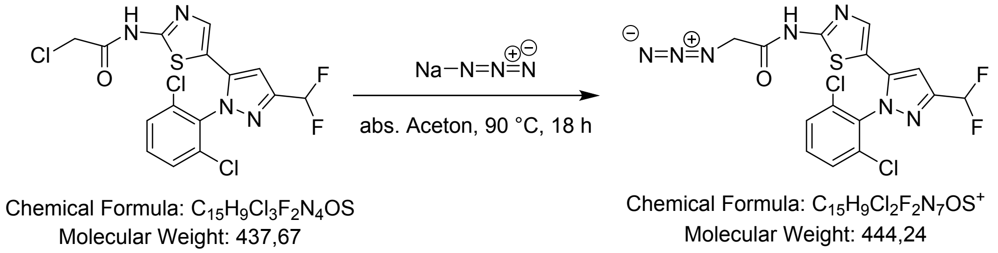

2-chloro-*N*-(5-(1-(2,6-dichlorophenyl)-3-(difluoromethyl)-1*H*-pyrazol-5-yl)thiazol-2-yl)acetamide (250 mg, 571.21 µmol, 1.0 eq.) was dissolved in 12 mL abs. acetone and sodium azide (74 mg, 1.14 mmol, 2.0 eq.) were added. The solution was stirred under reflux for 18 h. Thereafter, the mixture was extracted with DCM and washed with H_2O three times each. The united organic phases were dried over Na_2SO_4 , and the solvent was evaporated under reduced pressure, resulting in a brown resin.

Theoretical Yield: 430.37 mg

Yield: 343 mg ≡ 80%

^{1}H NMR (250 MHz, $(CD_3)_2SO$): δ = 12.57 (s, 1H), 7.87 – 7.74 (m, 3H), 7.71 (s, 1H), 7.27 (s, 1H), 7.14 (t, $J_{2H\text{-}F}$ = 54.2 Hz, 1H), 4.15 (s, 2H) ppm.

MS (ESI–): *m/z* = 443.95 $[M–H]^–$

TLC: (n-hexane : EE) (1:1) R_f = 0.36

Synthesis of 5-azido-N-(5-(1-(2,6-dichlorophenyl)-3-(difluoromethyl)-1H-pyrazol-5-yl)thiazol-2-yl)pentanamide (**10b**)

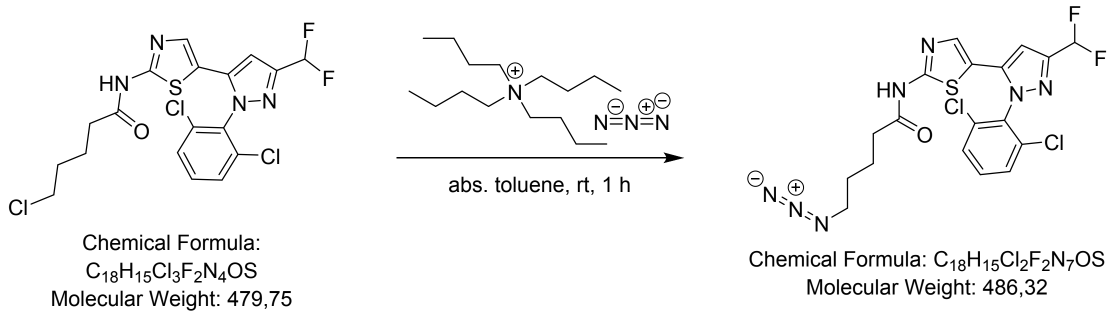

A solution of 5-chloro-*N*-(5-(1-(2,6-dichlorophenyl)-3-(difluoromethyl)-1*H*-pyrazol-5-yl)thiazol-2-yl)pentanamide (268 mg, 559 µmol, 1.0 eq.) and tetrabutylammonium azide (477 mg, 1.68 mmol, 3.0 eq.) in 15 mL toluene was stirred and heated to 100 °C for one hour. The product was obtained by RP-flash column chromatography (H_2O:ACN) (9:1 → 0:1) after adding celite to the solution and removing all volatile substances were removed under reduced pressure. The product was characterised as a white powder.

Theoretical Yield: 272 mg

Yield: 203 mg ≡ 75%

MS (ESI–): *m/z* = 486.05 $[M–H]^-$

TLC: (ACN : H_2O) (1:1) R_f = 0.30

Synthesis of 6-azido-N-(5-(1-(2,6-dichlorophenyl)-3-(difluoromethyl)-1H-pyrazol-5-yl)thiazol-2-yl)hexanamide (**10c**)

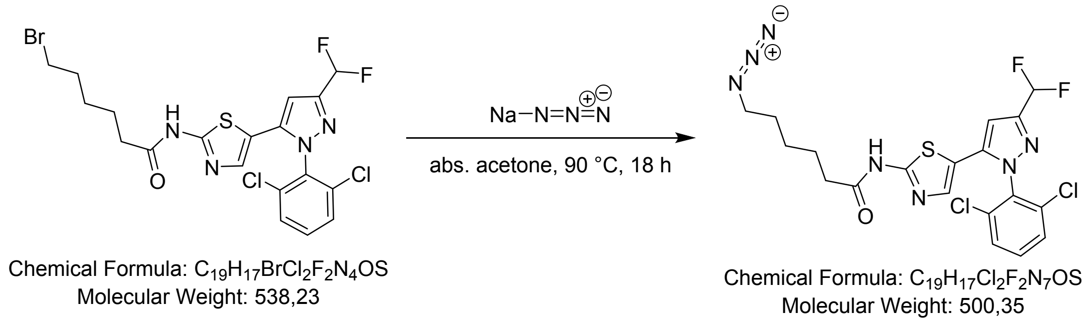

6-azido-*N*-(5-(1-(2,6-dichlorophenyl)-3-(difluoromethyl)-1*H*-pyrazol-5-yl)thiazol-2-yl)acetamide (350 mg, 650 µmol, 1.0 eq.) was added to a solution of sodium azide (85 mg, 1.30 mmol, 2.0 eq.) in 12 mL abs. acetone and refluxed at 90 °C for seven hours. Thereafter, an HPLC-MS (ESI) measurement showed that only 50% of the educt was converted into product. Hence, sodium azide (85 mg, 1.30 mmol, 2.0 eq.) was added once more to the reaction. After another six hours of refluxing, the conversion was still not complete, sodium azide (85 mg, 1.30 mmol, 2.0 eq.) was added again, as well as potassium iodide (10 mg, 60 µmol, 0.1 eq.). After another five hours of refluxing, the residue was absorbed in 20 mL EE and washed

three times with 15 mL H_2O. The resulting product was isolated as a white powder by evaporating the volatile components of the mixture under reduced pressure, after drying the organic phase over Na_2SO_4.

Theoretical Yield: 325 mg

Yield: 202 mg ≡ 62%

^{1}H NMR (250 MHz, $(CD_3)_2SO$): δ = 12.29 (s, 1H), 7.91 – 7.70 (m, 3H), 7.67 (s, 1H), 7.23 (s, 1H), 7.12 (t, $J_{2H\text{-}F}$ = 54.2 Hz, 1H), 3.31 (t, $J_{3H\text{-}H}$ = 6.8 Hz, 1H), 2.40 (t, $J_{3H\text{-}H}$ = 7.3 Hz, 2H), 1.89 – 1.18 (m, 6H) ppm.

MS (ESI–): *m/z* = 500.00 $[M–H]^-$

TLC: (DCM : EtOH) (1:1) R_f = 0.79

B) Synthesis of CRBN and VHL Alkyne Handles

CRBN (4-OH-Thal):

Synthesis of 2-(2,6-dioxopiperidin-3-yl)-4-hydroxyisoindoline-1,3-dione (**13**)

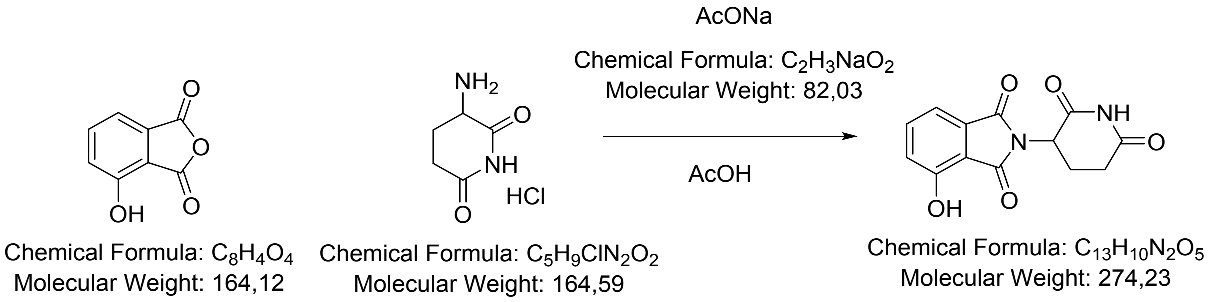

11 **12** **13**

A mixture of **11** (1 g, 6.09 mmol), 1**2** (1 g, 6.09 mmol), and NaOAc (1 g, 12.18 mmol) in HOAc (12 mL) was stirred at 110 °C for 12 h. The solvent was evaporated, water (5 mL) was added, and the resulting solution was sonicated for 1 min. The solid was filtered and concentrated to give compound 13as a black solid, which was used in subsequent steps without further purification.

Theoretical Yield: 1.666 g

Yield: 1.497 g ≡ 90 %

LC-MS (ESI+): *m/z* = 275.00 $[M+H]^+$

HPLC: t_R = 4.083, purity ≥ 95% (DAD1 C, Sig=320,150).

Synthesis of 2-(2,6-dioxopiperidin-3-yl)-4-(prop-2-yn-1-yloxy)isoindoline-1,3-dione (**14**)

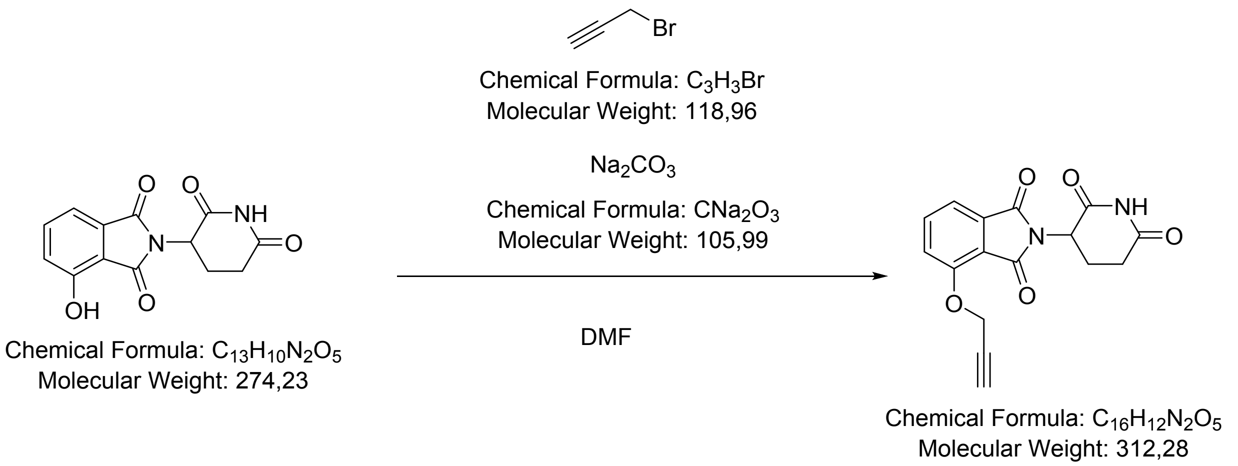

13 **14**

Intermediate 13 (500 mg, 1.82 mmol), propargyl bromide (304 mg, 284 µL, 2.55 mmol, 80wt.% in toluene), and Na_2CO_3 (290 mg, 2.73 mmol) in DMF (4 mL) were stirred at 60 °C for 3 h. The reaction was quenched by the addition of a sat. solution of NH_4Cl. The solvent was evaporated, water (20 mL) was added, and extracted with EtOAc (3 x 50 mL). The combined organic layers were washed with brine (100 mL), dried over Na2SO4, filtered, and concentrated. The crude residue was purified by reverse-phase column chromatography (RP-18, H2O/ACN) to afford the title compound as a pale white solid.

Theoretical Yield: 0.569 g

Yield: 0.320 g ≡ 56 %

^{1}H NMR (500 MHz, $(CD_3)_2SO$): δ = 11.10 (s, 1H), 7.86 (dd, *J* = 8.5, 7.3 Hz, 1H), 7.57 (d, *J* = 8.5 Hz, 1H), 7.51 (d, *J* = 7.2 Hz, 1H), 5.13 – 5.07 (m, 3H), 3.69 (t, *J* = 2.4 Hz, 1H), 2.89 (ddd, *J* = 17.1, 13.9, 5.4 Hz, 1H), 2.62 – 2.56 (m, 1H), 2.56 – 2.51 (m, 1H), 2.04 (dtd, *J* = 12.9, 5.3, 2.2 Hz, 1H).

^{13}C NMR (126 MHz, $(CD_3)_2SO$): δ = 172.76, 169.88, 166.68, 165.15, 154.27, 136.83, 133.34, 120.23, 116.83, 116.01, 79.41, 78.24, 56.44, 48.79, 30.92, 21.93.

LC-MS (ESI+): *m/z* = 313.05 $[M+H]^+$

HPLC: t_R = 4.693, purity ≥ 95% (DAD1 C, Sig=320,150).

CRBN (PD):

Synthesis of 1-(3-hydroxy-2-methylphenyl)dihydropyrimidine-2,4(1H,3H)-dione (**16**)

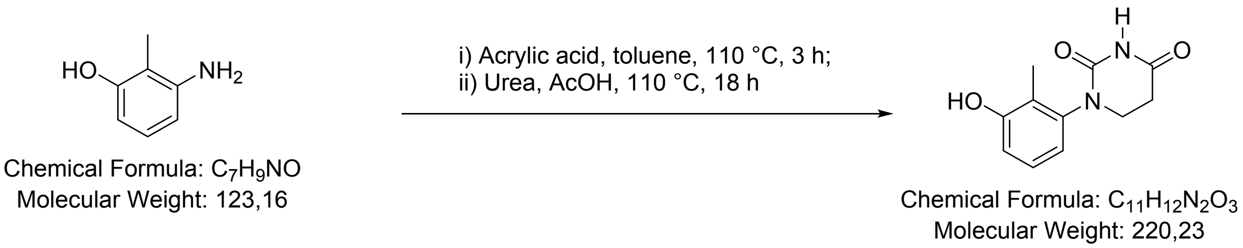

15 **16**

3-amino-2-methylphenol (**15**, 4.00 g, 32.5 mmol) and acrylic acid (3.34 mL, 48.7 mmol) were dissolved in dry toluene (20 mL) and stirred at 110 °C for 4 h. The solvent was removed under reduced pressure, and the crude reaction mixture was diluted with acetic acid (25 mL). Urea (5.85 g, 97.4 mmol) was added and stirred at 100 °C for 18 h. The reaction mixture was cooled in an ice bath (0 °C) and diluted with water, filtered, and the precipitate was washed with water (20 mL) and n-hexane (20 mL). The precipitate was dried under reduced pressure to give the title compound as a colourless solid (6.61 g, 92%).

Theoretical Yield: 7.153 g

Yield: 6.61 g ≡ 92 %

^{1}H NMR (400 MHz, $(CD_3)_2SO$): 10.27 (s, 1H), 9.48 (s, 1H), 7.01 (t, J = 7.8 Hz, 1H), 6.77 (dd, J = 8.1, 1.2 Hz, 1H), 6.70 (dd, J = 7.9, 1.2 Hz, 1H), 3.80 – 3.63 (m, 1H), 3.53 – 3.40 (m, 1H), 2.87 – 2.58 (m, 2H), 1.96 (s, 3H).

LC-MS (ESI+): *m/z* = 221.05 $[M+H]^+$

Synthesis of 1-(2-methyl-3-(prop-2-yn-1-yloxy)phenyl)dihydropyrimidine-2,4(1H,3H)-dione (**17**)

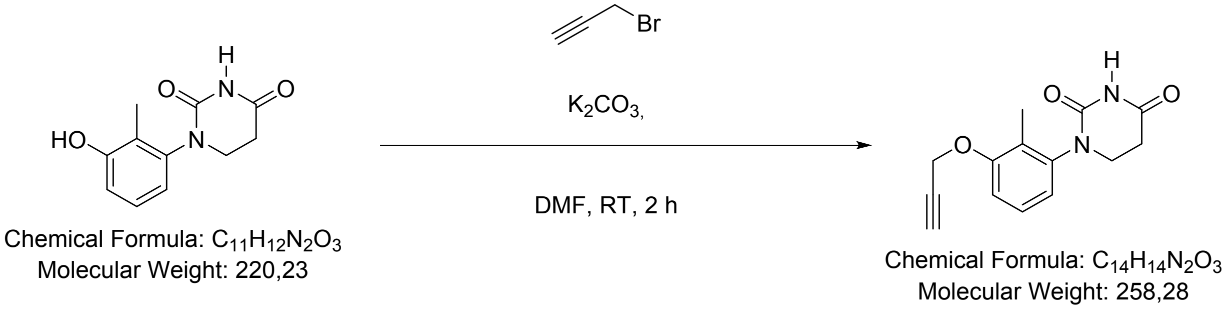

16 **17**

1-(3-hydroxy-2-methylphenyl)dihydropyrimidine-2,4(1H,3H)-dione (500 mg, 2.27 mmol), potassium carbonate (941 mg, 6.81 mmol), and Propargyl bromide (252 µl, 2.27 mmol) were dissolved in dry DMF (4 mL), and the reaction mixture was stirred at rt for 2 h. The solution was diluted with saturated aqueous

NH4Cl (10 mL), and the aqueous phase was extracted with EtOAc (4 x 10 mL). The combined organic phase was dried over MgSO4, filtered, and concentrated under reduced pressure. The crude product was purified by flash chromatography using acetonitrile/water as an eluent to obtain the title compound as a colourless solid (125 mg, 21%).

Theoretical Yield: 0.586 g

Yield: 0.13 g ≡ 21 %

^{1}H NMR (250 MHz, $(CD_3)_2SO$): 10.32 (s, 1H), 7.22 (t, J = 8.1 Hz, 1H), 7.01 (d, J = 8.3 Hz, 1H), 6.92 (d, J = 7.8 Hz, 1H), 4.84 (d, J = 2.4 Hz, 2H), 4.03 – 3.64 (m, 1H), 3.65 – 3.48 (m, 1H), 3.53 – 3.41 (m, 1H), 2.88 – 2.59 (m, 2H), 2.01 (s, 3H).

LC-MS (ESI+): *m/z* = 259.10 $[M+H]^+$

Synthesis of 3-methyl-1-(2-methyl-3-(prop-2-yn-1-yloxy)phenyl)dihydropyrimidine-2,4(1H,3H)-dione (**18**)

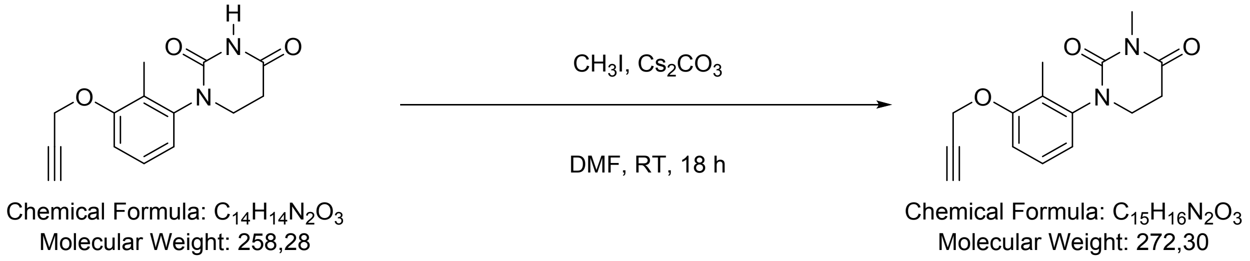

17 **18**

1-(2-methyl-3-(prop-2-yn-1-yloxy)phenyl)dihydropyrimidine-2,4(1H,3H)-dione (145 mg, 0.562 mmol), caesium carbonate (365 mg, 1.12 mmol) and iodomethane (105 µL, 1.68 mmol) were dissolved in dry DMF (2 mL) and the reaction mixture was stirred at rt for 18 h. The solution was diluted with saturated aqueous NH4Cl (10 mL), and the aqueous phase was extracted with EtOAc (4 x 10 mL). The combined organic phase was dried over MgSO4, filtered, and concentrated under reduced pressure. The crude product was purified by flash chromatography using acetonitrile/water as an eluent to obtain the title compound as a colourless solid (111 mg, 73%).

Theoretical Yield: 0.153 g

Yield: 0.11 g ≡ 73 %

^{1}H NMR (500 MHz, $(CD_3)_2SO$): δ = 7.22 (t, 1H), 7.02 (dd, J = 8.4, 1.1 Hz, 1H), 6.92 (dd, J = 8.0, 1.1 Hz, 1H), 4.85 (d, J = 2.5 Hz, 2H), 3.78 – 3.67 (m, 1H), 3.57 (t, J = 2.4 Hz, 1H), 3.53 – 3.43 (m, 1H), 3.05 (s, 3H), 2.96 – 2.75 (m, 2H), 2.01 (s, 3H).

^{13}C NMR (126 MHz, $(CD_3)_2SO$): δ = 169.76, 155.78, 152.27, 142.29, 126.59, 124.33, 119.78, 111.11, 79.34, 78.23, 55.94, 43.50, 31.34, 27.19, 10.72.

LC-MS (ESI+): *m/z* = 273.10 $[M+H]^+$

VHL (VH032):

Synthesis of (2S,4R)-1-((S)-3,3-dimethyl-2-propiolamidobutanoyl)-4-hydroxy-N-(4-(2-methylthiazol-5-yl)benzyl)pyrrolidine-2-carboxamide (**20a**)

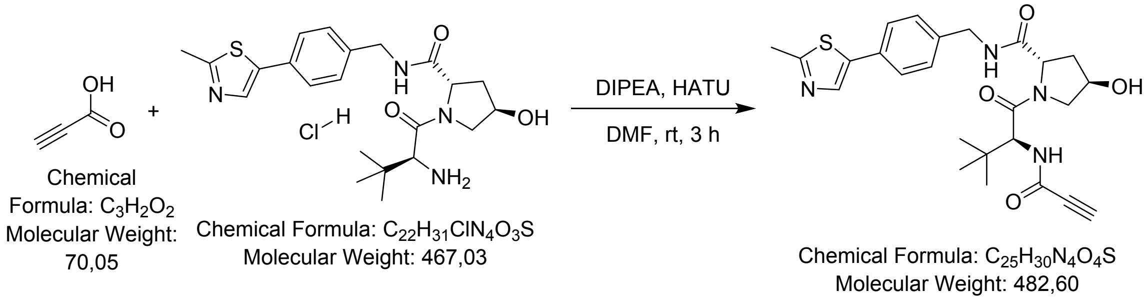

Prop-2-ynoic acid (26.5 µL, 428 µmol, 1.0 eq.) and VHL hydrochloride (200 mg, 428 µmol, 1.0 eq.) were added to a solution of 8 mL abs. DMF with DIPEA (400 µL, 2.29 mmol, 5.3 eq.) under argon. After the solution was stirred at rt for five minutes *O*-(7-Azabenzotriazol-1-yl)-*N*, *N*,*N′*,*N′*-tetramethyluronium-hexafluorphosphat (HATU) (407 mg, 1.07 mmol, 2.5 eq.) was added, and the solution was stirred for another three hours. The formation of the product was confirmed by HPLC-MS (ESI) and TLC. The reaction was quenched by the addition of 25 mL H_2O and extracted three times with 25 mL of EE. The united organic phases were washed with brine and dried over Na_2SO_4. Finally, the product was purified by flash column chromatography (DCM:EtOH) (9:1 → 0:1) and isolated as a white, powdery residue.

Theoretical Yield: 206 mg

Yield: 116 mg ≡ 56%

^{1}H NMR (250 MHz, $(CD_3)_2SO$): δ = 8.98 (s, 1H), 8.84 (d, J_{3H-H} = 9.1 Hz, 1H), 8.58 (t, J_{3H-H} = 6.0 Hz, 1H), 7.46 – 7.32 (m, 4H), 5.14 (d, J_{4H-H} = 3.6 Hz, 1H), 4.53 (d, J_{3H-H} = 9.1 Hz, 1H), 4.49 – 4.21 (m, 4H), 4.17 (s, 1H), 3.80 – 3.50 (m, 2H), 2.44 (s, 3H), 2.14 – 1.82 (m, 2H), 0.95 (s, 9H) ppm.

MS (ESI+): *m/z* = 483.15 $[M+H]^+$

TLC: (ACN : H_2O) (1:1) R_f = 0.54

Synthesis of (2S,4R)-1-((S)-2-(pent-4-ynamido)-3,3-dimethylbutanoyl)-4-hydroxy-N-(4-(2-methylthiazol-5-yl)benzyl)pyrrolidine-2-carboxamide (**20b**)

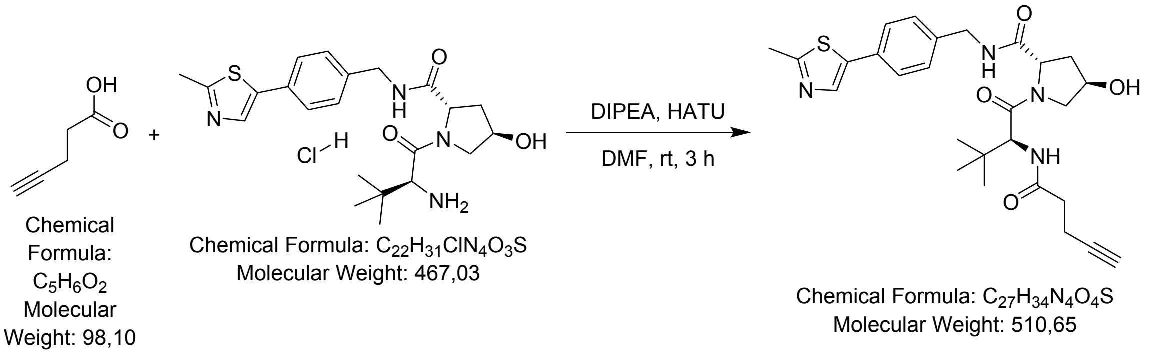

Pent-5-ynoic acid (63 mg, 642 µmol, 1.0 eq.) and VHL hydrochloride (300 mg, 642 µmol, 1.0 eq.) were added to a solution of 10 mL abs. DMF with DIPEA (600 µL, 3.43 mmol, 5.3 eq.) and HATU (611 mg, 1.61 mmol, 2.5 eq.) under argon. The solution was stirred at rt for three hours and monitored via TLC, whereafter the reaction was quenched by the addition of 25 mL of H_2O. The solution was extracted three times with 25 mL EE, and the united organic phases were dried over Na_2SO_4. The workup was completed via flash column chromatography (DCM:EtOH) (98:2 → 0:1). The product was obtained as a white powdery residue.

Theoretical Yield: 328 mg

Yield: 304 mg ≡ 93%

^{1}H NMR (250 MHz, $(CD_3)_2SO$): 8.98 (s, 1H), 8.56 (t, $J_{3H\text{-}H}$ = 6.0 Hz, 1H), 7.98 (d, $J_{3H\text{-}H}$ = 9.3 Hz, 1H), 7.52 – 7.32 (m, 4H), 5.13 (d, $J_{4H\text{-}H}$ = 3.5 Hz, 1H), 4.55 (d, $J_{3H\text{-}H}$ = 9.3 Hz, 1H), 4.48 – 4.29 (m, 3H), 4.21 (dd, $J_{2H\text{-}H}$ = 15.9, $J_{3H\text{-}H}$ = 5.5 Hz, 1H), 3.75 – 3.55 (m, 2H), 3.03 (s, 2H), 2.73(t, $J_{4H\text{-}H}$ = 2.4 Hz, 1H), 2.44 (s, 3H), 2.41 – 2.29 (m, 2H), 2.16 – 1.82 (m, 2H), 0.94 (s, 9H) ppm.

MS (ESI+): *m/z* = 511.20 $[M+H]^+$

TLC: (ACN : H_2O) (1:1) R_f = 0.50

Synthesis of (2S,4R)-1-((S)-2-(hex-5-ynamido)-3,3-dimethylbutanoyl)-4-hydroxy-N-(4-(2-methylthiazol-5-yl)benzyl)pyrrolidine-2-carboxamide (**20c**)

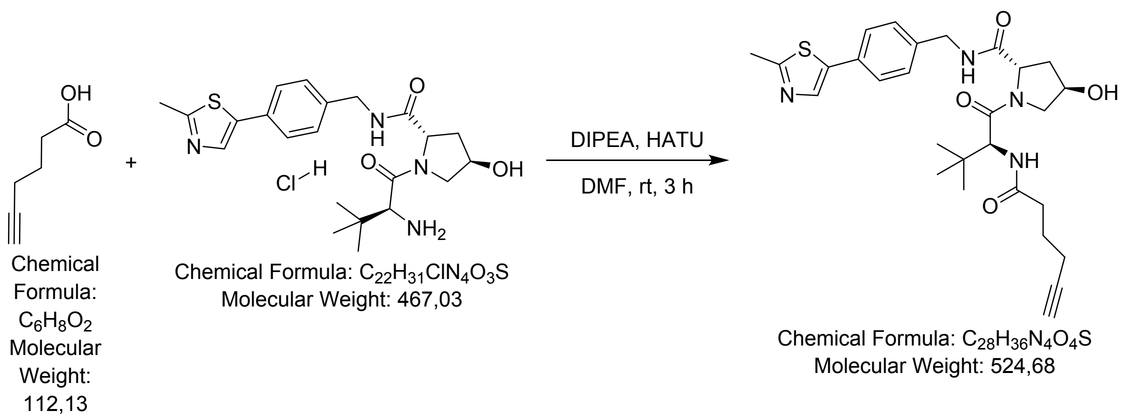

Hex-5-ynoic acid (71 µL, 642 µmol, 1.0 eq.) and VHL hydrochloride (300 mg, 642 µmol, 1.0 eq.) were added to a solution of 10 mL abs. DMF with DIPEA (600 µL, 3.43 mmol, 5.3 eq.) and HATU (611 mg, 1.61 mmol, 2.5 eq.) under argon. The solution was stirred at rt for three hours and monitored via TLC, whereafter the reaction was quenched by the addition of 25 mL of H_2O. The solution was extracted three times with 25 mL EE, and the united organic phases were dried over Na_2SO_4. The workup was completed via flash column chromatography (DCM:EtOH) (98:2 → 0:1). The product was obtained as a white powdery residue.

Theoretical Yield: 337 mg

Yield: 309 mg ≡ 92%

^{1}H NMR (250 MHz, $(CD_3)_2SO$): 8.99 (d, $J_{4H\text{-}H}$ = 2.5 Hz, 1H), 8.56 (t, $J_{3H\text{-}H}$ = 6.0 Hz, 1H), 7.91 (d, $J_{3H\text{-}H}$ = 9.3 Hz, 1H), 7.45 – 7.35 (m, 4H), 5.12 (d, $J_{4H\text{-}H}$ = 3.6 Hz, 1H), 4.53 (d, $J_{3H\text{-}H}$ = 9.2 Hz, 1H), 4.49 – 4.31 (m, 3H), 4.21 (dd, $J_{2H\text{-}H}$ = 15.9, $J_{3H\text{-}H}$ = 5.6 Hz, 1H), 3.65 (s, 2H), 3.03 (s, 2H), 2.85 – 2.74 (m, 1H), 2.44 (s, 3H), 2.36 – 1.56 (m, 6H), 0.95 (d, *J* = 9.3 Hz, 9H) ppm.

MS (ESI+): *m/z* = 525.25 $[M+H]^+$

TLC: (ACN : H_2O) (1:1) R_f = 0.45

C) Synthesis of LIMK-PROTACs:

Synthesis of N-(5-(1-(2,6-dichlorophenyl)-3-(difluoromethyl)-1H-pyrazol-5-yl)thiazol-2-yl)-2-(4-(((2-(2,6-dioxopiperidin-3-yl)-1,3-dioxoisoindolin-4-yl)oxy)methyl)-1H-1,2,3-triazol-1-yl)acetamide (**21a; TH685**)

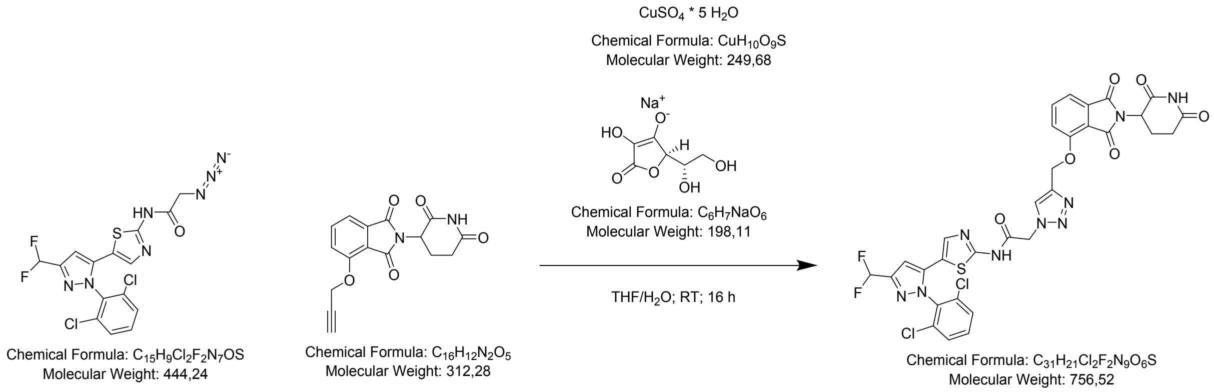

9a **14** **21a (TH685)**

In a flask was weighed intermediate 14 (19 mg, 0.06 mmol), (+)-sodium L-ascorbate (4.5 mg, 0.023 mmol), copper(II) sulfate powder (1.4 mg, 0.006 mmol), and intermediate 9a (26.5 mg, 0.06 mmol). The reaction mixture was treated with THF (1 mL, ca. 0.1M) and 3−4 drops of water, and stirred at rt for 16 h. LC-MS analysis of the crude reaction mixture indicated clean conversion to the desired product. The solvent of the reaction was evaporated and the crude residue was flashed on a RP-C18 column (ACN/H_2O) to obtain the desired product as a white solid.

Theoretical Yield: 0.045 g

Yield: 0.023 g ≡ 51 %

^{1}H NMR (500 MHz, (CD_3OD): δ = 8.17 (s, 1H), 7.79 – 7.73 (m, 1H), 7.63 – 7.54 (m, 4H), 7.47 (d, *J* = 7.2 Hz, 1H), 7.45 (s, 1H), 7.02 (s, 1H), 6.83 (t, *J* = 54.6 Hz, 1H), 5.50 – 5.41 (m, 4H), 5.09 (dd, *J* = 12.6, 5.5 Hz, 1H), 2.84 (ddd, *J* = 19.0, 13.9, 5.2 Hz, 1H), 2.78 – 2.63 (m, 2H), 2.11 (ddd, *J* = 9.9, 8.0, 2.8 Hz, 1H).

^{13}C NMR (126 MHz, (CD_3OD): δ = 174.60, 171.50, 168.49, 167.31, 166.06, 160.44, 156.95, 150.42 (t, *J* = 29.4 Hz), 144.32, 139.70, 138.80, 137.93, 136.65, 135.64, 135.17, 134.14, 130.41, 127.62, 121.58, 119.61, 118.84, 117.22, 112.25 (t, *J* = 233.8 Hz), 104.47, 63.83, 52.78, 50.47, 32.17, 23.64.

LC-MS (ESI+): *m/z* = 756.05 $[M+H]^+$

HPLC: t_R = 5.578, purity ≥ 95% (DAD1 C, Sig=320,150).

HRMS: m/z Calcd for $C_{31}H_{22}Cl_2F_2N_9O_6S_1$ $[M + 1]^+$ = 756.07534. Found $[M + 1]^+$ = 756.07504.

Synthesis of N-(5-(1-(2,6-dichlorophenyl)-3-(difluoromethyl)-1H-pyrazol-5-yl)thiazol-2-yl)-6-(4-(((2-(2,6-dioxopiperidin-3-yl)-1,3-dioxoisoindolin-4-yl)oxy)methyl)-1H-1,2,3-triazol-1-yl)hexanamide (**21b; TH694**)

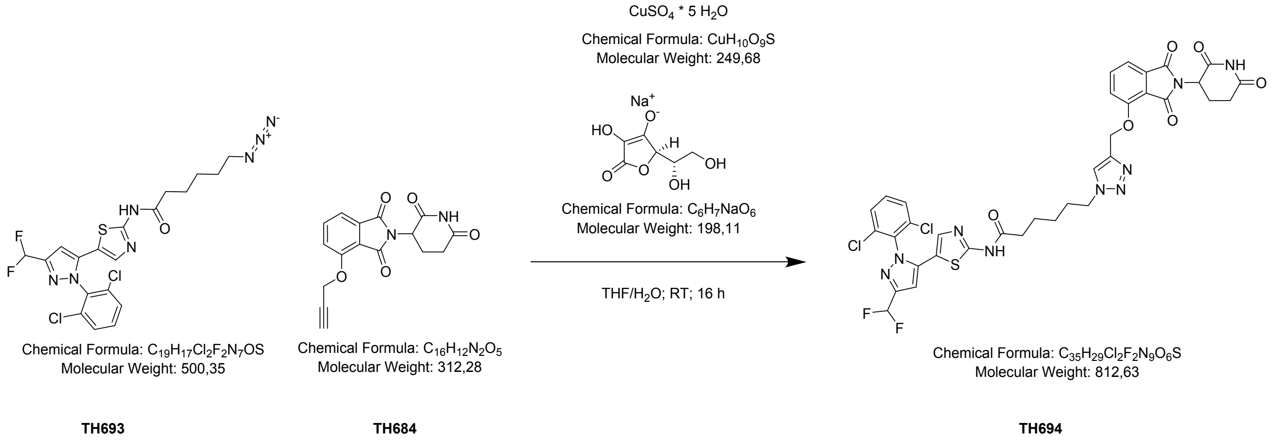

In a flask was weighed intermediate TH684 (19 mg, 0.06 mmol), (+)-sodium L-ascorbate (4.5 mg, 0.023 mmol), copper(II) sulfate powder (1.4 mg, 0.006 mmol), and intermediate TH693 (30 mg, 0.06 mmol). The reaction mixture was treated with THF (1 mL, ca. 0.1M) and 3−4 drops of water, and stirred at rt for 16 h. LC-MS analysis of the crude reaction mixture indicated clean conversion to the desired product. The solvent of the reaction was evaporated, and the crude residue was flashed on a RP-C18 column (ACN/H_2O) to obtain the desired product as a white solid.

Theoretical Yield: 0.049 g

Yield: 0.023 g ≡ 51 %

^{1}H NMR (500 MHz, (CD_3OD): δ = 8.10 (s, 1H), 7.75 (dd, *J* = 8.4, 7.3 Hz, 1H), 7.66 – 7.58 (m, 3H), 7.56 (d, *J* = 8.5 Hz, 1H), 7.46 (d, *J* = 7.2 Hz, 1H), 7.36 (s, 1H), 7.00 (s, 1H), 6.84 (t, *J* = 54.7 Hz, 1H), 5.42 (s, 2H), 5.09 (dd, *J* = 12.7, 5.5 Hz, 1H), 4.42 (t, *J* = 7.0 Hz, 2H), 2.85 (ddd, *J* = 17.5, 13.9, 5.2 Hz, 1H), 2.78 – 2.64 (m, 2H), 2.42 (t, *J* = 7.4 Hz, 2H), 2.15 – 2.07 (m, 1H), 1.97 – 1.88 (m, 2H), 1.73 – 1.63 (m, 2H), 1.35 – 1.28 (m, 2H).

^{13}C NMR (126 MHz, (CD_3OD): δ = 174.59, 173.38, 171.50, 168.47, 167.27, 160.87, 156.95, 150.40 (t, *J* = 29.4 Hz), 144.12, 139.93, 138.60, 137.91, 136.66, 135.78, 135.17, 134.07, 130.41, 125.82, 121.61, 118.96, 118.86, 117.21, 112.27 (t, *J* = 233.8 Hz), 104.36, 63.87, 51.17, 50.47, 35.97, 32.17, 30.79, 26.81, 25.30, 23.63.

LC-MS (ESI+): *m/z* = 812.15 $[M+H]^+$

HPLC: t_R = 4.147, purity ≥ 95% (DAD1 C, Sig=320,150).

HRMS: m/z Calcd for $C_{35}H_{30}Cl_2F_2N_9O_6S_1$ $[M + 1]^+$ = 812.13794. Found $[M + 1]^+$ = 812.13758.

Synthesis of N-(5-(1-(2,6-dichlorophenyl)-3-(difluoromethyl)-1H-pyrazol-5-yl)-1,3-thiazol-2-yl)(4-((3-(2,4-dioxo-1,3-diazinan-1-yl)-2-methylphenoxy)methyl)-1,2,3-triazol-1-yl)acetamide (**22a; THNAN100**)

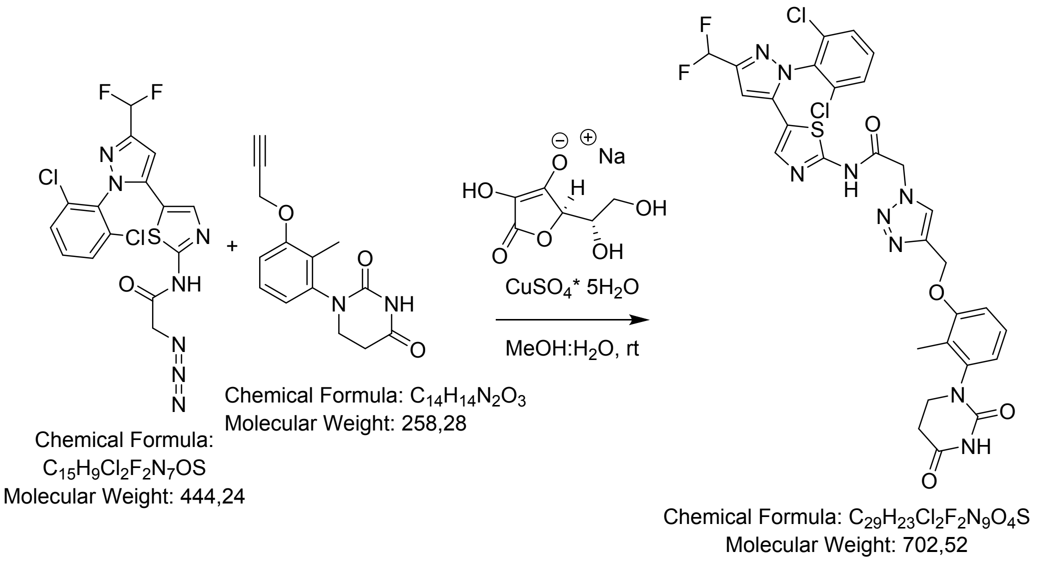

2-azido-*N*-(5-(1-(2,6-dichlorophenyl)-3-(difluoromethyl)-1*H*-pyrazol-5-yl)thiazol-2-yl)acetamide (**13**) (45 mg, 101 µmol, 1.0 eq.) and 1-(2-methyl-3-(prop-2-yn-1-yloxy)phenyl)-1,3-diazinane-2,4-dione (26 mg, 101 µmol, 1.0 eq.) were added to a solution of 5 mL MeOH:H_2O (1:1) with copper sulphate pentahydrate (13 mg, 51 µmol, 0.5 eq.) and sodium ascorbate (8 mg, 36 µmol, 0.35 eq.). The solution was stirred at rt for 16 h, after which it was worked up by adding 10 mL of water and extracting the solution three times with 10 mL of DCM. The united organic phases were dried over Na_2SO_4 and further purified by flash column chromatography (DCM:EtOH) (98:2→ 0:1). Since the product was still not pure, it was purified again by preparative RP-HPLC (H_2O:ACN) (98:1 → 0:1). The product was obtained as a brown oily substance.

Theoretical Yield: 71 mg

Yield: 17 mg ≡ 24%

^{1}H NMR (500 MHz, $(CD_3)_2SO$): δ =12.88 (s, 1H), 10.32 (s, 1H), 8.27 (s, 1H), 8.00 – 7.64 (m, 4H), 7.26 – 6.96 (m, 2H), 7.13 (t, $J_{2H\text{-}F}$ = 54.2 Hz, 1H), 6.92 (dd, $J_{3H\text{-}H}$ = 7.8, $J_{4H\text{-}H}$ = 1.0 Hz, 1H), 5.46 (s, 2H), 5.21 (s, 2H), 3.84 – 3.68 (m, 1H), 3.54 – 3.44 (m, 1H), 2.82 – 2.63 (m, 3H), 2.01 (s, 3H) ppm.

^{13}C NMR (126 MHz, $(CD_3)_2SO$): δ = 171.21, 162.79, 157.11, 152.22, 142.24, 138.68, 134.92, 134.14, 134.12, 129.99, 127.18, 126.60, 124.79, 120.05, 111.50, 111.47, 103.69, 62.24, 51.87, 31.55, 31.24, 11.27, 11.24 ppm.

MS (ESI–): *m/z* = 702.05 [M–H]$^-$

HRMS: m/z Calcd for $C_{29}H_{23}Cl_2F_2N_9O_4S$ [M - H]$^-$ = 700.0866. Found [M - H]$^-$ = 700.0869.

Synthesis of N-(5-(1-(2,6-dichlorophenyl)-3-(difluoromethyl)-1H-pyrazol-5-yl)-1,3-thiazol-2-yl)-5-(4-((3-(2,4-dioxo-1,3-diazinan-1-yl)-2-methylphenoxy)methyl)-1H-1,2,3-triazol-1-yl)pentanamide (**22b; THNAN69**)

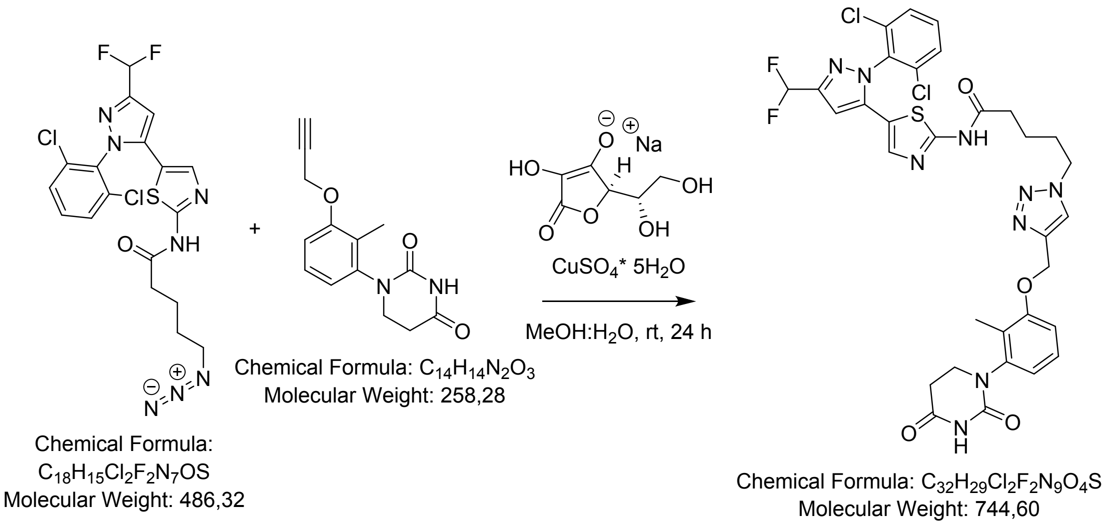

5-azido-*N*-(5-(1-(2,6-dichlorophenyl)-3-(difluoromethyl)-1*H*-pyrazol-5-yl)thiazol-2-yl)pentanamide (**15**) (40 mg, 82 µmol, 1.0 eq.) and 1-(2-methyl-3-(prop-2-yn-1-yloxy)phenyl)-1,3-diazinane-2,4-dione (21 mg, 82 µmol, 1.0 eq.) were added to a solution of 5 mL MeOH:H_2O (1:1) with copper sulphate pentahydrate (10 mg, 41 µmol, 0.5 eq.) and sodium ascorbate (6 mg, 29 µmol, 0.35 eq.). The solution was stirred at rt for 16 h, after which it was worked up by adding 10 mL of water and extracting the solution three times with 10 mL of DCM. The united organic phases were dried over Na_2SO_4 and further purified by flash column chromatography (DCM:EtOH) (98:2→ 0:1). Since the product was still not pure, it was purified again by RP-flash column chromatography (H_2O:ACN) (9:1 → 0:1). The product was obtained as a brown oil.

Theoretical Yield: 61 mg

Yield: 21 mg ≡ 34%

^{1}H NMR (500 MHz, $(CD_3)_2SO$): δ =12.30 (s, 1H), 10.31 (s, 1H), 8.23 (s, 1H), 7.98 – 7.69 (m, 4H), 7.27 – 6.99 (m, 3H), 6.89 (dd, $J_{3H\text{-}H}$ = 7.8, $J_{4H\text{-}H}$ = 1.1 Hz, 1H), 5.16 (s, 2H), 4.37 (t, $J_{3H\text{-}H}$ = 7.0 Hz, 2H), 3.79 – 3.69 (m, 1H), 3.47 (m, 1H), 2.73 (s, 3H), 2.43 (t, $J_{3H\text{-}H}$ = 7.4 Hz, 2H), 1.98 (s, 3H), 1.82 (p, $J_{3H\text{-}H}$ = 7.5 Hz, 2H), 1.52 (p, $J_{3H\text{-}H}$ = 7.6 Hz, 2H) ppm.

^{13}C NMR (126 MHz, $(CD_3)_2SO$): δ = 171.19, 162.79, 157.11, 152.20, 143.31, 142.21, 134.91, 134.02, 129.96, 127.15, 124.83, 124.78, 120.02, 111.53, 103.53, 62.39, 49.55, 31.54, 29.61, 21.89, 11.24 ppm.

MS (ESI–): *m/z* = 744.15 $[M–H]^-$

HRMS: m/z Calcd for $C_{32}H_{29}Cl_2F_2N_9O_4S$ $[M + H]^+$ = 744.1481. Found $[M + H]^+$ = 744.1472.

Synthesis of N-(5-(1-(2,6-dichlorophenyl)-3-(difluoromethyl)-1H-pyrazol-5-yl)-1,3-thiazol-2-yl)-6-(4-((3-(2,4-dioxo-1,3-diazinan-1-yl)-2-methylphenoxy)methyl)-1,2,3-triazol-1-yl)hexanamide (**22c, THNAN99**)

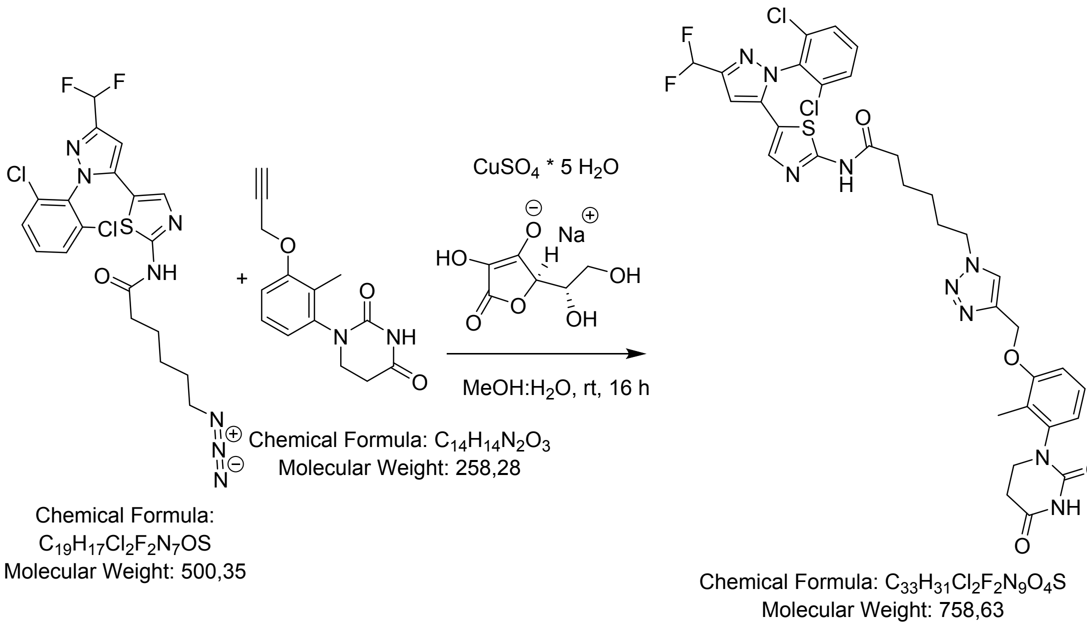

6-azido-*N*-(5-(1-(2,6-dichlorophenyl)-3-(difluoromethyl)-1*H*-pyrazol-5-yl)thiazol-2-yl)hexanamide (**16**) (77 mg, 155 µmol, 1.0 eq.) and 1-(2-methyl-3-(prop-2-yn-1-yloxy)phenyl)-1,3-diazinane-2,4-dione (40 mg, 155 µmol, 1.0 eq.) were added to a solution of 5 mL MeOH:H_2O (1:1) with copper sulphate pentahydrate (19 mg, 77 µmol, 0.5 eq.) and sodium ascorbate (12 mg, 54 µmol, 0.35 eq.). The solution was stirred at rt for 16 h, after which it was worked up by adding 10 mL of water and extracting the solution three times with 10 mL of DCM. The united organic phases were dried over Na_2SO_4 and further purified by flash column chromatography (DCM:EtOH) (98:2→ 0:1). The product was obtained as a brown oil.

Theoretical Yield: 117 mg

Yield: 33 mg ≡ 28%

^{1}H NMR (500 MHz, $(CD_3)_2SO$): δ =12.28 (s, 1H), 10.31 (s, 1H), 8.22 (s, 1H), 7.82 – 7.65 (m, 4H), 7.26 – 7.08 (m, 4H), 6.89 (d, $J_{3H\text{-}H}$ = 7.8 Hz, 1H), 5.15 (s, 2H), 4.36 (t, $J_{3H\text{-}H}$ = 7.1 Hz, 2H), 3.80 – 3.69 (m, 1H), 3.53 – 3.42 (m, 1H), 2.82 – 2.58 (m, 2H), 2.38 (t, $J_{3H\text{-}H}$ = 7.4 Hz, 2H), 1.98 (s, 3H), 1.82 (p, $J_{3H\text{-}H}$ = 7.3 Hz, 2H), 1.58 (p, $J_{3H\text{-}H}$ = 7.5 Hz, 2H), 1.29 – 1.14 (m, 3H), ppm.

^{13}C NMR (126 MHz, $(CD_3)_2SO$): δ = 170.46, 141.47, 137.83, 133.28, 129.22, 126.42, 123.98, 119.28, 112.65, 110.80, 110.77, 108.95, 102.81, 61.64, 48.92, 44.39, 34.21, 30.81, 29.12, 25.09, 23.55, 10.51 ppm.

MS (ESI–): *m/z* = 758.15 $[M–H]^–$

HRMS: m/z Calcd for $C_{33}H_{31}Cl_2F_2N_9O_4S$ $[M + H]^+$ = 758.1638. Found $[M + H]^+$ = 758.1627.

Synthesis of N-(5-(1-(2,6-dichlorophenyl)-3-(difluoromethyl)-1H-pyrazol-5-yl)thiazol-2-yl)-5-(4-((2-methyl-3-(3-methyl-2,4-dioxotetrahydropyrimidin-1(2H)-yl)phenoxy)methyl)-1H-1,2,3-triazol-1-yl)pentanamide (**22d; THNAN69-NC**)

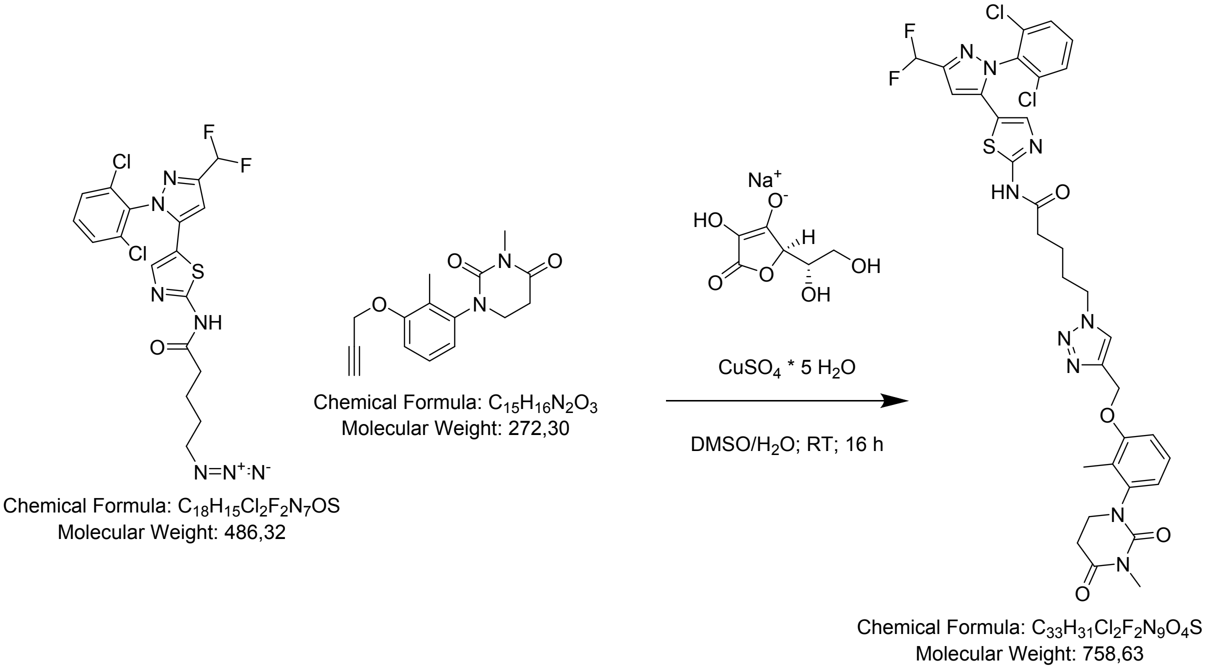

3-methyl-1-(2-methyl-3-(prop-2-yn-1-yloxy)phenyl)dihydropyrimidine-2,4(1H,3H)-dione (4.1 mg, 15 µmol) and 5-azido-N-(5-(1-(2,6-dichlorophenyl)-3-(difluoromethyl)-1H-pyrazol-5-yl)thiazol-2-yl)pentanamide (7.3 mg, 15 µmol) were dissolved in DMSO (2 mL). CuSO4·5H2O (1.1 mg, 4.5 µmol) and sodium ascorbate (0.9 mg, 4.5 µmol) were dissolved in water (0.5 mL) and added to the reaction mixture. The solution was stirred at rt for 18 h. All volatiles were removed under reduced pressure, and the crude product was purified by flash chromatography using acetonitrile/water as an eluent to obtain the title compound as a colourless solid (9.8 mg, 86%).

Theoretical Yield: 11.4 mg

Yield: 9.8 mg ≡ 86%

^{1}H NMR (400 MHz, $(CD_3)_2SO$): δ =12.31 (s, 1H), 8.24 (s, 1H), 7.82 – 7.70 (m, 3H), 7.68 (s, 1H), 7.24 (s, 1H), 7.22 – 7.18 (m, 1H), 7.15 (s, 1H), 7.06 (t, J = 54.3 Hz, 1H), 6.89 (dd, J = 7.8, 1.2 Hz, 1H), 5.17 (s, 2H), 4.38 (t, J = 7.0 Hz, 2H), 3.78 – 3.67 (m, 1H), 3.52 – 3.41 (m, 1H), 3.03 (s, 3H), 2.96 – 2.75 (m, 2H), 2.43 (t, J = 7.4 Hz, 2H), 1.98 (s, 3H), 1.82 (p, J = 7.1 Hz, 2H), 1.52 (p, J = 7.4 Hz, 2H).

^{13}C NMR (101 MHz, $(CD_3)_2SO$): δ = 171.23, 169.76, 159.01, 156.67, 152.26, 148.21, 142.82, 142.24, 138.44, 138.12, 134.45, 133.83, 133.55, 129.49, 126.71, 124.33, 124.25, 119.46, 116.45, 111.11, 103.05, 61.91, 49.06, 43.51, 33.97, 31.35, 29.13, 27.20, 21.41, 10.76 ppm.

MS (ESI–): *m/z* = 758.10 $[M+H]^+$

Synthesis of 1-(2-((5-(1-(2,6-dichlorophenyl)-3-(difluoromethyl)-1H-pyrazol-5-yl)thiazol-2-yl)amino)-2-oxoethyl)-N-((S)-1-((2S,4R)-4-hydroxy-2-((4-(2-methylthiazol-5-yl)benzyl)carbamoyl)pyrrolidin-1-yl)-3,3-dimethyl-1-oxobutan-2-yl)-1H-1,2,3-triazole-4-carboxamide (**23a; THNAN46**)

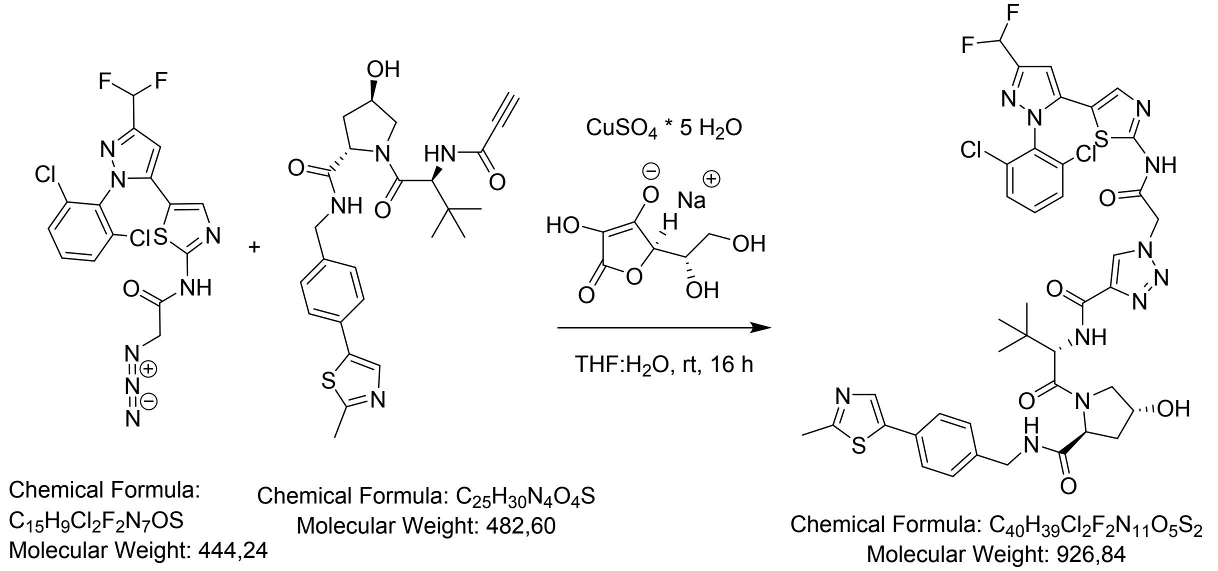

(2*S*,4*R*)-1-((*S*)-3,3-dimethyl-2-propiolamidobutanoyl)-4-hydroxy-*N*-(4-(2-methylthiazol-5-yl)benzyl)pyrrolidine-2-carboxamide (**19**) (54 mg, 112 µmol, 1.0 eq.) and 2-azido-*N*-(5-(1-(2,6-dichlorophenyl)-3-(difluoromethyl)-1*H*-pyrazol-5-yl)thiazol-2-yl)acetamide (**13**) (50 mg, 112 µmol, 1.0 eq.) were added to 6 mL of THF:H_2O (1:1) with copper sulphate pentahydrate (4 mg, 16 µmol, 0.15 eq.) and sodium ascorbate (8.3 mg, 39 µmol, 0.35 eq.). The reaction mixture was stirred at rt for 16 h. Subsequently, the workup of the reaction consisted of an extraction with 10 mL DCM three times, after which the united organic phases were dried over Na_2SO_4. Lastly, the reaction was purified via flash column chromatography (DCM:EtOH) (98:2→ 0:1). The product was obtained as a light brown oil.

Theoretical Yield: 104 mg

Yield: 27 mg ≡ 26%

^{1}H NMR (500 MHz, $(CD_3)_2SO$): δ = 12.7 (s, 1H), 8.98 (s, 1H), 8.63 (s, 1H), 7.85 (s, 1H), 7.81 – 7.67 (m, 4H), 7.42 (s, 4H), 7.28 (s, 1H), 7.13 (t, $J_{2H\text{-}F}$ = 54.2 Hz, 1H), 5.50 (s, 2H), 5.17 (d, $J_{4H\text{-}H}$ = 3.6 Hz, 1H), 4.74 (d,

$J_{3H\text{-}H}$ = 9.5 Hz, 1H), 4.50 (t, $J_{3H\text{-}H}$ = 7.9 Hz, 1H), 4.39 (d, $J_{3H\text{-}H}$ = 6.1 Hz, 1H), 4.27 (dd, $J_{2H\text{-}H}$ = 15.8, $J_{3H\text{-}H}$ = 5.5 Hz, 1H), 3.70 (d, $J_{4H\text{-}H}$ = 4.7 Hz, 2H), 2.46 (s, 3H), 2.22 – 1.82 (m, 2H), 1.23 (s, 3H), 1.02 (s, 9H) ppm.

^{13}C NMR (126 MHz, $(CD_3)_2SO$): δ = 172.20, 169.57, 165.36, 159.15, 151.92, 148.71, 148.26, 142.39, 139.90, 138.75, 134.93, 134.20, 134.09, 131.63, 130.21, 130.00, 129.22, 128.87, 127.93, 117.51, 111.51, 103.59, 69.39, 59.25, 57.20, 56.51, 52.25, 26.70, 16.42 ppm.

MS (ESI–): *m/z* = 926.10 [M–H]$^-$

HRMS: m/z Calcd for $C_{40}H_{39}Cl_2F_2N_{11}O_5S_2$ [M + H]$^+$ = 926.1995. Found [M + H]$^+$ = 926.1982.

Synthesis of 1-(5-((5-(1-(2,6-dichlorophenyl)-3-(difluoromethyl)-1H-pyrazol-5-yl)thiazol-2-yl)amino)-5-oxopentyl)-N-((S)-1-((2S,4R)-4-hydroxy-2-((4-(2-methylthiazol-5-yl)benzyl)carbamoyl)pyrrolidin-1-yl)-3,3-dimethyl-1-oxobutan-2-yl)-1H-1,2,3-triazole-4-carboxamide (**23b, THNAN105**)

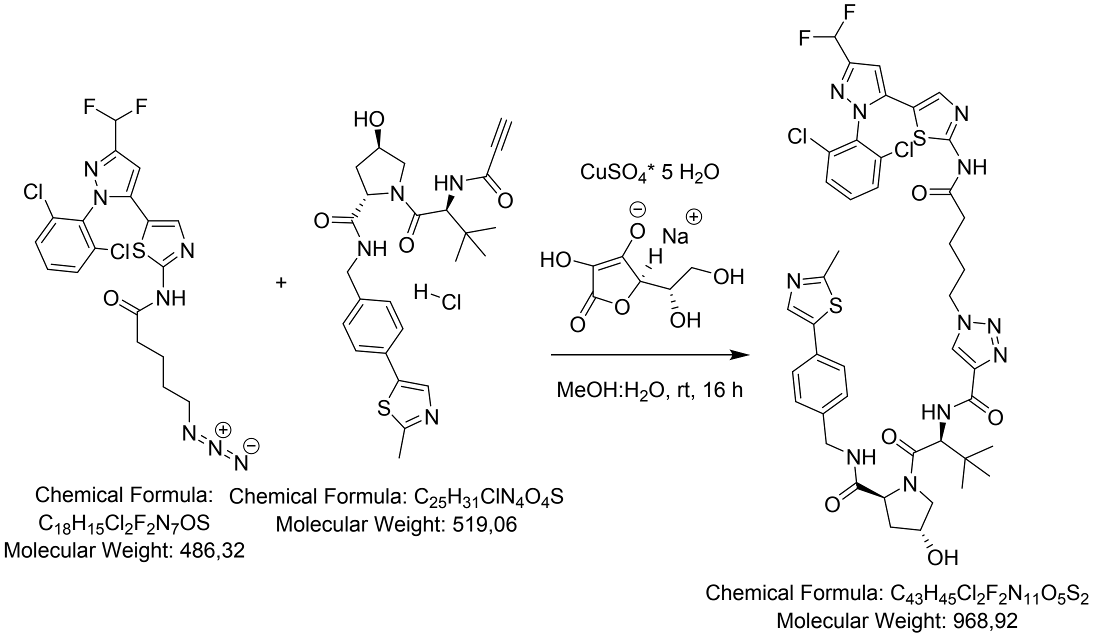

(2*S*,4*R*)-1-((*S*)-3,3-dimethyl-2-propiolamidobutanoyl)-4-hydroxy-*N*-(4-(2-methylthiazol-5-yl)benzyl)pyrrolidine-2-carboxamide (**19**) (48 mg, 92 µmol, 1.0 eq.) and 5-azido-*N*-(5-(1-(2,6-dichlorophenyl)-3-(difluoromethyl)-1*H*-pyrazol-5-yl)thiazol-2-yl)pentanamide (**15**) (45 mg, 93 µmol, 1.0 eq.) were added to 6 mL of $MeOH:H_2O$ (1:1) with copper sulphate pentahydrate (11 mg, 46 µmol, 0.5 eq.) and sodium ascorbate (7 mg, 32 µmol, 0.35 eq.). The reaction mixture was stirred at rt for 16 h. Subsequently the workup of the reaction consisted of an extraction with 10 mL DCM three times, after which the united organic phases were dried over Na_2SO_4. Lastly, the reaction was purified via flash column chromatography (DCM:EtOH) (98:2→ 0:1). The product was obtained as a light brown oil.

^{1}H NMR (500 MHz, $(CD_3)_2SO$): δ = 12.30 (s, 1H), 8.98 (s, 1H), 8.66 (s, 1H), 8.63 (t, J_{3H-H} = 6.1 Hz, 1H), 7.82 – 7.66 (m, 4H), 7.42 (s, 4H), 7.23 (t, J_{4H-H} = 1.0 Hz, 1H), 7.12 (t, J_{2H-F} = 54.2 Hz, 1H), 5.16 (d, J_{4H-H} = 3.7 Hz, 1H), 4.73 (d, J_{3H-H} = 9.6 Hz, 1H), 4.48 (t, J_{3H-H} = 8.2 Hz, 1H), 4.44 – 4.35 (m, 4H), 4.30 – 4.24 (m, 1H), 3.74 – 3.63 (m, 2H), 2.44 (m, 5H), 2.12 – 1.89 (m, 2H), 1.83 (p, J_{3H-H} = 7.1 Hz, 2H), 1.55 – 1.45 (m, 2), 1.00 (s, 9H) ppm.
^{13}C NMR (126 MHz, $(CD_3)_2SO$): δ =171.73, 171.20, 171.18, 169.13, 159.02, 158.87, 151.45, 148.20, 147.80, 141.94, 139.42, 138.44, 138.09, 134.45, 133.83, 133.55, 129.74, 129.57, 129.49, 128.90, 128.74, 128.71, 127.52, 127.45, 127.36, 126.59, 116.47, 111.06, 103.06, 103.03, 68.92, 58.77, 56.72, 55.97, 49.54, 49.51, 36.11, 33.96, 28.94, 26.21, 26.16, 21.27, 15.94 ppm.

Theoretical Yield: 90 mg

Yield: 28 mg ≡ 31%

MS (ESI–): *m/z* = 968.25 [M–H]$^-$

HRMS: m/z Calcd for $C_{43}H_{45}Cl_2F_2N_{11}O_5S_2$ [M + H]$^+$ = 968.2464. Found [M + H]$^+$ = 968.2459.

Synthesis of 1-(6-((5-(1-(2,6-dichlorophenyl)-3-(difluoromethyl)-1H-pyrazol-5-yl)thiazol-2-yl)amino)-6-oxohexyl)-N-((S)-1-((2S,4R)-4-hydroxy-2-((4-(2-methylthiazol-5-yl)benzyl)carbamoyl)pyrrolidin-1-yl)-3,3-dimethyl-1-oxobutan-2-yl)-1H-1,2,3-triazole-4-carboxamide (**23c, THNAN71**)

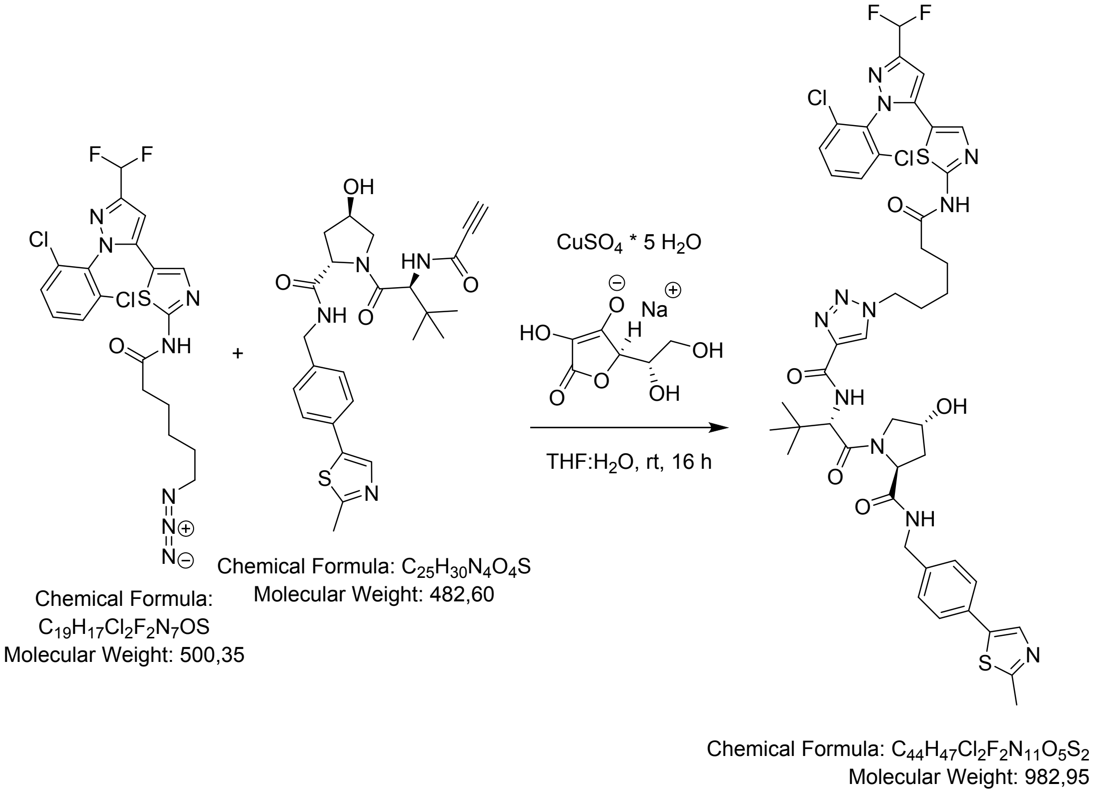

(2*S*,4*R*)-1-((*S*)-3,3-dimethyl-2-propiolamidobutanoyl)-4-hydroxy-*N*-(4-(2-methylthiazol-5-yl)benzyl)pyrrolidine-2-carboxamide (**19**) (48 mg, 100 µmol, 1.0 eq.) and 6-azido-*N*-(5-(1-(2,6-

dichlorophenyl)-3-(difluoromethyl)-1*H*-pyrazol-5-yl)thiazol-2-yl)hexanamide (**16**) (50 mg, 100 µmol, 1.0 eq.) were added to 6 mL of THF:H_2O (1:1) with copper sulphate pentahydrate (12 mg, 50 µmol, 0.5 eq.) and sodium ascorbate (7 mg, 34 µmol, 0.35 eq.). The reaction mixture was stirred at rt for 16 h. Subsequently, the workup of the reaction consisted of an extraction with 10 mL DCM three times, after which the united organic phases were dried over Na_2SO_4. Lastly, the reaction was purified via flash column chromatography (DCM:EtOH) (98:2→ 0:1). The product was obtained as a brittle, white/green substance.

Theoretical Yield: 98 mg

Yield: 50 mg ≡ 51%

^{1}H NMR (500 MHz, $(CD_3)_2SO$): δ = 12.27 (s, 1H), 8.99 (s, 1H), 8.66 (s, 1H), 8.63 (t, J_{3H-H} = 6.1 Hz, 1H), 7.83 – 7.64 (m, 4H), 7.42 (s, 4H), 7.23 (s, 1H), 7.13 (t, J_{2H-F} = 54.2 Hz, 1H), 5.15 (d, J_{4H-H} = 3.6 Hz, 1H), 4.73 (d, J_{3H-H} = 9.6 Hz, 1H), 4.51 – 4.36 (m, 5H), 4.28 (dd, J_{2H-H} = 15.8, J_{3H-H} = 5.6 Hz, 1H), 3.75 – 3.65 (m, 2H), 2.46 (s, 3H), 2.38 (t, J_{3H-H} = 7.5 Hz, 2H), 1.84 (p, J_{3H-H} = 7.3 Hz, 2H), 1.58 (p, J_{3H-H} = 7.6 Hz, 2H), 1.29 – 1.15 (m, 3H), 1.01 (s, 9H) ppm.

^{13}C NMR (126 MHz, $(CD_3)_2SO$): δ =171.68, 171.44, 169.10, 159.03, 158.84, 151.40, 147.76, 141.89, 139.39, 138.42, 138.03, 134.42, 133.83, 133.50, 131.12, 129.72, 129.46, 128.72, 127.43, 126.50, 116.41, 111.04, 103.05, 103.03, 103.01, 68.88, 58.74, 56.69, 55.92, 49.57, 34.44, 29.20, 26.17, 25.22, 23.78, 15.92 ppm.

MS (ESI–): *m/z* = 982.25 $[M–H]^–$

HRMS: m/z Calcd for $C_{44}H_{47}Cl_2F_2N_{11}O_5S_2$ $[M + H]^+$ = 982.2621. Found $[M + H]^+$ = 982.2617.

Synthesis of (2S,4R)-1-((S)-2-(3-(1-(2-((5-(1-(2,6-dichlorophenyl)-3-(difluoromethyl)-1H-pyrazol-5-yl)thiazol-2-yl)amino)-2-oxoethyl)-1H-1,2,3-triazol-4-yl)propanamido)-3,3-dimethylbutanoyl)-4-hydroxy-N-(4-(2-methylthiazol-5-yl)benzyl)pyrrolidine-2-carboxamide (**23d, THNAN47**)

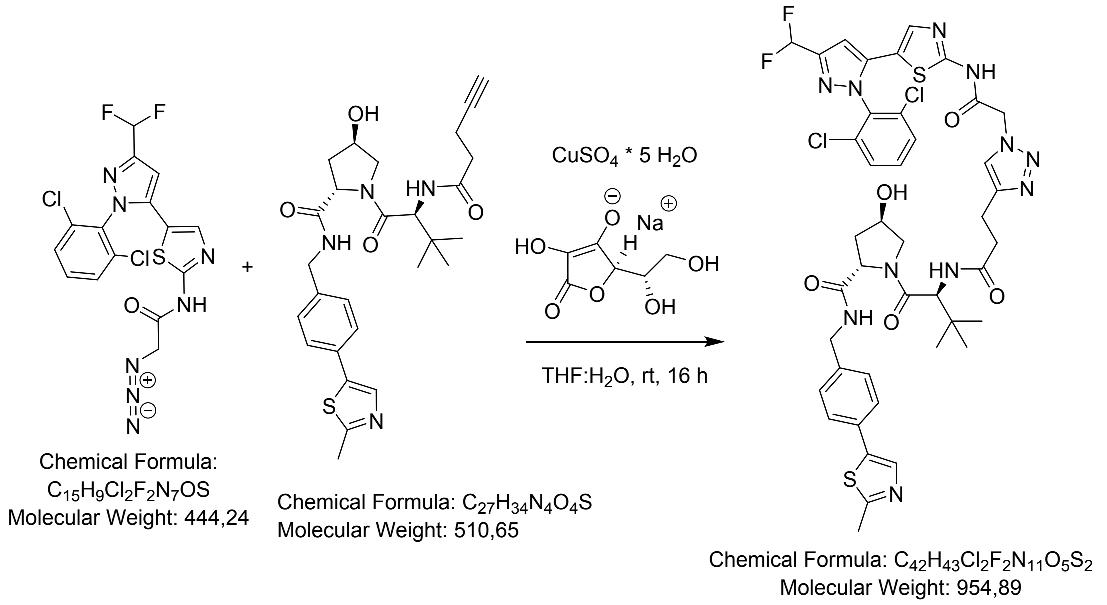

(2*S*,4*R*)-1-((*S*)-2-(pent-4-ynamido)-3,3-dimethylbutanoyl)-4-hydroxy-*N*-(4-(2-methylthiazol-5-yl)benzyl)pyrrolidine-2-carboxamide (**21**) (57 mg, 112 µmol, 1.0 eq.) and 2-azido-*N*-(5-(1-(2,6-dichlorophenyl)-3-(difluoromethyl)-1*H*-pyrazol-5-yl)thiazol-2-yl)acetamide (**13**) (50 mg, 112 µmol, 1.0 eq.) were added to 6 mL of THF:H_2O (1:1) with copper sulphate pentahydrate (4 mg, 16 µmol, 0.15 eq.) and sodium ascorbate (8.3 mg, 39 µmol, 0.35 eq.). The reaction mixture was stirred at rt for 16 h. Subsequently, the workup of the reaction consisted of an extraction with 10 mL DCM three times, after which the united organic phases were dried over Na_2SO_4. Lastly the reaction was purified via flash column chromatography (DCM:EtOH) (98:2→ 0:1). The product was obtained as a light brown oil.

Theoretical Yield: 107 mg

Yield: 70 mg ≡ 65%

^{1}H NMR (500 MHz, $(CD_3)_2SO$): δ = 12.83 (s, 1H), 8.98 (s, 1H), 8.56 (t, J_{3H-H} = 5.9 Hz, 1H), 7.98 (d, J_{3H-H} = 9.2 Hz, 1H), 7.89 – 7.63 (m, 4H), 7.48 – 7.31 (m, 4H), 7.27 (s, 1H), 7.13 (t, J_{2H-F} = 54.2 Hz, 1H), 5.35 (s, 2H), 5.15 (d, J_{4H-H} = 3.5 Hz, 1H), 4.55 (d, J_{3H-H} = 9.3 Hz, 1H), 4.52 – 4.29 (m, 2H), 4.22 (dd, J_{2H-H} = 15.8, J_{3H-H} = 5.3 Hz, 1H), 3.68 (s, 2H), 3.44 (m, 1H), 2.98 – 2.76 (m, 3H), 2.44 (s, 4H), 2.17 – 1.81 (m, 2H), 1.05 (t, J_{3H-H} = 7.0 Hz, 2H), 0.91 (s, 9H) ppm.

^{13}C NMR (126 MHz, $(CD_3)_2SO$): δ = 171.93, 171.07, 169.63, 151.43, 147.71, 146.07, 139.49, 138.22, 134.43, 133.64, 129.64, 129.49, 129.41, 128.63, 128.59, 128.54, 127.42, 123.61, 111.02, 103.21, 68.89, 58.72, 56.48, 56.45, 56.02, 35.17, 34.38, 26.34, 26.25, 26.20, 26.18, 26.16, 26.12, 21.45, 18.54, 15.93 ppm.

MS (ESI–): *m/z* = 954.25 $[M–H]^–$

HRMS: m/z Calcd for $C_{42}H_{43}Cl_2F_2N_{11}O_5S_2$ $[M + H]^+$ = 954.2308. Found $[M + H]^+$ = 954.2302.

(2S,4R)-1-((2S)-2-((3-(1-(5-((5-(1-(2,6-dichlorophenyl)-3-(difluoromethyl)-1H-pyrazol-5-yl)-1,3-thiazol-2-yl)amino)-5-oxopentyl)-1,2,3-triazol-4-yl)propanoyl)amino)-3,3-dimethylbutanoyl)-4-hydroxy-N-(4-(2-methyl-1,3-thiazol-5-yl)benzyl)pyrrolidine-2-carboxamide (**23e, THNAN103**)

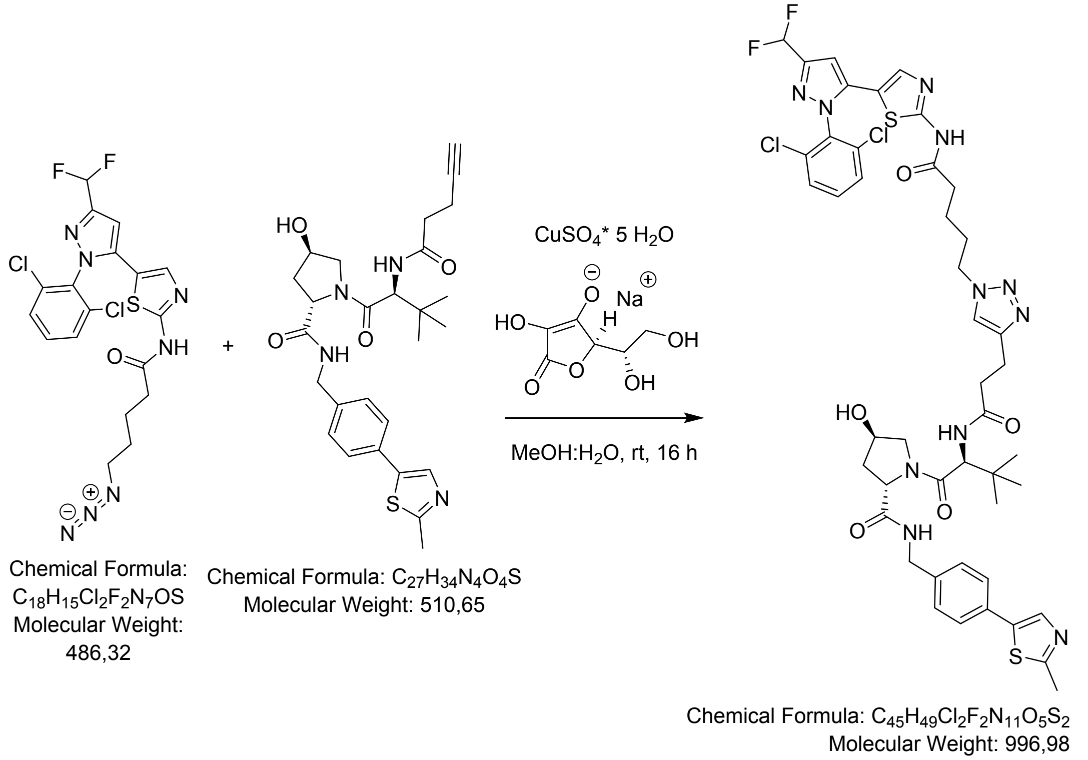

(2*S*,4*R*)-1-((*S*)-2-(pent-4-ynamido)-3,3-dimethylbutanoyl)-4-hydroxy-*N*-(4-(2-methylthiazol-5-yl)benzyl)pyrrolidine-2-carboxamide (**21**) (47 mg, 93 µmol, 1.0 eq.) and 5-azido-*N*-(5-(1-(2,6-dichlorophenyl)-3-(difluoromethyl)-1*H*-pyrazol-5-yl)thiazol-2-yl)pentanamide (**15**) (45 mg, 93 µmol, 1.0 eq.) were added to 6 mL of MeOH:H_2O (1:1) with copper sulphate pentahydrate (12 mg, 46 µmol, 0.5 eq.) and sodium ascorbate (7 mg, 32 µmol, 0.35 eq.). The reaction mixture was stirred at rt for 16 h. Subsequently, the workup of the reaction consisted of an extraction with 10 mL DCM for three times, after which the united organic phases were dried over Na_2SO_4. Lastly, the reaction was purified via flash column chromatography (DCM:EtOH) (98:2→ 0:1). The product was obtained as a light brown oil.

Theoretical Yield: 92 mg

Yield: 36 mg ≡ 39%

^{1}H NMR (500 MHz, $(CD_3)_2SO$): δ =12.30 (s, 1H), 9.00 (s, 1H), 8.55 (t, J_{3H-H} = 6.1 Hz, 1H), 7.93 (d, J_{3H-H} = 9.3 Hz, 1H), 7.82 – 7.68 (m, 3H), 7.67 (s, 1H), 7.44 – 7.35 (m, 4H), 7.22 (s, 1H), 7.12 (t, J_{2H-F} = 54.2 Hz, 1H), 4.54 (d, J = 9.4 Hz, 1H), 4.46 – 4.39 (m, 2H), 4.36 (dd, J_{3H-H} = 4.7, J_{4H-H} = 2.6 Hz, 1H), 4.27 (t, J_{3H-H} = 7.0 Hz, 2H),

4.22 (dd, J_{2H-H} = 15.9, J_{3H-H} = 5.6 Hz, 1H), 2.93 – 2.75 (m, 2H), 2.43 (d, J_{3H-H} = 10.0 Hz, 5H), 2.08 – 1.86 (m, 2H), 1.76 (p, J_{3H-H} = 7.2 Hz, 2H), 1.54 – 1.44 (m, 2H), 0.87 (s, 9H) ppm.

^{13}C NMR (126 MHz, $(CD_3)_2SO$): δ =172.41, 171.69, 171.60, 170.10, 159.48, 139.97, 138.90, 138.56, 134.91, 134.30, 134.00, 130.11, 129.94, 129.12, 127.90, 116.94, 111.52, 103.54, 103.52, 69.37, 59.18, 56.90, 49.31, 35.60, 34.91, 34.46, 29.66, 26.76, 26.70, 21.88, 16.39.

MS (ESI–): *m/z* = 996.30 $[M–H]^-$

HRMS: m/z Calcd for $C_{45}H_{49}Cl_2F_2N_{11}O_5S_2$ $[M - H]^-$ = 996.2777. Found $[M - H]^-$ = 996.2767.

Synthesis of (2S,4R)-1-((S)-2-(3-(1-(6-((5-(1-(2,6-dichlorophenyl)-3-(difluoromethyl)-1H-pyrazol-5-yl)thiazol-2-yl)amino)-6-oxohexyl)-1H-1,2,3-triazol-4-yl)propanamido)-3,3-dimethylbutanoyl)-4-hydroxy-N-(4-(2-methylthiazol-5-yl)benzyl)pyrrolidine-2-carboxamide (**23f; THNAN101**)

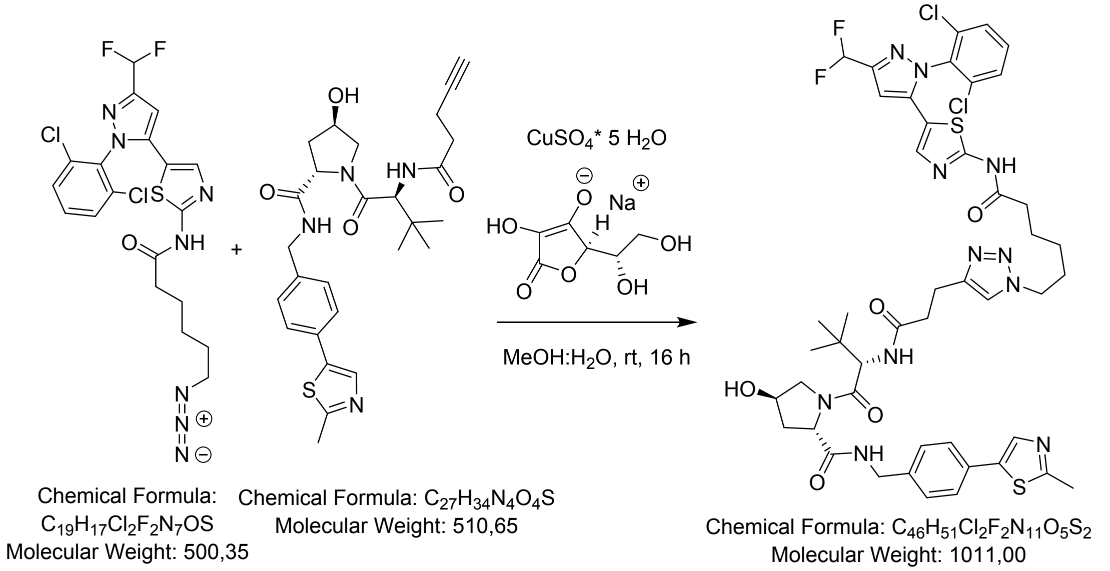

(2*S*,4*R*)-1-((*S*)-2-(pent-4-ynamido)-3,3-dimethylbutanoyl)-4-hydroxy-*N*-(4-(2-methylthiazol-5-yl)benzyl)pyrrolidine-2-carboxamide (**21**) (46 mg, 90 µmol, 1.0 eq.) and 6-azido-*N*-(5-(1-(2,6-dichlorophenyl)-3-(difluoromethyl)-1*H*-pyrazol-5-yl)thiazol-2-yl)hexanamide (**16**) (45 mg, 90 µmol, 1.0 eq.) were added to 6 mL of MeOH:H_2O (1:1) with copper sulphate pentahydrate (11 mg, 45 µmol, 0.5 eq.) and sodium ascorbate (7 mg, 31 µmol, 0.35 eq.). The reaction mixture was stirred at rt for 16 h. Subsequently, the workup of the reaction consisted of an extraction with 10 mL DCM three times, after which the united organic phases were dried over Na_2SO_4. Lastly, the reaction was purified via flash column chromatography (DCM:EtOH) (98:2→ 0:1). The product was obtained as a light brown oil.

Theoretical Yield: 91 mg

Yield: 30 mg ≡ 33%

^{1}H NMR (500 MHz, $(CD_3)_2SO$): δ =12.28 (s, 1H), 8.97 (s, 1H), 8.55 (t, $J_{3H\text{-}H}$ = 6.1 Hz, 1H), 7.94 (d, $J_{3H\text{-}H}$ = 9.3 Hz, 1H), 7.81 – 7.69 (m, 4H), 7.66 (s, 1H), 7.46 – 7.34 (m, 4H), 7.22 (s, 1H), 7.12 (t, $J_{2H\text{-}F}$ = 54.2 Hz, 1H), 5.20 – 5.13 (m, 1H), 4.55 (d, $J_{3H\text{-}H}$ = 9.4 Hz, 1H), 4.46 – 4.39 (m, 2H), 4.36 (t, $J_{4H\text{-}H}$ = 3.8 Hz, 1H), 4.29 – 4.18 (m, 3H), 3.67 (d, $J_{4H\text{-}H}$ = 3.1 Hz, 2H), 2.91 – 2.75 (m, 2H), 2.44 (s, 3H), 2.37 (t, $J_{3H\text{-}H}$ = 7.5 Hz, 2H), 2.09 – 1.88 (m, 2H), 1.76 (p, $J_{3H\text{-}H}$ = 7.3 Hz, 2H), 1.56 (p, $J_{3H\text{-}H}$ = 7.6 Hz, 2H), 1.25 – 1.16 (m, 2H), 0.89 (s, 9H) ppm.

^{13}C NMR (126 MHz, $(CD_3)_2SO$): δ =171.94, 171.15, 169.65, 151.45, 147.73, 145.95, 139.50, 138.07, 134.45, 133.87, 133.53, 129.48, 128.65, 127.44, 121.79, 111.07, 103.05, 68.91, 58.72, 56.45, 48.94, 41.68, 37.95, 35.17, 34.47, 29.48, 26.39, 26.32, 25.41, 23.86, 21.53, 15.94 ppm.

MS (ESI–): *m/z* = 505.75 $[M–2H]^{2–}$

HRMS: m/z Calcd for $C_{46}H_{51}Cl_2F_2N_{11}O_5S_2$ $[M + H]^+$ = 1010.2934. Found $[M + H]^+$ = 1010.2928.

Synthesis of (2S,4R)-1-((S)-2-(4-(1-(2-((5-(1-(2,6-dichlorophenyl)-3-(difluoromethyl)-1H-pyrazol-5-yl)thiazol-2-yl)amino)-2-oxoethyl)-1H-1,2,3-triazol-4-yl)butanamido)-3,3-dimethylbutanoyl)-4-hydroxy-N-(4-(2-methylthiazol-5-yl)benzyl)pyrrolidine-2-carboxamide (**23g; THNAN50**)

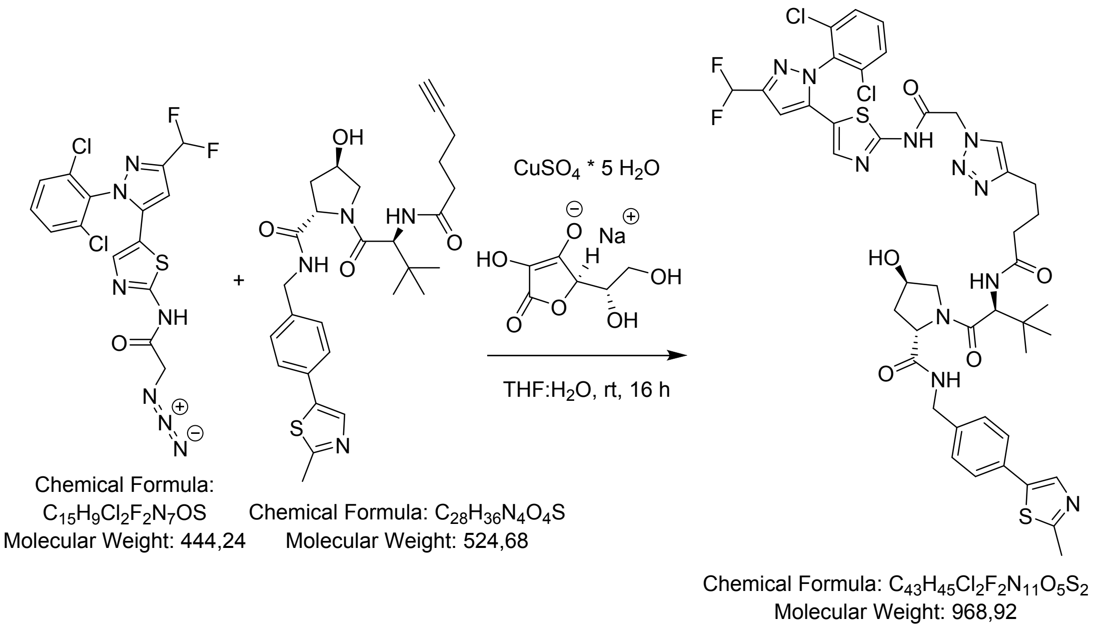

(2*S*,4*R*)-1-((*S*)-2-(hex-5-ynamido)-3,3-dimethylbutanoyl)-4-hydroxy-*N*-(4-(2-methylthiazol-5-yl)benzyl)pyrrolidine-2-carboxamide (**22**) (59 mg, 112 µmol, 1.0 eq.) and 2-azido-*N*-(5-(1-(2,6-dichlorophenyl)-3-(difluoromethyl)-1*H*-pyrazol-5-yl)thiazol-2-yl)acetamide (**13**) (50 mg, 112 µmol, 1.0 eq.) were added to 6 mL of THF:H_2O (1:1) with copper sulphate pentahydrate (4 mg, 16 µmol, 0.15 eq.) and sodium ascorbate (8.3 mg, 39 µmol, 0.35 eq.). The reaction mixture was stirred at rt for 16 h. Subsequently, the workup of the reaction consisted of an extraction with 10 mL DCM three times, after

which the united organic phases were dried over Na_2SO_4. Lastly, the reaction was purified via flash column chromatography (DCM:EtOH) (98:2→ 0:1). The product was obtained as a light brown oil.

Theoretical Yield: 109 mg

Yield: 50 mg ≡ 45%

^{1}H NMR (500 MHz, $(CD_3)_2SO$): δ = 12.84 (s, 1H), 8.98 (s, 1H), 8.57 (t, J_{3H-H} = 6.0 Hz, 1H), 7.93 (d, J_{3H-H} = 9.3 Hz, 1H), 7.89 – 7.64 (m, 4H), 7.46 – 7.35 (m, 4H), 7.27 (s, 1H), 7.13 (t, J_{2H-F} = 54.2 Hz, 1H), 5.36 (s, 2H), 5.14 (d, J_{4H-H} = 3.5 Hz, 1H), 4.57 (d, J = 9.3 Hz, 1H), 4.52 – 4.32 (m, 3H), 4.22 (dd, J_{2H-H} = 15.8, J_{3H-H} = 5.4 Hz, 1H), 3.68 (d, J_{4H-H} = 2.9 Hz, 2H), 2.62 (t, J_{3H-H} = 7.6 Hz, 2H), 2.44 (s, 3H), 2.39 – 1.73 (m, 5H), 0.96 (s, 9H) ppm.

^{13}C NMR (126 MHz, $(CD_3)_2SO$): δ =172.43, 172.25, 170.19, 151.91, 148.20, 146.97, 139.98, 138.73, 134.91, 134.14, 134.12, 131.64, 130.12, 129.98, 129.12, 127.90, 127.86, 124.05, 111.51, 103.66, 103.64, 69.36, 59.18, 56.87, 51.71, 42.15, 38.41, 35.68, 34.97, 26.96, 26.88, 26.81, 25.92, 25.13, 16.42 ppm.

MS (ESI–): *m/z* = 968.25 $[M–H]^-$

HRMS: m/z Calcd for $C_{43}H_{45}Cl_2F_2N_{11}O_5S_2$ $[M + H]^+$ = 968.2464. Found $[M + H]^+$ = 968.2456.

(2S,4R)-1-((2S)-2-((4-(1-(5-((5-(1-(2,6-dichlorophenyl)-3-(difluoromethyl)-1H-pyrazol-5-yl)-1,3-thiazol-2-yl)amino)-5-oxopentyl)-1H-1,2,3-triazol-4-yl)butanoyl)amino)-3,3-dimethylbutanoyl)-4-hydroxy-N-(4-(2-methyl-1,3-thiazol-5-yl)benzyl)pyrrolidine-2-carboxamide (**23h; THNAN106**)

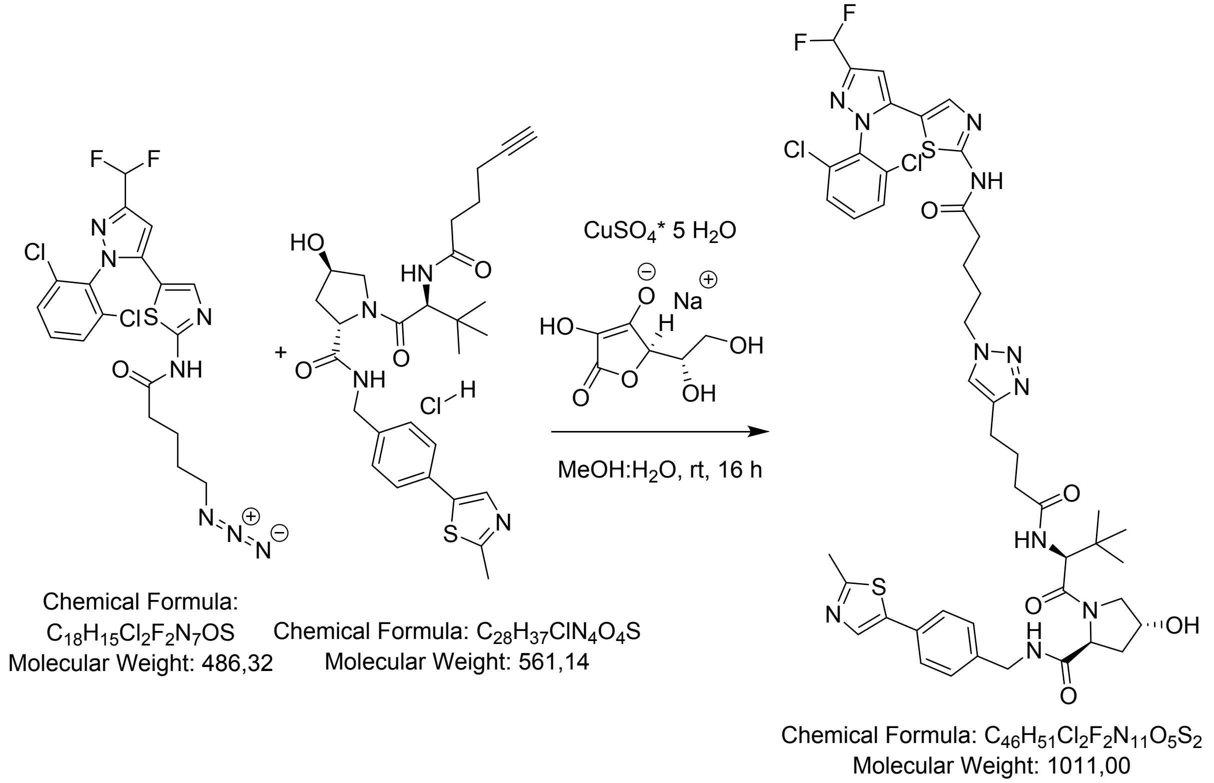

(2*S*,4*R*)-1-((*S*)-2-(hex-5-ynamido)-3,3-dimethylbutanoyl)-4-hydroxy-*N*-(4-(2-methylthiazol-5-yl)benzyl)pyrrolidine-2-carboxamide (**22**) (47 mg, 93 µmol, 1.0 eq.) and 5-azido-*N*-(5-(1-(2,6-dichlorophenyl)-3-(difluoromethyl)-1*H*-pyrazol-5-yl)thiazol-2-yl)pentanamide (**15**) (45 mg, 93 µmol, 1.0 eq.) were added to 6 mL of MeOH:H_2O (1:1) with copper sulphate pentahydrate (12 mg, 46 µmol, 0.5 eq.) and sodium ascorbate (7 mg, 32 µmol, 0.35 eq.). The reaction mixture was stirred at rt for 16 h. Subsequently, the workup of the reaction consisted of an extraction with 10 mL DCM three times, after which the united organic phases were dried over Na_2SO_4. Lastly, the reaction was purified via flash column chromatography (DCM:EtOH) (98:2→ 0:1). The product was obtained as a light brown oil.

Theoretical Yield: 94 mg

Yield: 19 mg ≡ 20%

^{1}H NMR (500 MHz, $(CD_3)_2SO$): δ =12.30 (s, 1H), 8.98 (s, 1H), 8.55 (t, $J_{3H\text{-}H}$ = 6.1 Hz, 1H), 7.83 (s, 1H), 7.82 – 7.69 (m, 3H), 7.67 (s, 1H), 7.45 – 7.34 (m, 4H), 7.22 (s, 1H), 7.07 (t, $J_{2H\text{-}F}$ = 54.2 Hz, 1H), 5.13 (d, $J_{4H\text{-}H}$ = 3.6 Hz, 1H), 4.55 (d, $J_{3H\text{-}H}$ = 9.3 Hz, 1H), 4.47 – 4.32 (m, 3H), 4.28 (t, $J_{3H\text{-}H}$ = 7.0 Hz, 2H), 4.22 (dd, $J_{2H\text{-}H}$ = 15.9, $J_{3H\text{-}H}$ = 5.6 Hz, 1H), 3.71 – 3.63 (m, 2H), 2.58 (td, $J_{3H\text{-}H}$ = 7.7, $J_{4H\text{-}H}$ =2.9 Hz, 2H), 2.43 (d, $J_{3H\text{-}H}$ = 8.5 Hz, 5H), 2.34 – 2.15 (m, 2H), 2.06 – 1.88 (m, 2H), 1.84 – 1.73 (m, 4H), 1.56 – 1.45 (m, 3H), 0.94 (s, 9H) ppm.

^{13}C NMR (126 MHz, $(CD_3)_2SO$): δ =171.95, 171.79, 171.24, 169.71, 159.01, 151.45, 139.50, 138.43, 138.09, 134.44, 133.83, 133.54, 129.65, 129.48, 128.64, 127.43, 121.77, 111.06, 103.06, 103.04, 68.88, 58.71, 56.40, 48.83, 35.19, 34.43, 33.99, 29.16, 26.39, 26.34, 25.44, 24.69, 21.42, 15.94 ppm.

MS (ESI–): *m/z* = 505.85 $[M–2H]^{2–}$

HRMS: m/z Calcd for $C_{46}H_{51}Cl_2F_2N_{11}O_5S_2$ $[M + H]^+$ = 1010.2934. Found $[M + H]^+$ = 1010.2925.

Synthesis of (2S,4R)-1-((S)-2-(4-(1-(6-((5-(1-(2,6-dichlorophenyl)-3-(difluoromethyl)-1H-pyrazol-5-yl)thiazol-2-yl)amino)-6-oxohexyl)-1H-1,2,3-triazol-4-yl)butanamido)-3,3-dimethylbutanoyl)-4-hydroxy-N-(4-(2-methylthiazol-5-yl)benzyl)pyrrolidine-2-carboxamide (**23i; THNAN73**)

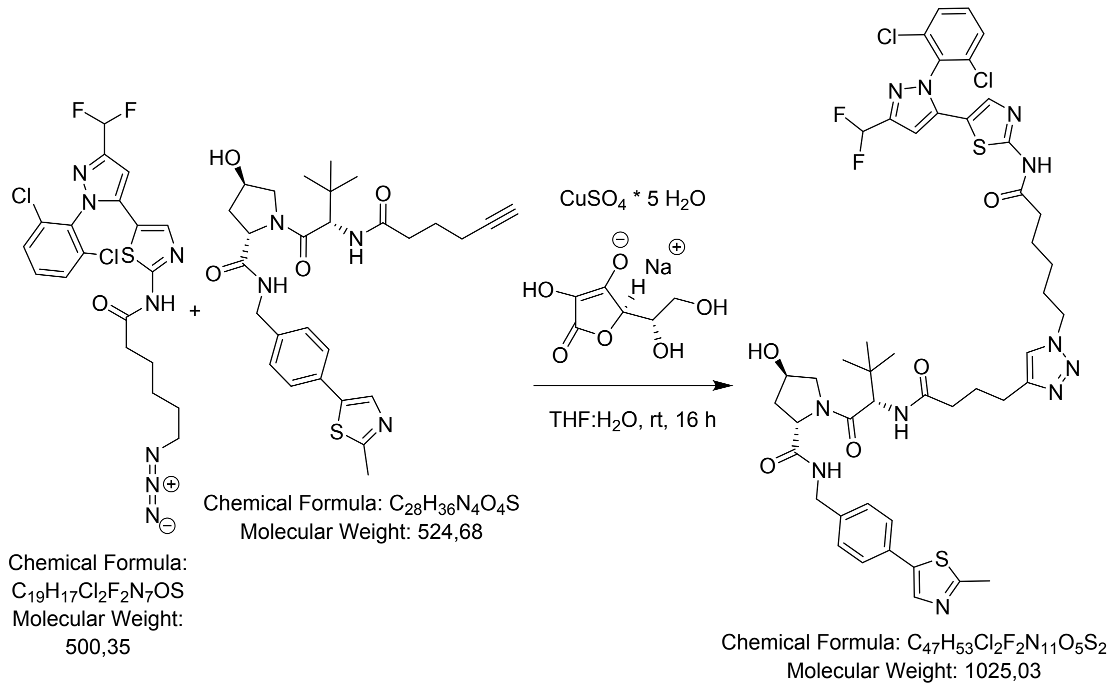

(2*S*,4*R*)-1-((*S*)-2-(hex-5-ynamido)-3,3-dimethylbutanoyl)-4-hydroxy-*N*-(4-(2-methylthiazol-5-yl)benzyl)pyrrolidine-2-carboxamide (**22**) (52 mg, 100 µmol, 1.0 eq.) and 6-azido-*N*-(5-(1-(2,6-dichlorophenyl)-3-(difluoromethyl)-1*H*-pyrazol-5-yl)thiazol-2-yl)hexanamide (**16**) (50 mg, 100 µmol, 1.0 eq.) were added to 6 mL of THF:H_2O (1:1) with copper sulphate pentahydrate (12 mg, 50 µmol, 0.5 eq.) and sodium ascorbate (7 mg, 35 µmol, 0.35 eq.). The reaction mixture was stirred at rt for 16 h. Subsequently, the workup of the reaction consisted of an extraction with 10 mL DCM three times, after which the united organic phases were dried over Na_2SO_4. Lastly, the reaction was purified via flash column chromatography (DCM:EtOH) (98:2→ 0:1). The product was obtained as a brittle, white/green substance.

Theoretical Yield: 102 mg

Yield: 45 mg ≡ 44%

^{1}H NMR (500 MHz, $(CD_3)_2SO$): δ = 12.14 (s, 1H), 8.98 (s, 1H), 8.55 (t, $J_{3H\text{-}H}$ = 6.1 Hz, 1H), 7.97 – 7.69 (m, 5H), 7.66 (s, 1H), 7.45 – 7.35 (m, 4H), 7.22 (s, 1H), 7.12 (t, $J_{2H\text{-}F}$ = 54.3 Hz, 1H), 5.12 (d, $J_{4H\text{-}H}$ = 3.6 Hz, 1H), 4.55 (d, $J_{3H\text{-}H}$ = 9.4 Hz, 1H), 4.47 – 4.40 (m, 2H), 4.35 (s, 1H), 4.27 (t, $J_{3H\text{-}H}$ = 7.1 Hz, 2H), 4.22 (dd, $J_{2H\text{-}H}$ = 15.8, $J_{3H\text{-}H}$ = 5.5 Hz, 1H), 3.70 – 3.60 (m, 2H), 2.89 (s, 2H), 2.44 (s, 3H), 2.37 (t, $J_{3H\text{-}H}$ = 7.4 Hz, 2H), 2.33 – 2.14 (m, 2H), 2.09 – 1.86 (m, 2H), 1.78 (m, 2H), 1.56 (p, $J_{3H\text{-}H}$ = 7.6 Hz, 2H), 1.27 – 1.17 (m, 3H), 0.94 (s, 9H) ppm.

^{13}C NMR (126 MHz, $(CD_3)_2SO$): δ =171.94, 171.77, 171.48, 169.69, 162.30, 159.07, 151.44, 147.71, 146.40, 139.49, 138.44, 138.07, 134.43, 133.84, 133.52, 131.16, 129.64, 129.47, 128.63, 127.42, 121.69, 116.40, 111.05, 103.04, 68.87, 58.70, 56.38, 48.93, 35.77, 35.19, 34.42, 29.40, 26.38, 25.45, 25.38, 24.68, 23.83, 15.93 ppm.

MS (ESI–): *m/z* = 512.75 $[M–2H]^{2–}$

HRMS: m/z Calcd for $C_{47}H_{53}Cl_2F_2N_{11}O_5S_2$ $[M + H]^+$ = 1024.3090. Found $[M + H]^+$ = 1024.3086.

REFERENCES:

1. Schrödinger, LLC (2024). The PyMOL Molecular Graphics System. Version 3.1.0a0.

2. Waterhouse, A.M., Procter, J.B., Martin, D.M.A., Clamp, M., and Barton, G.J. (2009). Jalview Version 2—a multiple sequence alignment editor and analysis workbench. Bioinformatics *25*, 1189–1191. https://doi.org/10.1093/bioinformatics/btp033.
